# Mechanisms and applications of cyclometalated Pt(ii) complexes in photoredox catalytic trifluoromethylation†
†Electronic supplementary information (ESI) available: Experimental details and the syntheses of the Pt(ii) catalysts; Fig. S1–S55, showing the phosphorescence spectra, femto- and nanosecond laser flash photolysis results, cyclic and differential pulse voltammograms, phosphorescence quenching experiment results, photoinduced ESR spectrum, spectroelectrochemical UV-vis-NIR absorption spectrum, dark-state reaction results, and ^1^H, ^13^C, and ^19^F NMR spectra; Scheme S1, presenting the synthetic routes to the Pt(ii) complexes; Tables S1–S6, listing optimization results for the trifluoromethylation of 1-dodecene, 3-methylindole and *N*-methylpyrrole, and the Cartesian coordinates for the optimized geometries of one-electron oxidized species of the Pt complexes. See DOI: 10.1039/c4sc02537g



**DOI:** 10.1039/c4sc02537g

**Published:** 2014-11-24

**Authors:** Won Joon Choi, Sungkyu Choi, Kei Ohkubo, Shunichi Fukuzumi, Eun Jin Cho, Youngmin You

**Affiliations:** a Department of Advanced Materials Engineering for Information and Electronics , Kyung Hee University , Yongin , Gyeonggi-do 446-701 , Korea . Email: odds2@khu.ac.kr; b Department of Applied Chemistry & Department of Bionanotechnology , Hanyang University , Ansan , Gyeonggi-do 426-791 , Korea . Email: echo@hanyang.ac.kr; c Department of Material and Life Science , Graduate School of Engineering , Osaka University , ALCA , Japan Science and Technology Agency (JST) , Suita , Osaka 565-0871 , Japan . Email: fukuzumi@chem.eng.osaka-u.ac.jp

## Abstract

The incorporation of a trifluoromethyl group into an existing scaffold can provide an effective strategy for designing new drugs and agrochemicals. Among the numerous approaches to trifluoromethylation, radical trifluoromethylation mediated by visible light-driven photoredox catalysis has gathered significant interest as it offers unique opportunities for circumventing the drawbacks encountered in conventional methods. A limited understanding of the mechanism and molecular parameters that control the catalytic actions has hampered the full utilization of photoredox catalysis reactions. To address this challenge, we evaluated and investigated the photoredox catalytic trifluoromethylation reaction using a series of cyclometalated Pt(ii) complexes with systematically varied ligand structures. The Pt(ii) complexes were capable of catalyzing the trifluoromethylation of non-prefunctionalized alkenes and heteroarenes in the presence of CF_3_I under visible light irradiation. The high excited-state redox potentials of the complexes permitted oxidative quenching during the cycle, whereas reductive quenching was forbidden. Spectroscopic measurements, including time-resolved photoluminescence and laser flash photolysis, were performed to identify the catalytic intermediates and directly monitor their conversions. The mechanistic studies provide compelling evidence that the catalytic cycle selects the oxidative quenching pathway. We also found that electron transfer during each step of the cycle strictly adhered to the Marcus normal region behaviors. The results are fully supported by additional experiments, including photoinduced ESR spectroscopy, spectroelectrochemical measurements, and quantum chemical calculations based on time-dependent density functional theory. Finally, quantum yields exceeding 100% strongly suggest that radical propagation significantly contributes to the catalytic trifluoromethylation reaction. These findings establish molecular strategies for designing trifluoromethyl sources and catalysts in an effort to enhance catalysis performance.

## Abstract 2

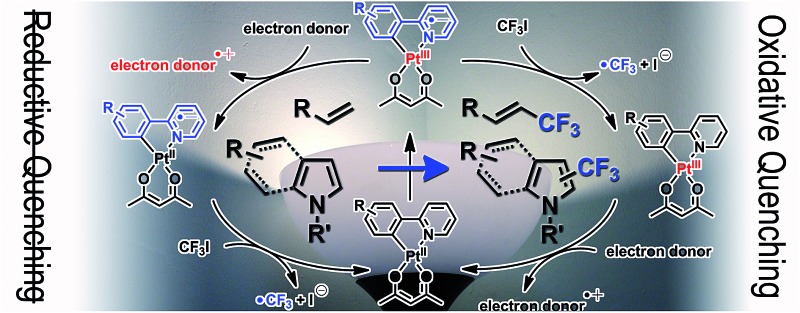
Pt(ii) complexes catalyse the visible light-driven trifluoromethylation of alkenes and heteroarenes with improved quantum yields, due to strict adherence to an oxidative quenching pathway.

## Introduction

The recent development of visible light-driven photoredox catalysis has attracted renewed interest in radical-mediated organic syntheses.^1–21^ A wide variety of radical transformations that have been difficult to accomplish using conventional initiators can be carried out through photoredox catalysis. The most notable example of photoredox catalysis, which highlights the value of the reaction, is trifluoromethylation. While trifluoromethylation significantly improves the activities of drugs and agrochemicals, such as their metabolic stabilities, binding selectivities, and bioavailabilities,^22–26^ its high electron deficiency precludes reactions based on substitution methodologies. Trifluoromethylation has thus relied on the use of toxic transition metal catalysts, such as those containing Pd^27–37^ and Cu.^38–51^ Photoredox catalytic trifluoromethylation provides a promising alternative to these existing methods, as it utilizes the environmentally benign photon.^52^ The mild reaction conditions tolerate a variety of functional groups and, thus, are amenable to the post-modification of bioactive molecules.^53–55^

**Scheme 1:**
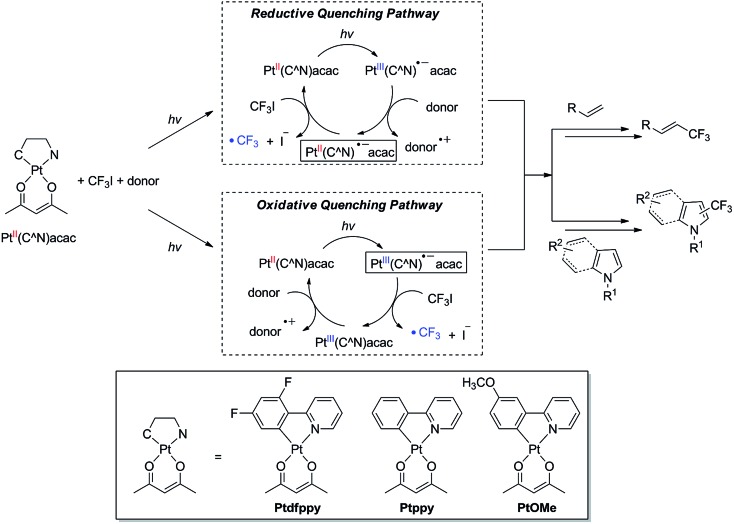
Photoredox catalytic trifluoromethylation of alkenes and heteroarenes.

Photoredox catalysed trifluoromethylation reactions typically employ coordinatively saturated 4d or 5d transition metal complexes, such as Ru(ii) polypyridine complexes^53–66^ and cyclometalated Ir(iii) complexes,^64,67–70^ as the catalysts. Photoexcitation of these complexes promotes the formation of a long-lived triplet metal-to-ligand charge-transfer (^3^MLCT) transition state, in which the one-electron oxidized metal core and the one-electron reduced ligand act as the oxidant and reductant, respectively. A variety of reagents, such as CF_3_I,^55,60–62,64,67,71,72^ silver trifluoroacetate,^73^ triflyl chloride (CF_3_SO_2_Cl),^54,69^ the Langlois reagent,^74^ the Togni reagent,^56,58,59,65,70^ the Umemoto reagent,^53,57,63,68,70^ and the Shibata reagent,^66^ can serve as trifluoromethyl radical (˙CF_3_) sources,^75^ and the key step involves the reductive cleavage of these reagents under photoirradiation of the catalyst. Two possible routes to the reductive generation of ˙CF_3_ are available (Scheme 1). The photoexcited catalyst can directly deliver an electron to the ˙CF_3_ source (oxidative quenching pathway), followed by the reductive regeneration of the catalyst by an electron donor. Alternatively, the photoexcited catalyst may be preferentially reduced by an electron donor to a one-electron reduced species that subsequently transfers an extra electron to the ˙CF_3_ source (reductive quenching pathway). Although the net reactions are identical (*i.e.*, CF_3_X + donor → ˙CF_3_ + donor˙^+^ + X^–^), each of two pathways involves reductants with distinct stabilities and thermodynamic driving forces for reductive cleavage. As shown in Scheme 1, the oxidative and reductive quenching pathways involve the photoexcited catalyst and one-electron reduced catalyst, respectively, as the reductants. In most cases, photoredox catalysts, such as [Ru(bpy)_3_]^2+^ and *fac*-Ir(ppy)_3_ (bpy = 2,2′-bipyridine and ppy = 2-phenylpyridinate), can promote both oxidative^53,54,56–59,63,65,68–70^ and reductive^55,60–62,64,67,74^ quenching cycles, due to the positive driving forces for electron transfer to typical ˙CF_3_ sources and from electron donors.^7^ The presence of two competing pathways hampers the elucidation and optimization of the molecular parameters associated with the catalysis. Further developments are needed to identify photoredox catalysts that select a single quenching process. This need prompted us to employ cyclometalated Pt(ii) complexes as photoredox catalysts for trifluoromethylation reactions. The cyclometalated structure between a Pt(ii) core and an anionic cyclometalating (C∧N) ligand can cathodically shift the excited-state redox potentials. This effect provides the thermodynamic conditions needed to suppress excited-state reduction while enabling excited-state oxidation of the Pt complex. Thus, the resulting photoredox catalysis reaction strictly follows an oxidative quenching cycle. The additional benefits of using Pt(ii) complexes are enormous. The complexes can be easily modified, allowing the molecular parameters to be synthetically tailored. Control over the substituents in the C∧N ligands provides a viable approach to tuning the excited-state redox potentials and lifetimes of the complexes. Molecular design efforts can benefit from the rich knowledge established in previous studies of Pt(ii) complexes in the context of electrophosphorescence and sensors.^76–79^ Taking advantage of these properties, photoredox catalytic applications of Pt(ii) complexes in polymerization,^80,81^ hydrogen production,^82^ and oxidation reactions^83,84^ have been demonstrated. It is, however, noted that trifluoromethylation by a Pt(ii) complex has yet to be established.

Here, we report the first demonstration of photoredox catalytic trifluoromethylation using cyclometalated Pt(ii) complexes. Key intermediates in the photoredox catalytic cycle were monitored directly using spectroscopic techniques. The spectroscopic identification of catalytic intermediates in the visible light-driven trifluoromethylation reaction is unprecedented. Mechanistic studies revealed that the photoredox catalysis involves an oxidative quenching pathway. The kinetic parameters associated with each step of the catalytic cycle were determined, providing novel insights into enhancing the catalysis performance.

## Results and discussion

### Synthesis and properties of the Pt(ii) complex catalysts

A series of prototypical Pt(ii) complexes having C∧N ligands with different electron densities, 2-(2,4-difluorophenyl)pyridinate, 2-phenylpyridinate, and 2-(3-methoxyphenyl)pyridinate (Ptdfppy, Ptppy, and PtOMe, respectively, in Scheme 1), were tested here.^85^ The ligand environment around the Pt(ii) center was completed by non-photoactive acetylacetonate ancillary ligands. The complexes were prepared through a modified procedure based on the protocol reported by Zhao and co-workers.^86^ All compounds were characterized using standard spectroscopic methods, including ^1^H, ^13^C, and ^19^F NMR spectroscopy, mass spectrometry, and elemental analysis. The spectroscopic identification data agreed fully with the proposed structures (see ESI†).

**Fig. 1:**
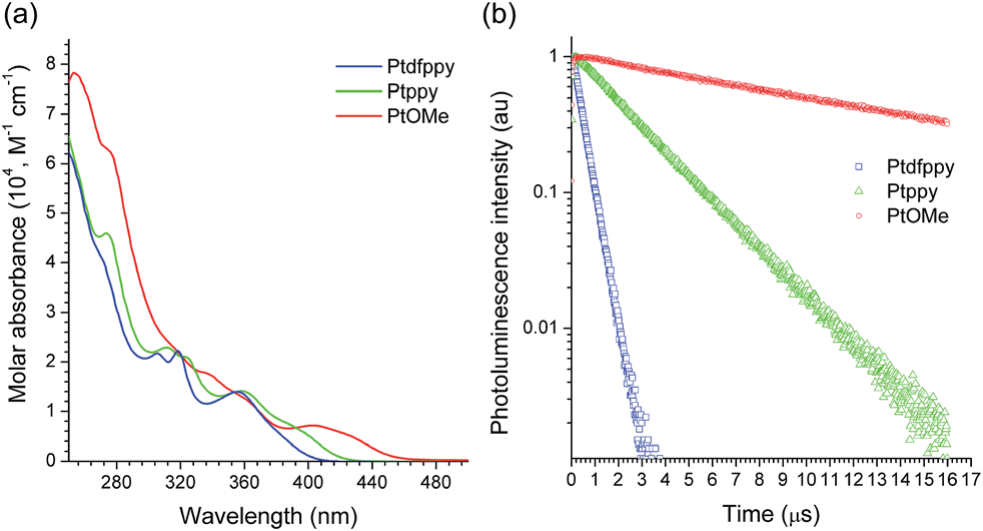
(a) UV-vis absorption spectra of the Pt(ii) complexes (10 μM in acetonitrile). (b) Photoluminescence decay traces of the Pt(ii) complexes (50 μM in deaerated acetonitrile) after nanosecond pulsed photoexcitation at 377 nm: *λ*_obs_ = 465 nm (Ptdfppy), 483 nm (Ptppy), and 543 nm (PtOMe).

The UV-vis absorption spectra of the Pt(ii) complexes (10 μM in CH_3_CN) featured characteristic MLCT transition bands at 380 nm (log *ε* = 3.76; Ptdfppy), 386 nm (log *ε* = 3.91; Ptppy), and 420 nm (log *ε* = 3.78; PtOMe), with tails that approached the visible regions (Fig. 1a).^85,87,88^ Strong photoluminescence emission was observed upon photoexcitation of the MLCT bands at room temperature (see ESI, Fig. S1† for the photoluminescence spectra). The photoluminescence lifetimes (*τ*_obs_) of the emission were on the order of submicroseconds or microseconds, indicating phosphorescence (Fig. 1b). It should be noted that the *τ*_obs_ value increased by two orders of magnitude as the electron richness of the C∧N ligand was increased (*i.e.*, in PtOMe). The slow decay was indicative of weak MLCT contributions to the spin-forbidden electronic transition. Indeed, the MLCT character estimated by time-dependent density functional theory (CPCM(CH_3_CN)-TD-B3LYP/LANL2DZ:6-311+G(d,p)//B3LYP/LANL2DZ:6-311+G(d,p)) was significantly smaller in the PtOMe complex (15%) than in the Ptdfppy complex (28%) (Table 1). Therefore, the triplet state responsible for phosphorescence emission can be best described as an admixture of the MLCT, ligand-centered (LC) π–π*, and intraligand charge-transfer (ILCT) transitions. The large variations in *τ*_obs_ were useful for examining the notion of whether a long-lifetime catalyst could exhibit better photoredox catalytic performance (*vide infra*). The photophysical data, including the photoluminescence quantum yields (PLQY) and the triplet-state energies (Δ*E*_T_) of the Pt(ii) complexes are summarized in Table 1.

**Table 1:** Photophysical and electrochemical data for the photoredox catalysts

	λ_abs_ ^*a*^ (MLCT) (nm; log *ε*)	PLQY^*b*^	MLCT^*c*^ (%)	Δ*E*_T_ ^*d*^ (eV)	*E*_ox_ ^*e*^ (V *vs.* SCE)	*E*_red_ ^*f*^ (V *vs.* SCE)	*E* * ox ^*g*^ (V *vs.* SCE)	*E* * red ^*h*^ (V *vs.* SCE)	*τ*_obs_ ^*i*^ (μs)
Ptdfppy	380 (3.76)	0.31	28	2.73	0.62 (qr)	–2.46	–2.11	0.27	0.382 ± 0.059
Ptppy	386 (3.91)	0.44	27	2.64	0.57 (irr)	–2.38	–2.07	0.26	2.21 ± 0.14
PtOMe	420 (3.78)	0.74	15	2.45	0.52 (irr)	–2.20	–1.93	0.25	11.6 ± 0.50

^*a*^10 μM in acetonitrile solutions, 298 K.

^*b*^Photoluminescence quantum yields determined relative to the fluorescein standard (0.1 N NaOH (aq), PLQY = 0.79).

^*c*^MLCT contribution to the triplet transition estimated using AOMix based on the TD-DFT (CPCM(CH_3_CN)-TD-B3LYP/LANL2DZ:6-311+G(d,p)//B3LYP/LANL2DZ:6-311+G(d,p)) results.

^*d*^Triplet-state energy determined from the phosphorescence spectra.

^*e*^Determined using cyclic and differential pulse voltammetry. Conditions: scan rate = 100 and 0.4 mV s^–1^ for CV and DPV, respectively; 1.0 mM Pt complex in Ar-saturated acetonitrile containing the 0.10 M Bu_4_NPF_6_ supporting electrolyte; a Pt wire counter electrode and a Pt microdisc working electrode; the Ag/AgNO_3_ couple as the pseudo-reference electrode; qr = quasi-reversible; irr = irreversible.

^*f*^Estimated using *E*_red_ = *E*_ox_ – Δ*E*_g_, where Δ*E*_g_ is the optical band gap energy: Ptdfppy, 3.08 eV; Ptppy, 2.95 eV; PtOMe, 2.72 eV.

^*g*^ *E* * ox = *E*_ox_ – Δ*E*_T_.

^*h*^ *E* * red = *E*_red_ + Δ*E*_T_.

^*i*^Photoluminescence lifetimes observed at *λ*_em_ = 465 nm (Ptdfppy), 483 nm (Ptppy), and 543 nm (PtOMe). The measurements were collected in triplicate.

The ground-state redox potentials of the Pt(ii) complexes were determined by cyclic voltammetry (CV), and were further verified using differential pulse voltammetry (DPV). Quasi-reversible (Ptdfppy) and irreversible (Ptppy and PtOMe) oxidation waves were measured at 0.52–0.62 V *vs.* SCE (see ESI, Fig. S2† for the voltammograms), corresponding to the Pt^III/II^ redox couple.^88^ These oxidation potentials (*E*_ox_) were more positive than the *E*_ox_ value (0.47 V *vs.* SCE) of *N*,*N*,*N*′,*N*′-tetramethylethylenediamine (TMEDA), a sacrificial electron donor. Because reduction was not observed until –1.8 V *vs.* SCE, the reduction potentials (*E*_red_) for the Pt(ii) complexes were calculated according to the relationship, *E*_red_ = *E*_ox_ – Δ*E*_g_, where Δ*E*_g_ is the optical band gap energy. As summarized in Table 1, the *E*_red_ value shifted cathodically as the electron density of the C∧N ligand decreased. The same trend was observed in the excited-state oxidation potential (E*ox = *E*_ox_ – Δ*E*_T_), whereas the excited-state reduction potential (E*red = *E*_red_ + Δ*E*_T_) remained relatively unchanged across the series of the Pt(ii) complexes (Table 1). The changes in the E*ox values were dominated by Δ*E*_T_ rather than *E*_ox_, as inferred from ΔΔ*E*_T_ (0.28 eV) > *e*Δ*E*_ox_ (0.10 eV; *e* is the elementary charge). The E*ox values (–2.11 to –1.93 V *vs.* SCE) were more negative than the *E*_red_ value of CF_3_I (–0.91 V *vs.* SCE,^89^ determined by DPV (ESI, Fig. S3†)), indicating that the photoexcited Pt complexes could donate an electron to CF_3_I under the driving force for the photoinduced electron transfer (–Δ*G*_PeT_ = *e*[E*ox(Pt) – *E*_red_(CF_3_I)]) of 1.02–1.20 eV. The photoexcited complexes could not be reduced by TMEDA, as indicated by the negative driving force (–Δ*G*_PeT_ = *e*[*E*_ox_(TMEDA) – E*red(Pt)] = –0.20∼ –0.22 eV).

### Trifluoromethylation of non-prefunctionalized alkenes and heteroarenes

**Fig. 2:**
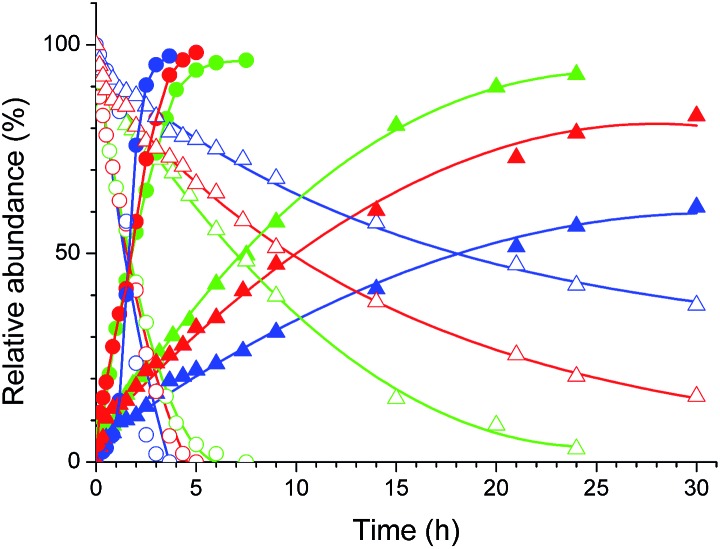
Photoredox catalytic trifluoromethylation of 1-dodecene (circles) and *N*-methylpyrrole (triangles) by Ptdfppy (blue), Ptppy (green), and PtOMe (red). Empty and filled symbols correspond to the substrates and the trifluoromethylated products, respectively. Conditions: 2.0 mL of a deaerated acetonitrile solution containing 0.50 mmol substrate, 0.010 mmol Pt catalyst, 1.0 mmol TMEDA (or 1.0 mmol DBU), and 1.5 mmol CF_3_I was photoirradiated under blue LEDs (450 nm, 7 W) at room temperature. The progress of the reaction was monitored using gas chromatography, with dodecane as the internal standard.

The Pt(ii) complexes were evaluated for their activity in the photoredox catalytic trifluoromethylation of non-prefunctionalized sp^2^ carbons. We employed CF_3_I as a ˙CF_3_ source. Trifluoromethylation of 0.50 mmol terminal alkenes was carried out in a deaerated acetonitrile solution (2.0 mL) containing 1.0 mol% Pt catalyst, 1.0 mmol DBU and 1.5 mmol CF_3_I. The reaction mixture was photoirradiated using blue LEDs (450 nm, 7 W) at room temperature, and the progress of the reaction was monitored using gas chromatography or thin-layer chromatography. The same method was applied to the trifluoromethylation of heteroarenes, except that 1.0 mmol TMEDA was used in place of DBU. The reaction conditions were optimized by testing several bases, DIPEA, TEA, K_3_PO_4_, K_2_HPO_4_, and KO^*t*^Bu, and by testing several solvents, DMF, CH_3_OH, and CH_2_Cl_2_. The optimization results revealed that the protocols described above worked best (ESI, Tables S1 and S2†). As demonstrated in Fig. 2, the trifluoromethylation of 1-dodecene and *N*-methylpyrrole went to completion within 6 h and 30 h, respectively. The trifluoromethylated products were not detected without the Pt catalyst nor photoirradiation (ESI, Tables S1–S3†), confirming that the photoexcited catalysts were responsible for the observed reactivities. Under the optimized conditions, the Pt(ii) complexes exhibited catalytic activities comparable to the well-established photoredox catalysts, such as [Ru(bpy)_3_]Cl_2_ and [Ir(ppy)_2_(dtbbpy)]PF_6_ (dtbbpy = 4,4′-di(*t*-butyl)-2,2′-bipyridine). The scope of the Pt(ii) complex-mediated trifluoromethylation was investigated over a range of alkenes and heteroarenes (Tables 2 and 3). The isolated yields of the trifluoromethylated alkenes exceeded 82%, whereas moderate yields were obtained from the trifluoromethylation of heteroarenes. The trifluoromethylation reaction was highly tolerant of the presence of a variety of functional groups, including hydroxyl (**2c**), silylether (**2d**), ester (**2e**, **2f** and **4b**), amide (**2g** and **2h**), carbonate (**2i**), and sulfonate (**2j**) groups. Spectroscopic data for the trifluoromethylated alkenes and heteroarenes listed in Tables 2 and 3 are summarized in the ESI.† These results successfully demonstrate that the cyclometalated Pt(ii) complexes are promising alternatives to conventional photoredox catalysts based on Ir(iii) and Ru(ii) complexes for trifluoromethylation.

**Table 2:** Photoredox catalytic trifluoromethylation of alkenes^*a*^

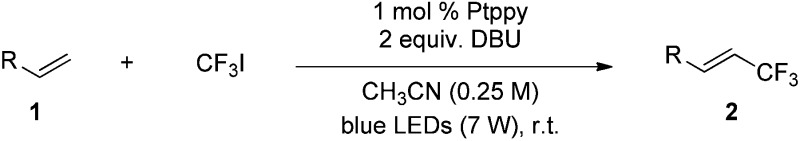			
Entry	Product		Yield^*b*^ (%; *E/Z* ratio^*c*^)
1	**2a**	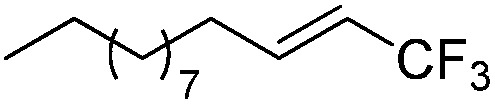	97 (25 : 1)
2	**2b**	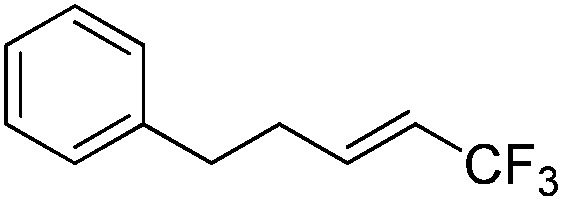	82 (25 : 1)
3	**2c**	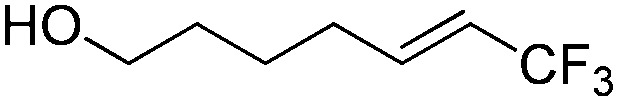	85 (30 : 1)
4	**2d**	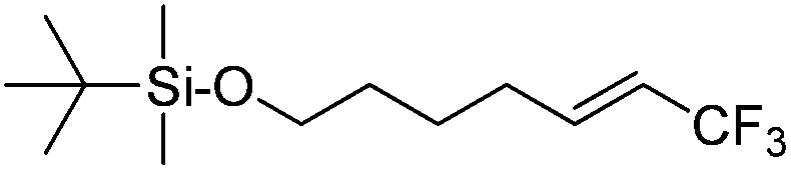	95 (25 : 1)
5	**2e**	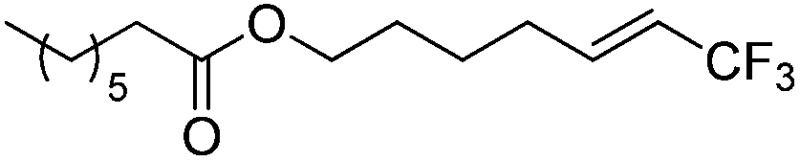	95 (25 : 1)
6	**2f**	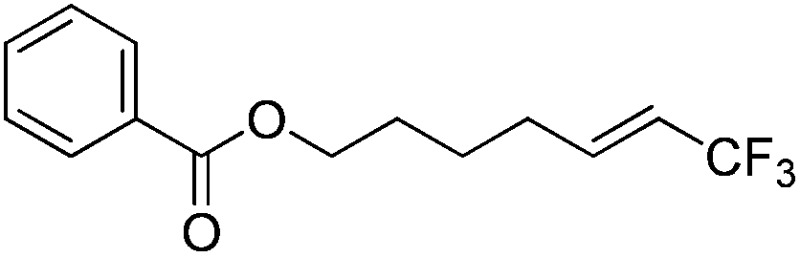	94 (25 : 1)
7	**2g**	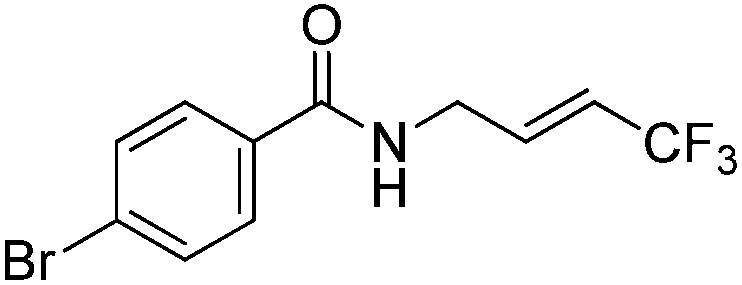	88 (only *E*)
8	**2h**	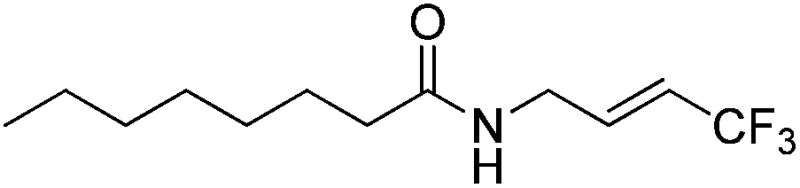	89 (40 : 1)
9	**2i**	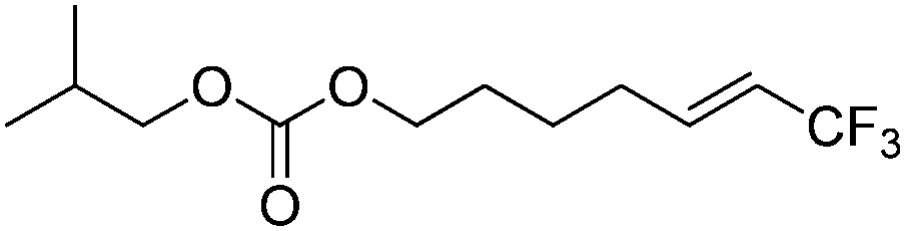	96 (25 : 1)
10	**2j**	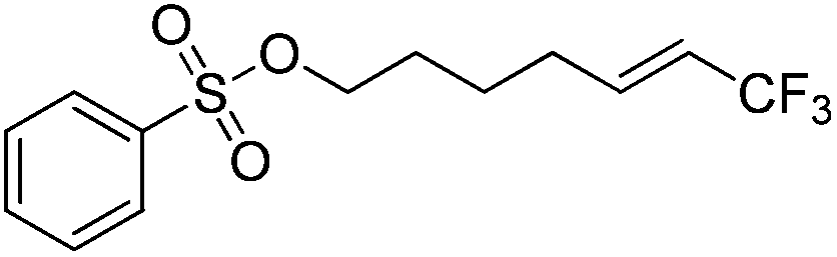	89 (25 : 1)

^*a*^An oven-dried resealable test tube equipped with a magnetic stirrer bar was charged with an alkene (0.50 mmol), sealed with a screw-cap, and degassed by alternating between putting under vacuum and backfilling with argon. A solution of Ptppy (1.0 mol%, 0.0050 mmol) in CH_3_CN (2.0 mL, 0.25 M) and DBU (1.0 mmol) was then added to the tube under argon. CF_3_I (1.5 mmol) was then delivered to the reaction mixture using a gastight syringe. The test tube was placed under blue LEDs (7 W) at room temperature for 5–10 h, and the progress of each reaction was monitored by TLC or gas chromatography.

^*b*^The given yields are isolated yields reported as the average of two runs, except for **2b** (entry 2), which was monitored using ^19^F NMR due to the volatility of the product.

^*c*^The *E*/*Z* ratio was determined by ^1^H NMR spectroscopy and gas chromatography.

**Table 3:** Photoredox catalytic trifluoromethylation of heteroarenes^*a*^

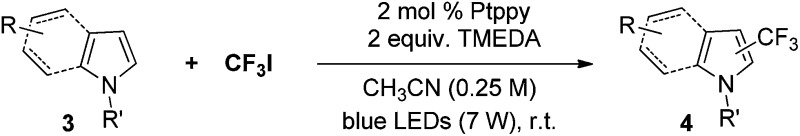			
Entry	Product		Yield^*b*^ (%)
1	**4a**	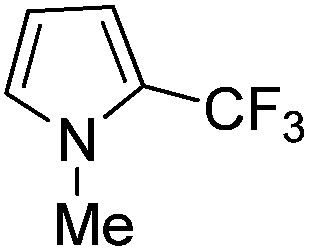	95
2	**4b**	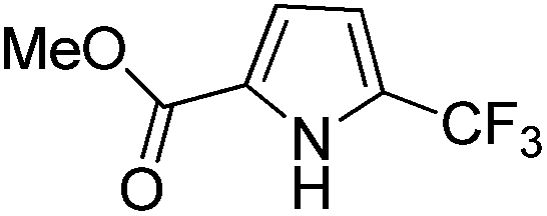	75
3	**4c**	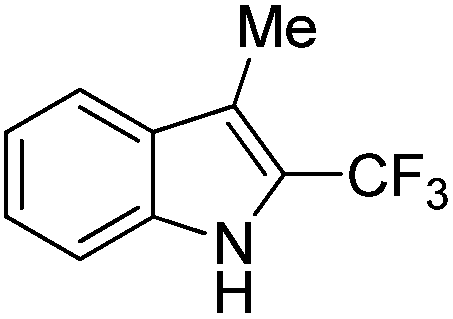	70
4	**4d**	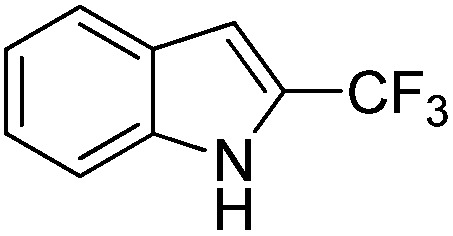	68
5	**4e**	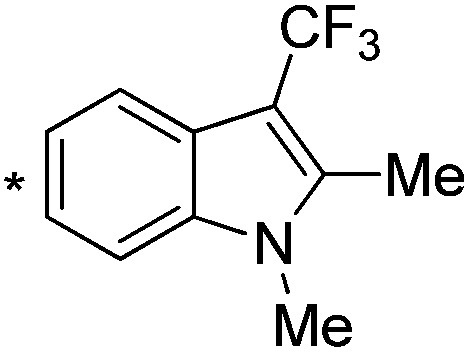	67^*c*^

^*a*^An oven-dried resealable test tube equipped with a magnetic stirrer bar was charged with an acetonitrile solution (2.0 mL) containing heteroarene (0.50 mmol), TMEDA (1.0 mmol), and Ptppy (2.0 mol%, 0.010 mmol), and sealed with a screw-cap. The reaction mixture was degassed by alternating between putting under vacuum and backfilling with argon, after which CF_3_I (1.5 mmol) was delivered using a gastight syringe. The test tube was placed under blue LEDs (7 W) at room temperature for 10–24 h, while the progress of each reaction was monitored by TLC.

^*b*^The given yields are isolated yields obtained by taking the average of two runs, except for **4a** (entry 1), which was monitored using ^19^F NMR due to the volatility of the product.

^*c*^Trifluoromethylation at the phenyl moiety of indole occurred at <10% (^19^F NMR and GC).

**Scheme 2:**
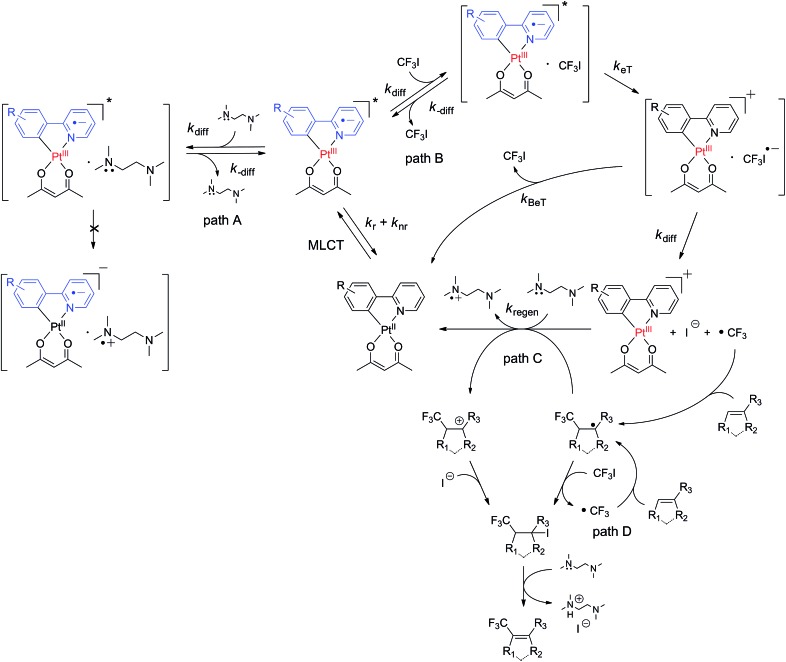
Proposed mechanism for the trifluoromethylation of alkenes and heteroarenes.

Scheme 2 illustrates the proposed mechanism underlying the photoredox catalytic trifluoromethylation reaction in the presence of the cyclometalated Pt(ii) complex (Pt^II^(C∧N) hereafter) catalysts. Photoexcitation of Pt^II^(C∧N) promoted an electronic transition to a singlet MLCT (^1^MLCT) state, which underwent ultrafast intersystem crossing to a ^3^MLCT state (*i.e.*, ^3^[Pt^III^(C∧N)˙^–^]*). Diffusional collision of ^3^[Pt^III^(C∧N)˙^–^]* with CF_3_I led to the reversible formation of an encounter complex ([^3^[Pt^III^(C∧N)˙^–^]* CF_3_I]; path B in Scheme 2). Oxidative electron transfer from the Pt catalyst to CF_3_I in the encounter complex generated a geminate radical ion pair, [[Pt^III^(C∧N)]^+^ CF_3_I˙^–^]. It should be noted that ligand oxidation (*i.e.*, [Pt^II^(C∧N)˙^+^]^+^) could not be excluded, because the triplet state of the Pt(ii) complex contained significant LC character (*vide supra*). An encounter complex formed between the photoexcited catalyst and an electron donor, such as TMEDA, could also have been generated (path A in Scheme 2); however, the negative driving force abrogated the formation of a geminate radical ion pair (*vide supra*). Two pathways were available to the [[Pt^III^(C∧N)]^+^ CF_3_I˙^–^] species: the first pathway involved quenching to the original species (*i.e.*, Pt^II^(C∧N) and CF_3_I) by back electron transfer (BeT), and the other pathway involved dissociation into [Pt^III^(C∧N)]^+^ and CF_3_I˙^–^. In the latter path, prompt cleavage of the C–I bond in CF_3_I˙^–^ resulted in the formation of ˙CF_3_. Radical addition of ˙CF_3_ to substrates formed trifluoromethylated radical species. The photocatalytic cycle was completed by the reductive regeneration of Pt^II^(C∧N) with sacrificial electron donors, such as TMEDA and DBU. Alternatively, Pt^II^(C∧N) was recovered through the radical-polar mechanism, which involved oxidation of radical species of the trifluoromethylated substrate to the cation (path C in Scheme 2). In this case, the resulting cationic species of the trifluoromethylated substrate was trapped by the strong nucleophile, I^–^. It should be noted that the compound bearing both iodide and trifluoromethyl groups could also be produced by radical propagation between the CF_3_I and radical species of the trifluoromethylated substrate (path D in Scheme 2). In both cases (*i.e.*, paths C and D), E2 elimination assisted by TMEDA or DBU furnished the desired product, with the stoichiometric generation of ammonium iodide salts.

The photoredox catalytic cycle described above involved three key electron-transfer steps: forward photoinduced electron transfer (PeT), BeT, and reductive regeneration of the catalyst. The electron-transfer processes and their intermediate species generated during the photoredox catalytic trifluoromethylation reaction had not been directly observed to date. No kinetic information about electron transfer was available in the literature.

### Spectroscopic observation of the photoredox catalytic cycle

**Fig. 3:**
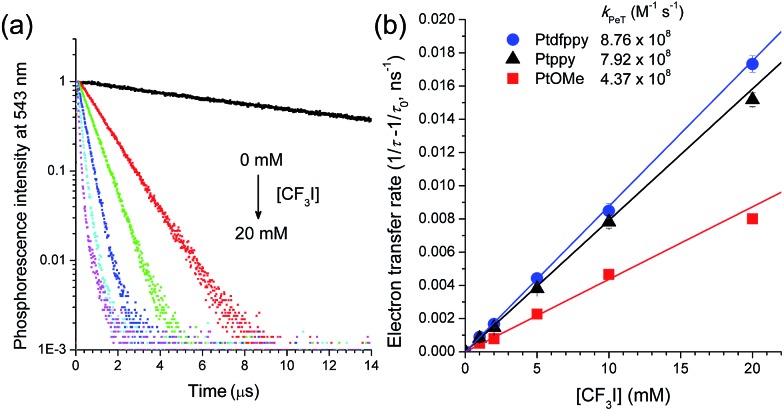
Determining the rate constants for photoinduced electron transfer (*k*_PeT_). (a) Phosphorescence decay traces of 50 μM PtOMe (deaerated CH_3_CN; *λ*_obs_ = 543 nm) over the range of CF_3_I concentrations (0–20 mM). (b) Plot of the electron transfer rates (1/*τ*_obs_(CF_3_I) – 1/*τ*_obs_, *τ*_obs_(CF_3_I) and *τ*_obs_ are the phosphorescence lifetimes of the Pt(ii) complexes in the presence and absence of CF_3_I, respectively) as a function of the concentration of CF_3_I. The *k*_PeT_ values were determined based on the pseudo-first order fits of the electron transfer rates. Standard deviations were determined from three independent measurements.

The electron transfer reactions in each step of the photoredox catalytic cycle were investigated using time-resolved photoluminescence and laser flash photolysis measurements after nanosecond pulsed photoexcitation. Photoluminescence quenching experiments using time-correlated single photon counting (TCSPC) techniques were performed to monitor the oxidative PeT from ^3^[Pt^III^(C∧N)˙^–^]* to CF_3_I. Attempts to detect the rise signals of the transient absorption spectrum of the oxidized catalyst, [Pt^III^(C∧N)]^+^, were unsuccessful, because the significant overlap between the absorption spectra of [Pt^III^(C∧N)]^+^ and ^3^[Pt^III^(C∧N)˙^–^]* (ESI, Fig. S4†) did not permit spectral resolution. Fig. 3a shows the photoluminescence decay traces (*λ*_obs_ = 543 nm) for the deaerated acetonitrile solutions of 50 μM PtOMe after nanosecond pulsed photoexcitation at 377 nm. The decay trace followed a monoexponential decay model. *τ*_obs_ was as long as 11.6 μs, but the incremental addition of CF_3_I (0–20 mM) to the solution significantly shortened *τ*_obs_. Other Pt complexes show similar photoluminescence quenching behaviors (ESI, Fig. S5†). Photoluminescence quenching accompanied the generation of ˙CF_3_, as evidenced by a weak photoinduced ESR signal with a *g* value of 2.004, corresponding to a free radical,^90,91^ although the broad ESR spectrum hindered resolution of the hyperfine coupling due to the fluorine nuclei (ESI, Fig. S6†). These results unambiguously indicate the occurrence of oxidative quenching of ^3^[Pt^III^(C∧N)˙^–^]* by CF_3_I. As inferred from the negative driving force (*vide supra*), reductive quenching was not observed, even at 20 mM TMEDA (ESI, Fig. S7†). The oxidative PeT rates were calculated from 1/*τ*_obs_(CF_3_I) – 1/*τ*_obs_, where *τ*_obs_(CF_3_I) and *τ*_obs_ are the photoluminescence lifetimes in the presence and absence of CF_3_I, respectively. The rate constants of PeT (*k*_PeT_), determined by the pseudo-first order fit of the PeT rates *vs.* the CF_3_I concentrations, were 8.8 × 10^8^ M^–1^ s^–1^, 7.9 × 10^8^ M^–1^ s^–1^, and 4.4 × 10^8^ M^–1^ s^–1^ for Ptdfppy, Ptppy, and PtOMe, respectively (Fig. 3b). Apparently, *k*_PeT_ increased in proportion to –Δ*G*_PeT_, indicating electron transfer in the Marcus normal region (*vide infra*). The fraction (*f*) of ^3^[Pt^III^(C∧N)˙^–^]* that underwent oxidative quenching was estimated based on the empirical relationship, *f* = (*k*_PeT_ × 1 mM)/(*k*_r_ + *k*_nr_ + *k*_PeT_ × 1 mM), assuming that 1 mM CF_3_I was present and other conditions, including diffusion, were held constant. In this relationship, *k*_r_ and *k*_nr_ are the rate constants for the radiative transition and non-radiative transition, respectively, which compete with PeT (Table 4). The corresponding *f* values are 0.25, 0.74, and 0.84 for Ptdfppy, Ptppy, and PtOMe, respectively. This trend agreed with the order of *τ*_obs_, suggesting that a photocatalyst with a long excited-state lifetime was likely to undergo oxidative quenching.

**Table 4:** Rate constants for the photophysical and photoelectrochemical processes of the Pt(ii) complexes

	*k*_r_ ^*a*^ (10^4^ s^–1^)	*k*_nr_ ^*b*^ (10^4^ s^–1^)	*k*_PeT_ ^*c*^ (10^8^ M^–1^ s^–1^)	*k*_BeT_ ^*d*^ (10^10^ M^–1^ s^–1^)	*k*_regen_ ^*e*^ (10^8^ M^–1^ s^–1^)
Ptdfppy	81.2	181	8.8	2.2	7.7
Ptppy	1.99	25.3	7.9	1.4	5.0
PtOMe	6.38	2.24	4.4	0.57	3.4

^*a*^Radiative rate constant.

^*b*^Non-radiative rate constant.

^*c*^Rate constant for photoinduced electron transfer.

^*d*^Rate constant for back electron transfer.

^*e*^Rate constant for regeneration of the Pt(ii) complex photocatalyst by reductive electron transfer from TMEDA.

**Fig. 4:**
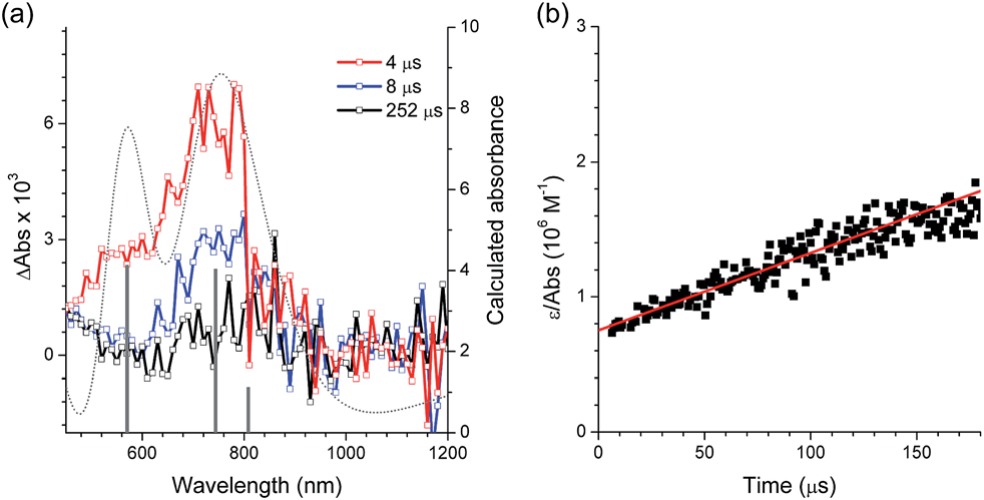
Determination of the rate constant for back electron transfer (*k*_BeT_) by nanosecond laser flash photolysis (*λ*_ex_ = 355 nm) for a deaerated acetonitrile solution containing 100 μM PtOMe (O.D. at 355 nm = 0.52) and 50 mM CF_3_I. (a) Transient absorption spectra recorded at 4 μs (red), 8 μs (blue), and 252 μs (black) after photoexcitation. The dotted grey line and grey bars are the simulated (CPCM(CH_3_CN)-TD-B3LYP/LANL2DZ:6-311+G(d,p)//B3LYP/LANL2DZ:6-311+G(d,p)) absorption spectrum and calculated absorbance, respectively, of the one-electron oxidized species of PtOMe. (b) Plot of *ε*/absorbance of the 770 nm band *vs.* time. The *k*_BeT_ value was determined from the second-order linear fit (red). The results obtained from other Pt(ii) complexes are shown in the ESI, Fig. S8.†

After oxidative quenching by PeT, BeT could occur in the geminate radical ion pair to restore [Pt^II^(C∧N)] and CF_3_I. The BeT process was monitored according to the [Pt^III^(C∧N)]^+^ decay signals in the microsecond regime. Fig. 4a presents the transient absorption spectra of a deaerated acetonitrile solution containing 100 μM PtOMe and 50 mM CF_3_I after nanosecond photoexcitation at 355 nm (O.D. = 0.52). The spectra display a visible absorption band at 770 nm, and similar visible absorption signatures are also observed for Ptdfppy (740 nm) and Ptppy (730 nm) (ESI, Fig. S8†). These absorption bands can be reasonably ascribed to [Pt^III^(C∧N)]^+^ because chemical oxidation of 50 μM Ptdfppy by 50 μM [Fe^III^(bpy)_3_]^3+^ produced a visible absorption band around 700 nm (*ε* = 3200 M^–1^ cm^–1^; ESI, Fig. S9†), although the irreversible oxidation of Ptppy and PtOMe hampered our ability to measure the visible absorption bands. As shown in Fig. 4a, the visible absorption spectrum of [Pt^III^(C∧N)]^+^ can be reproduced using quantum chemical simulation methods based on time-dependent density functional theory (CPCM(CH_3_CN)-TD-B3LYP/LANL2DZ:6-311+G(d,p)//B3LYP/LANL2DZ:6-311+G(d,p)). The molar absorbances (*ε*) of the one-electron oxidized species of PtOMe and Ptppy were thus estimated based on the simulated spectra. The decay trace of the one-electron oxidized species of PtOMe was monitored at 770 nm, and the rate constant for BeT (*k*_BeT_) was determined from the second-order linear fit of the decay traces to be 2.2 × 10^10^ M^–1^ s^–1^ (Fig. 4b; see ESI, Fig. S8† for other complexes). The *k*_BeT_ value was approximately one order of magnitude greater than the *k*_PeT_ values (Table 4), and was comparable to the diffusion rate constant (*k*_diff_) in CH_3_CN at 298 K, 1.93 × 10^10^ M^–1^ s^–1^, calculated according to the Stokes–Einstein–Smoluchowski equation, *k*_diff_ = (8*k*_B_ *N*_A_ *T*)/(3*η*), where *k*_B_, *N*_A_, *T*, and *η* are the Boltzmann constant, Avogadro's number, the absolute temperature, and the viscosity of the solvent, respectively. A significant fraction of the CF_3_I˙^–^ species returned to CF_3_I *via* BeT prior to escaping the solvent cage. This result revealed the importance of controlling the molecular structure to suppress BeT.

**Fig. 5:**
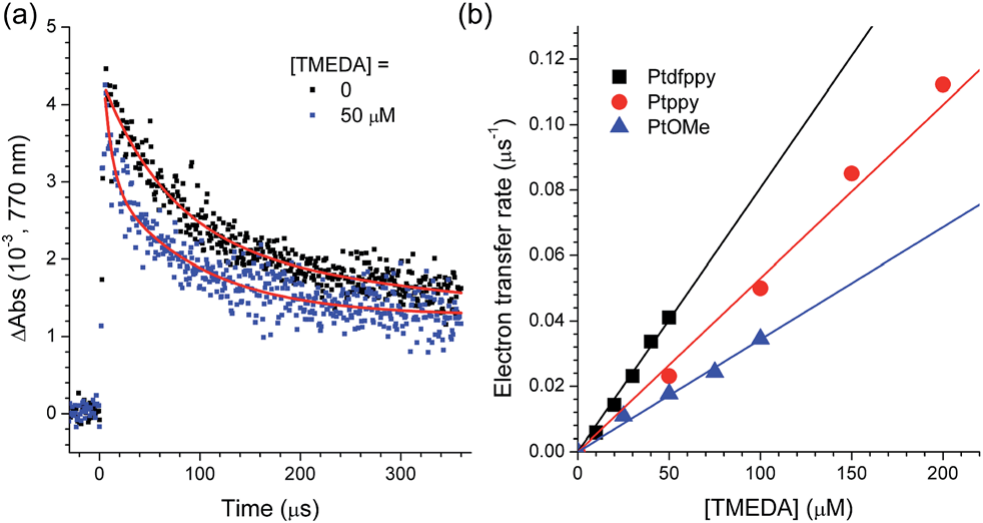
Determination of the rate constants for the regeneration (*k*_regen_) of the Pt(ii) complex catalysts. (a) Decay traces of the 770 nm absorption band of the one-electron oxidized PtOMe (100 μM in deaerated CH_3_CN, O.D. at 355 nm = 0.52) in the presence (50 μM) or absence of TMEDA after nanosecond pulsed photoexcitation at 355 nm. (b) Plots of the regeneration rate (1/*τ*(TMEDA) – 1/*τ*, where *τ*(TMEDA) and *τ* are the half-lives of the 770 nm traces in the presence and absence of TMEDA, respectively) *vs.* the concentration of TMEDA. The *k*_regen_ values were determined according to the single exponential curve fitting.

The photoredox cycle was completed by the one-electron reduction of [Pt^III^(C∧N)]^+^. Regeneration was accomplished primarily by electron transfer from a sacrificial electron donor, such as TMEDA, as determined based on the positive driving force (–Δ*G*_eT_ = *e*[*E*_ox_(TMEDA) – *E*_ox_(Pt)] = 0.05–0.15 eV). This process was monitored by acquiring the decay traces of [Pt^III^(C∧N)]^+^ under various concentrations of TMEDA. Fig. 5a shows the decay traces of the 770 nm absorption band for the deaerated acetonitrile solutions containing 100 μM PtOMe and 50 mM CF_3_I in the presence and absence of TMEDA. The half-life of the decay trace decreased from 70 μs to 25 μs upon the addition of 50 μM TMEDA. This behavior was ascribed to the reductive regeneration of PtOMe by TMEDA. The regeneration rate was calculated from the relationship, 1/*τ*(TMEDA) – 1/*τ*, where *τ*(TMEDA) and *τ* are the half-lives of the 770 nm decay traces in the presence and absence of TMEDA, respectively. The pseudo-first order linear fit of the regeneration rates *vs.* the concentration of TMEDA yielded the rate constants for regeneration (*k*_regen_) in the range of 3.4 to 7.7 × 10^8^ M^–1^ s^–1^. As shown in Table 4, these values are comparable to the *k*_PeT_ values but are approximately one order of magnitude smaller than *k*_BeT_.

The classical Marcus theory for adiabatic outer-sphere electron transfer provided a valuable basis for correlating the kinetic parameters (*i.e.*, *k*_PeT_, *k*_BeT_, and *k*_regen_) to the driving force. The electron transfer steps could be best described using eqn (1) with consideration for the diffusion process:^92,93^
1
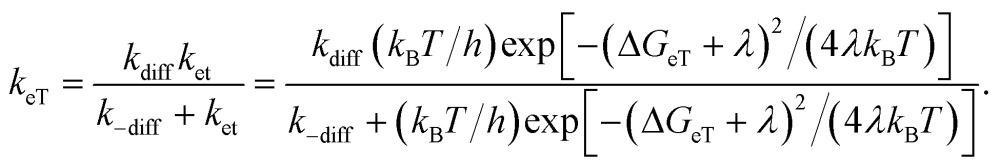
In eqn (1), *k*_et_, *h*, Δ*G*_eT_, and *λ* are the rate for adiabatic outer-sphere electron transfer, the Planck constant, the free energy change, and the reorganization energy for electron transfer, respectively.^94^

**Fig. 6:**
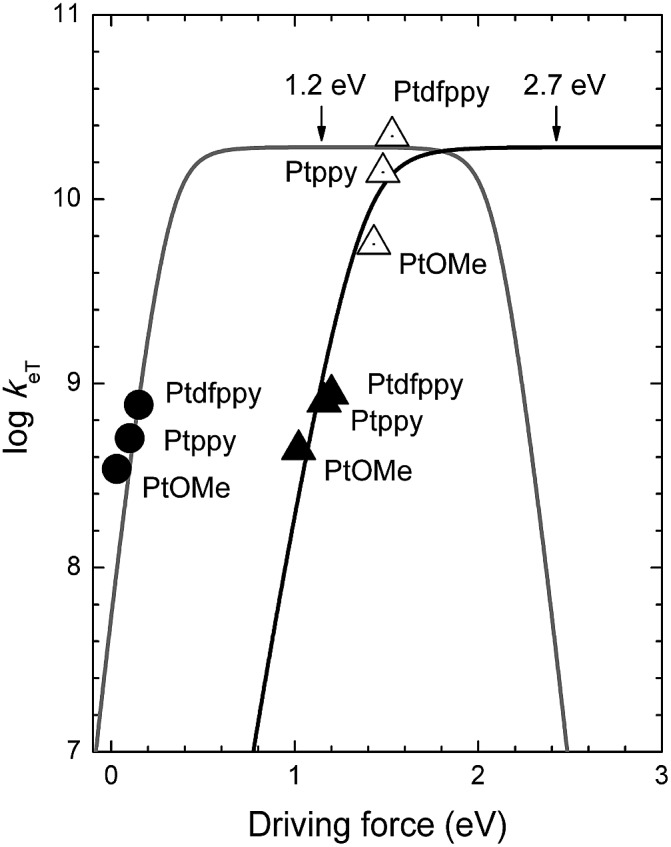
Plots of the log(rate constant) (log *k*_eT_) *vs.* the driving force for oxidative photoinduced electron transfer (*k*_PeT_, filled triangles), back electron transfer (*k*_BeT_, empty triangles), and regeneration of the Pt(ii) complex (*k*_regen_, filled circles) at 298 K. Curves show the theoretical plots of eqn (1) at *λ* = 1.2 eV (grey curve) and 2.7 eV (black curve).

As shown in Fig. 6, the *k*_PeT_ values adhere well to the theoretical curve calculated using the Marcus theory of electron transfer, with a *λ* value of 2.7 eV. The large reorganization energy indicates bond scission in CF_3_I after electron transfer. The positive dependence of *k*_PeT_ on –Δ*G*_PeT_ points to the occurrence of PeT in the Marcus normal region (*i.e.*, –Δ*G*_PeT_ < *λ*). The Marcus normal behavior suggests two possible strategies for accelerating PeT: (1) raising E*ox of the photoredox catalyst by incorporating ligands with wide band gap energies (*i.e.*, high Δ*E*_T_) and weak π-backbonding abilities (*i.e.*, cathodically shifting *E*_ox_), and (2) using ˙CF_3_ sources with large (more positive) *E*_red_ values, such as the Shibata reagent.^66^ The former approach involves a tradeoff because the effect may be offset by a reduction in the visible absorption cross-section.

The analyses using eqn (1) revealed a positive dependence of *k*_BeT_ on –Δ*G*_BeT_, with a *λ* value of 2.7 eV. This *λ* value was identical to that obtained under PeT, as expected for forward and reverse electron transfer. One potential strategy for retarding the hazardous BeT involves raising the *E*_ox_ of the photoredox catalysts using ligands with weak π-accepting or strong σ-donating properties. Alternatively, the use of ˙CF_3_ sources with large (more positive) *E*_red_ values may be advantageous.

The reductive regeneration by TMEDA was found to be essential because the trifluoromethylated products could not be obtained if TMEDA was replaced with a Brønsted base, such as K_3_PO_4_, K_2_HPO_4_ or KO^*t*^Bu (ESI, Tables S1 and S2†). As shown in Fig. 6, the regeneration by TMEDA followed a typical Rehm–Weller behavior with a *λ* value of 1.2 eV. The large positive dependence indicated that the rate of regeneration increased using catalysts with large *E*_ox_ values; however, predicting the influence of *k*_regen_ on the overall cycle was not straightforward due to the presence of an additional regeneration path involving radical species generated on the trifluoromethylated substrate (path C in Scheme 2). The above analyses based on the Marcus theory of electron transfer established that the photoredox catalysis performance could be improved by implementing the following molecular controls: (1) *E*_ox_ of the catalyst should be as small as possible to speed up PeT and to slow down BeT; (2) *E*_ox_ of the catalyst should be more positive than *E*_ox_ of the sacrificial electron donor to warrant regeneration; (3) the value of Δ*E*_T_ for the catalyst may be optimized, since larger values of Δ*E*_T_ will accelerate oxidative PeT but very large Δ*E*_T_ values eventually initiate competing reductive quenching.

Having established the photoredox catalytic cycle, we sought to understand the influence of the C∧N ligand structures on the overall catalytic performance. The quantum yields of the three Pt(ii) complexes for trifluoromethylation (QY) of 1-dodecene and *N*-methylpyrrole were determined using standard ferrioxalate actinometry (6.0 mM K_3_[Fe(C_2_O_4_)_3_], quantum yield = 1.1 at 420 nm; see the ESI† for experimental details). As summarized in Table 5, the QY values exceeded 100% in all cases. These results strongly indicated the significant involvement of radical propagation (path D in Scheme 2). This hypothesis was supported by the observation of a dark-state reaction after shutting off photoirradiation (ESI, Fig. S10†). It is noted that the Pt(ii) catalyst prepared with an electron-poor C∧N ligand produced a larger value of QY for the alkene, whereas the QY values for the heteroarene exhibited an opposite trend. Because the radical propagation and subsequent steps, including iodination and E2 elimination, were independent of the identity of the photoredox catalyst, variations in QY could be explained in terms of the photoredox cycle. Obviously, the QYs for 1-dodecene followed a trend opposite to those of *f* and *k*_BeT_ for the series of Pt(ii) complexes, suggesting that the effects of *f* and *k*_BeT_ would be marginal. One possible explanation for the QY trend is that regeneration is the limiting step in the overall catalytic performance. Of note, in Table 5 are the greater QY values of the Pt(ii) complexes over those of the well-established Ir(iii) and Ru(ii) catalysts. The improved photon economy of the Pt(ii) catalysts is likely due to elimination of the hazardous reductive quenching pathway that exists in the Ir(iii) and Ru(ii) catalyst systems.

**Table 5:** Quantum yields for trifluoromethylation of 1-dodecene and *N*-methylpyrrole by the Pt(ii) complexes and the established photoredox catalysts based on Ir(iii) and Ru(ii) complexes

	Ptdfppy	Ptppy	PtOMe	*fac*-Ir(ppy)_3_	[Ir(ppy)_2_(dtbbpy)]PF_6_	[Ru(bpy)_3_]Cl_2_
QY_alkene_ ^*a*^	18	17	16	12	10	6.5
QY_hetero_ ^*b*^	2.4	3.9	4.8	3.0	2.5	0.67

^*a*^Quantum yields for trifluoromethylation of 1-dodecene.

^*b*^Quantum yields for trifluoromethylation of *N*-methylpyrrole. The quantum yields were determined using standard ferrioxalate actinometry (6.0 mM K_3_[Fe(C_2_O_4_)_3_], QY = 1.1 at 420 nm (light intensity = 6.7 × 10^–10^ Einstein s^-1^)).

## Conclusions

We demonstrated the photoredox catalytic properties of a series of cyclometalated Pt(ii) complexes for use in the trifluoromethylation of non-prefunctionalized alkenes and heteroarenes under visible light irradiation. The Pt(ii) complexes displayed excellent catalytic performances, as demonstrated by high yields and functional group tolerance. The oxidative quenching pathway was exclusively allowed in the photoredox catalysis cycle due to the high excited-state redox potentials (E*ox and E*red) of the Pt(ii) catalysts. Direct spectroscopic measurements revealed Marcus normal behaviors for the photoinduced electron transfer, back electron transfer, and reductive regeneration processes. These results suggested several molecular strategies that could be used to enhance the catalyst performances. Forward photoinduced electron transfer to generate ˙CF_3_ could be accelerated by incorporating ligands with a high triplet-state energy and weak π-accepting properties, whereas hazardous back electron transfer could be minimized through the use of ligands having weak π-accepting or strong σ-donating properties. Alternatively, ˙CF_3_ reagents with low (*i.e.*, more positive) reduction potentials, such as the Shibata reagent, could have identical effects. Correlations between the quantum yields and the rate constants for electron transfer pointed to the notion that regeneration by a sacrificial electron donor or radical species of the trifluoromethylated substrate could be a limiting process in the overall catalysis cycle. Evidence for radical propagation suggests an oxidation potential smaller than –0.91 V *vs.* SCE for the radical species of the trifluoromethylated substrate. Accordingly, a large driving force exceeding 1.43 eV for catalyst regeneration by the radical species of the trifluoromethylated substrate can be estimated, strongly supporting that regeneration by the sacrificial electron donor may be the limiting process. We hope that the research described in this work will provide useful insights into the future development of photoredox catalysts for a range of organic transformations.
