# Supplementary material for: Mechanisms and applications of cyclometalated Pt(ii) complexes in photoredox catalytic trifluoromethylation
Source: Chem Sci. 2014 Nov 24;6(2):1454–64. doi: 10.1039/c4sc02537g (PMC5811153; doi:10.1039/c4sc02537g)
Supplement: Supplementary file 1 [file SC-006-C4SC02537G-s001.pdf]

Electronic Supplementary Material (ESI) for Chemical Science.
This journal is © The Royal Society of Chemistry 2014

Electronic Supplementary Information for

Mechanisms and Applications of Cyclometalated Pt(II) Complexes in Photoredox Catalytic Trifluoromethylation

Won Joon Choi,[a] Sungkyu Choi,[b] Kei Ohkubo,[c] Shunichi Fukuzumi,[c]* Eun Jin Cho,[b]* and Youngmin You[a]*

[a]Department of Advanced Materials Engineering for Information and Electronics, Kyung Hee University, Yongin, Gyeonggi-do 446-701, Korea,
[b]Department of Applied Chemistry & Department of Bionanotechnology, Hanyang University, Ansan, Gyeonggi-do 426-791, Korea,
[c]Department of Material and Life Science, Graduate School of Engineering, Osaka University, ALCA, Japan Science and Technology Agency (JST), Suita, Osaka 565-0871, Japan.

CONTENTS

Experimental details S3

Scheme S1. Synthesis of the Pt(II) complexes S4

Fig. S1. Spectroscopic data for the Pt(II) complexes S14

Fig. S2. Cyclic and differential pulse voltammograms of the Pt(II) complexes S14

Fig. S3. Differential pulse voltammogram of CF_3I S15

Fig. S4. Transient absorption spectra of (a) 1.0 mM Ptdfppy, (b) 1.0 mM Ptppy, and (c) 1.0 mM PtOMe (N_2-saturated acetonitrile) upon femtosecond laser excitation at 400 nm S15

Fig. S5. Phosphorescence decay traces of (a) 50 μM Ptdfppy (λ_{obs} = 465 nm) and (b) 50 μM Ptppy (λ_{obs} = 483 nm) (deaerated CH_3CN) with increasing the concentration of CF_3I (0–20 mM) (λ_{ex} = 377 nm, temporal resolution = 16 ns) S16

Fig. S6. ESR spectrum of a deaerated acetonitrile solution (78 K) of 1.0 mM Ptppy and 10 mM CF_3I upon photoexcitation at λ > 300 nm S16

Fig. S7. Photoluminescence decay traces of deaerated acetonitrile solutions containing 50 μM Pt(II) complex in the absence and presence of 20 mM TMEDA S16

Fig. S8. Nanosecond laser flash photolysis (λ_{ex} = 355 nm) for deaerated acetonitrile solutions containing (a and b) 100 μM Ptdfppy (O.D. at 355 nm = 0.67) and 50 mM CF_3I and (c and d) 100 μM Ptppy (O.D. at 355 nm = 0.62) and 50 mM CF_3I S17

Fig. S9. UV−vis−NIR absorption spectra of 50 μM Ptdfppy (acetonitrile) before (black) and after (red) the addition of 50 μM $[Fe(bpy)_3]^{3+}$ S17

Fig. S10. Progress of photoredox catalytic trifluoromethylation of 1-dodecene during the alternating cycle of photoirradiation (420 nm blue LED (7 W) for 30 min) and a dark reaction (for 30 min) S18

Fig. S11. ^{1}H NMR spectrum (400 MHz, $CDCl_3$) of Ptdfppy S18

Fig. S12. ^{13}C{^{1}H} NMR spectrum (100 MHz, $CDCl_3$) of Ptdfppy S19

Fig. S13. ^{19}F NMR spectrum (376 MHz, $CDCl_3$) of Ptdfppy S19

Fig. S14. ^{1}H NMR spectrum (400 MHz, $CDCl_3$) of Ptppy S20

Fig. S15. ^{13}C{^{1}H} NMR spectrum (100 MHz, $CDCl_3$) of Ptppy S20

Fig. S16. ^{1}H NMR spectrum (400 MHz, $CDCl_3$) of PtOMe S21

Fig. S17. ^{13}C{^{1}H} NMR spectrum (100 MHz, $CDCl_3$) of PtOMe S21

Fig. S18. ^{1}H NMR spectrum (400 MHz, $CDCl_3$) of **2a** S22

Fig. S19. ^{13}C NMR spectrum (101 MHz, $CDCl_3$) of **2a** S22

Fig. S20. ^{19}F NMR spectrum (377 MHz, $CDCl_3$) of **2a** S23

Fig. S21. ^{1}H NMR spectrum (400 MHz, $CDCl_3$) of **2b** S23

Fig. S22. ^{13}C NMR spectrum (101 MHz, $CDCl_3$) of **2b** S24

Fig. S23. ^{19}F NMR spectrum (377 MHz, $CDCl_3$) of **2b** S24

Fig. S24. ^{1}H NMR spectrum (400 MHz, $CDCl_3$) of **2c** S25

Fig. S25. ^{19}F NMR spectrum (377 MHz, $CDCl_3$) of **2c** S25

Fig. S26. ^{1}H NMR spectrum (400 MHz, $CDCl_3$) of **2d** S26

Fig. S27. ^{13}C NMR spectrum (101 MHz, $CDCl_3$) of **2d** S26

Fig. S28. ^{19}F NMR spectrum (377 MHz, $CDCl_3$) of **2d** S27

Fig. S29. ^{1}H NMR spectrum (400 MHz, $CDCl_3$) of **2e** S27

Fig. S30. ^{13}C NMR spectrum (101 MHz, $CDCl_3$) of **2e** S28

Fig. S31. ^{19}F NMR spectrum (377 MHz, $CDCl_3$) of **2e** S28

Fig. S32. ^{1}H NMR spectrum (400 MHz, $CDCl_3$) of **2f** S29

Fig. S33. ^{13}C NMR spectrum (101 MHz, $CDCl_3$) of **2f** S29

Fig. S34. ^{19}F NMR spectrum (377 MHz, $CDCl_3$) of **2f** S30

Fig. S35. ^{1}H NMR spectrum (400 MHz, $CDCl_3$) of **2g** S30

Fig. S36. ^{13}C NMR spectrum (101 MHz, $CDCl_3$) of **2g** S31
Fig. S37. ^{19}F NMR spectrum (377 MHz, $CDCl_3$) of **2g** S31
Fig. S38. ^{1}H NMR spectrum (400 MHz, $CDCl_3$) of **2h** S32
Fig. S39. ^{13}C NMR spectrum (101 MHz, $CDCl_3$) of **2h** S32
Fig. S40. ^{19}F NMR spectrum (377 MHz, $CDCl_3$) of **2h** S33
Fig. S41. ^{1}H NMR spectrum (400 MHz, $CDCl_3$) of **2i** S33
Fig. S42. ^{13}C NMR spectrum (101 MHz, $CDCl_3$) of **2i** S34
Fig. S43. ^{19}F NMR spectrum (377 MHz, $CDCl_3$) of **2i** S34
Fig. S44. ^{1}H NMR spectrum (400 MHz, $CDCl_3$) of **2j** S35
Fig. S45. ^{13}C NMR spectrum (101 MHz, $CDCl_3$) of **2j** S35
Fig. S46. ^{19}F NMR spectrum (377 MHz, $CDCl_3$) of **2j** S36
Fig. S47. ^{1}H NMR spectrum (400 MHz, $CDCl_3$) of **4b** S36
Fig. S48. ^{13}C NMR spectrum (101 MHz, $CDCl_3$) of **4b** S37
Fig. S49. ^{19}F NMR spectrum (377 MHz, $CDCl_3$) of **4b** S37
Fig. S50. ^{1}H NMR spectrum (400 MHz, $CDCl_3$) of **4c** S38
Fig. S51. ^{13}C NMR spectrum (101 MHz, $CDCl_3$) of **4c** S38
Fig. S52. ^{19}F NMR spectrum (377 MHz, $CDCl_3$) of **4c** S39
Fig. S53. ^{1}H NMR spectrum (400 MHz, $CDCl_3$) of **4e** S39
Fig. S54. ^{13}C NMR spectrum (101 MHz, $CDCl_3$) of **4e** S40
Fig. S55. ^{19}F NMR spectrum (377 MHz, $CDCl_3$) of **4e** S40
Table S1. Optimization of the reaction conditions for trifluoromethylation of 1-dodecene S41
Table S2. Optimization of the reaction conditions for trifluoromethylation of 3-methylindole S42
Table S3. Optimization of the reaction conditions for trifluoromethylation of *N*-methylpyrrole S43
Table S4. Report of the optimized geometry for the one-electron oxidized species of Ptdfppy S44
Table S5. Report of the optimized geometry for the one-electron oxidized species of Ptppy S44
Table S6. Report of the optimized geometry for the one-electron oxidized species of PtOMe S45
References S45

Experimental Details

Materials.

The syntheses of the cyclometalated Pt(II) complexes were performed according to the method reported previously (Scheme S1).[1] The preparation of 2-(2,4-difluorophenyl)pyridine and 2-(3-methoxyphenyl)pyridine was reported earlier.[2] 6.0 mM ferrioxalate standard solution was prepared according to the protocol described in the literature.[3] Commercially available chemicals were used without further purification unless otherwise stated. All glassware and

magnetic stirring bars were thoroughly dried in a convection oven. THF was purified by filtering over anhydrous alumina columns prior to use. Reactions were monitored using thin layer chromatography (TLC). Commercial TLC plates (silica gel 60 F_{254}, Merck Co.) were developed and the spots were visualized under UV illumination at 254 or 365 nm. Silica gel column chromatography was performed using silica gel 60 (particle size 0.063–0.200 mm, Merck Co.). ^{1}H, ^{13}C, and ^{19}F NMR spectra referenced to deuterated solvents were collected with a Bruker Ultrashield 400 plus NMR spectrometer. FT–IR spectra were recorded on a Bruker Alpha FT–IR spectrometer for KBr pellets. Electrospray ionization mass spectra were obtained by using a Thermo Electrons Co. Finnigan LCQ Advantage Max spectrometer. High resolution mass spectral data were obtained from the Korea Basic Science Institute (Daegu) on a Jeol JMS 700 high resolution mass spectrometer. Elemental analysis was performed using an EA1110 or EA1112 (CE Instrument, Italy) for C, H, and N.

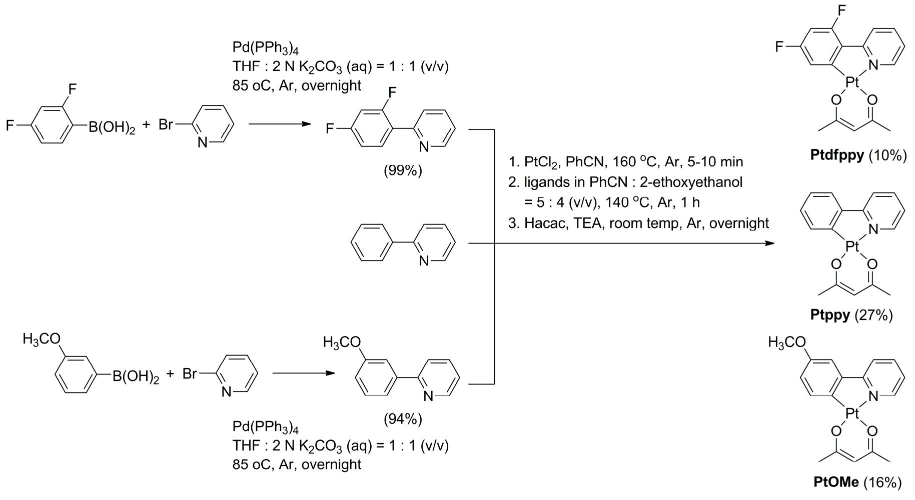

Scheme S1. Synthesis of the Pt(II) complexes.

Ptdfppy. 700 mg $PtCl_2$ (2.63 mmol) was added to a thoroughly dried two-necked round bottom flask. 6.0 mL anhydrous benzonitrile was delivered to a flask via a syringe under an Ar atmosphere. The reaction mixture was heated at 160 °C for 5 min, and the temperature was lowered to 140 °C. A mixture of 15 mL benzonitrile and 12 mL 2-ethoxyethanol containing 504 mg 2-(2,4-difluorophenyl)pyridine (2.63 mmol) was slowly added to the reaction mixture, after which the solution was heated for additional 1 h. After cooling down to room temperature, 1.5 mL 2-acetylacetone (Hacac, 14.5 mmol) and 1.9 mL dry TEA (14.0 mmol) were delivered using a syringe. The reaction mixture was stirred overnight under dark. The solution was poured onto

150 mL EtOAc and the organic layer was thoroughly washed with water (200 mL × five times). Recovered organic layer was dried over anhydrous $MgSO_4$, filtered and concentrated under reduced pressure. Residual benzonitrile was not completely removed. The crude product was subjected to silica gel column chromatography (eluent = CH_2Cl_2), and collected portions were concentrated under vacuum. Finally, repetitive reprecipitation in cold hexane furnished the desired product (yellow powder, 10%, 131 mg). ^{1}H NMR (400 MHz, CD_2Cl_2) δ 2.01 (s, 6H), 5.52 (s, 1H), 6.59 (m, 1H), 7.03 (dd, J = 19.8, 2.4 Hz, 1H), 7.17 (m, 1H), 7.87 (t, J = 7.7 Hz, 1H), 7.97 (m, 1H), 9.00 (d, J = 5.8 Hz, 1H). ^{13}C NMR (100 MHz, $CDCl_3$) δ 27.28, 28.35, 99.26, 99.52, 99.79, 102.93, 112.79 (d, J = 18 Hz), 121.30, 122.32 (d, J = 18 Hz), 138.91, 143.40, 147.51, 161.44, 165.40, 184.70, 186.26. ^{19}F NMR (376 MHz, $CDCl_3$) δ −112.30 (m, 1F), −106.80 (m, 1F). MS (ESI, positive): Calcd for $C_{16}H_{15}F_2NO_2Pt$ ($[M+H]^+$), 485; found, 485. Anal. Calcd. for $C_{16}H_{13}F_2NO_2Pt$: C, 39.68; H, 2.71; N, 2.89. Found: C, 39.80; H, 2.51; N, 2.80.

Ptppy. The identical procedure for Ptdfppy was employed for the synthesis of Ptppy (yellow powder, 318 mg, 27%), except the use of 2-phenylpyridine. ^{1}H NMR (400 MHz, $CDCl_3$) δ 2.00 (s, 6H), 5.55 (s, 1H), 7.09 (td, J = 7.5, 1.4 Hz, 1H), 7.15 (m, 2H), 7.46 (dd, J = 7.5, 1.3 Hz, 1H), 7.53 (d, J = 7.9 Hz, 1H), 7.65 (d, J = 7.8 Hz, 1H), 7.84 (td, J = 7.8, 1.6 Hz, 1H), 8.99 (d, J = 7.6 Hz, 1H). ^{13}C NMR (100 MHz, $CDCl_3$) δ 27.35, 28.50, 102.73, 118.55, 121.40, 123.22, 123.80, 129.50, 130.80, 138.34, 139.15, 144.85, 147.54, 168.58, 184.37, 186.00. MS (ESI, positive): Calcd for $C_{16}H_{16}NO_2Pt$ ($[M+H]^+$), 449; found, 449. Anal. Calcd. for $C_{16}H_{15}NO_2Pt$: C, 42.86; H, 3.37; N, 3.12. Found: C, 42.84; H, 3.51; N, 3.07.

PtOMe. The identical procedure for Ptdfppy was employed for the synthesis of PtOMe (yellow powder, 200 mg, 16%), except the use of 2-(3-methoxyphenyl)pyridine. ^{1}H NMR (400 MHz, $CDCl_3$) δ 1.98 (s, 6H), 3.82 (s, 3H), 5.49 (s, 1H), 6.86 (dd, J = 8.4, 2.7 Hz, 1H), 7.04 (d, J = 2.6 Hz, 1H), 7.13 (m, 1H), 7.40 (d, J = 8.4 Hz, 1H), 7.61 (d, J = 7.9 Hz, 1H), 7.83 (td, J = 7.8, 1.6 Hz, 1H), 8.97 (d, J = 5.2 Hz, 1H). ^{13}C NMR (100 MHz, $CDCl_3$) δ 27.32, 28.52, 55.73, 102.72, 109.11, 115.82, 118.59, 121.58, 129.11, 131.37, 138.31, 145.27, 147.61, 157.34, 168.34, 184.22, 185.87. MS (ESI, positive): Calcd for $C_{17}H_{18}NO_3Pt$ ($[M+H]^+$), 479; found, 479. Anal. Calcd. for $C_{17}H_{17}NO_3Pt$: C, 42.68; H, 3.58; N, 2.93. Found: C, 42.74; H, 3.72; N, 3.00.

Trifluoromethylation of alkenes

An oven-dried resealable test tube equipped with a magnetic stir bar was charged with an alkene (0.50 mmol), sealed with a silicone septa screw-cap, and degassed by alternating vacuum and argon backfill cycles. A solution of Ptppy (1.0 mol %, 5.0 μmol) in CH_3CN (2.0 mL, 0.25 M) and DBU (1.0 mmol) were then added to the tube under argon. CF_3I (1.5 mmol) was then delivered into the reaction mixture using a gastight syringe. The test tube was placed under blue

LEDs (7 W) at room temperature for 5−10 h, and the progress of the reaction was monitored by TLC or gas chromatography. The reaction mixture was diluted in diethyl ether, and washed with water and brine. The organic layers were dried over $MgSO_4$, concentrated in vacuo, and purified by flash column chromatography on silica gel to give the trifluoromethylated alkene.

Analytic Data for Alkene Substrates

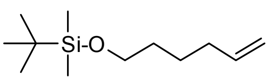

1d: Colorless oil; ^{1}H NMR (400 MHz, $CDCl_3$) δ 5.81 (ddt, J = 16.8, 10.0, 7.0 Hz, 1H), 5.00 (ddt, J = 17.0, 2.0, 1.6 Hz, 1H), 4.94 (ddt, J = 10.0, 2.0, 1.2 Hz, 1H), 3.61 (t, J = 6.4 Hz, 2H), 2.06 (tddd, J = 7.0, 7.0, 1.6, 1.2 Hz, 2H), 1.57−1.49 (m, 2H), 1.47−1.38 (m, 2H), 0.89 (s, 9H), 0.05 (s, 6H); ^{13}C NMR (101 MHz, $CDCl_3$) δ 139.16, 114.57, 63.29, 33.76, 32.52, 26.20, 25.38, 18.59, –5.06; FT–IR (neat): ν_{max} = 2930, 2859, 1472, 1255, 1103 cm^{-1}; R_f = 0.83 (only hexanes).

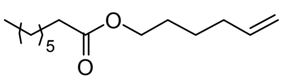

1e: Colorless oil; ^{1}H NMR (400 MHz, $CDCl_3$) δ 5.79 (ddt, J = 17.2, 10.0, 6.8 Hz, 1H), 5.01 (ddt, J = 17.2, 2.0, 1.6 Hz, 1H), 4.96 (ddt, J = 10.0, 2.0, 1.2 Hz, 1H), 4.07 (t, J = 6.8 Hz, 2H), 2.29 (t, J = 7.6 Hz, 2H), 2.08 (tddd, J = 6.8, 6.8, 1.6, 1.2 Hz, 2H), 1.68–1.58 (m, 2H), 1.50–1.41 (m, 2H), 1.35–1.25 (m, 10H), 0.88 (t, J = 6.8 Hz, 3H); ^{13}C NMR (101 MHz, $CDCl_3$) δ 173.93, 138.37, 114.87, 64.17, 34.44, 33.39, 31.78, 29.22, 29.04, 28.21, 25.32, 25.11, 22.70, 14.12; FT–IR (neat): ν_{max} = 2929, 2858, 1740, 1641, 1459, 1168 cm^{-1}; HRMS m/z (EI) calc. for $C_{14}H_{26}O_2$ [M$^+$] 226.1933, found 226.1936; R_f = 0.70 (hex/EtOAc, 8/1).

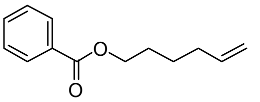

1f: Colorless oil; ^{1}H NMR (400 MHz, $CDCl_3$) δ 8.05 (dd, J = 8.4, 1.2 Hz, 2H), 7.55 (tt, J = 7.6, 1.2 Hz, 1H), 7.43 (dd, J = 8.4, 7.6 Hz, 2H), 5.82 (ddt, J = 17.0, 10.0, 6.8 Hz, 1H), 5.04 (ddt, J = 17.0, 2.0, 1.6 Hz, 1H), 4.98 (ddt, J = 10.0, 2.0, 1.2 Hz, 1H), 4.33 (t, J = 6.4 Hz, 2H), 2.13 (tddd, J = 6.8, 6.8, 1.6, 1.2 Hz, 2H), 1.83–1.75 (m, 2H), 1.60–1.51 (m, 2H); ^{13}C NMR (101 MHz, $CDCl_3$) δ 166.79, 138.50, 132.98, 130.64, 129.70, 128.49, 115.05, 65.04, 33.50, 28.34, 25.48; FT–IR (neat): ν_{max} = 2938, 2861, 1720, 1641, 1452, 1274 cm^{-1}; HRMS m/z (EI) calc. for $C_{13}H_{16}O_2$ [M$^+$] 204.1150, found 204.1153; R_f = 0.63 (hex/EtOAc, 8/1).

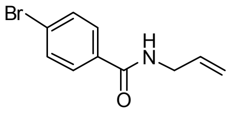

1g: White solid; ^{1}H NMR (400 MHz, $CDCl_3$) δ 7.67 (d, J = 8.6, 2H), 7.48 (d, J = 8.6 Hz, 2H), 7.39 (bs, 1H), 5.87 (dtd, J = 17.0, 10.2, 5.6 Hz, 1H), 5.19 (ddt, J = 17.0, 1.6, 1.2 Hz, 1H), 5.13 (ddt, J = 10.2, 1.6, 1.2 Hz, 1H), 4.02–3.96 (m, 2H); ^{13}C NMR (101 MHz, $CDCl_3$) δ 166.85, 133.97,

133.24, 131.64, 128.82, 126.11, 116.49, 42.54; FT–IR (neat): ν_{max} = 3295, 3074, 2248, 1633, 1538 cm^{-1}; HRMS *m/z* (EI) calc. for $C_{10}H_{10}BrNO$ [M^{+}] 238.9946, found 238.9946; R_f = 0.28 (hex/EtOAc, 2/1).

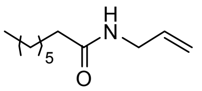

1h: Colorless oil; ^{1}H NMR (400 MHz, $CDCl_3$) δ 6.18 (bs, 1H), 5.77 (ddt, *J* = 17.2, 10.0, 5.6 Hz, 1H), 5.11 (ddt, *J* = 17.2, 1.6, 1.6 Hz, 1H), 5.05 (ddt, *J* = 10.0, 1.6, 1.2 Hz, 1H), 3.83–3.78 (m, 2H), 2.15 (t, *J* = 7.6 Hz, 2H), 1.58 (tt, *J* = 7.6, 7.6 Hz, 2H), 1.30–1.16 (m, 8H), 0.82 (t, *J* = 7.0 Hz, 3H); ^{13}C NMR (101 MHz, $CDCl_3$) δ 173.37, 134.54, 166.10, 41.92, 36.76, 31.78, 29.38, 29.12, 25.93, 22.68, 14.13; FT–IR (neat): ν_{max} = 3289, 2927, 1644, 1547 cm^{-1}; HRMS *m/z* (EI) calc. for $C_{11}H_{21}NO$ [M^{+}] 183.1623, found 183.1624; R_f = 0.30 (hex/EtOAc, 2/1).

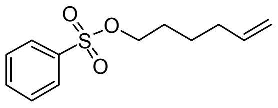

1j: Colorless oil; ^{1}H NMR (400 MHz, $CDCl_3$) δ 7.93–7.53 (m, 5H), 5.71 (ddt, *J* = 17.2, 10.4, 6.4 Hz, 1H), 4.99–4.91 (m, 2H), 4.05 (t, *J* = 6.4 Hz, 2H), 2.00 (tddd, *J* = 6.8, 6.8, 1.2, 1.2 Hz, 2H) 1.70–1.61 (m, 2H), 1.45–1.36 (m, 2H); ^{13}C NMR (101 MHz, $CDCl_3$) δ 137.94, 136.21, 133.83, 129.34, 127.89, 115.17, 70.80, 32.96, 28.24, 24.58; FT–IR (neat): ν_{max} = 2938, 1641, 1360, 1187, 935 cm^{-1}; HRMS *m/z* (FAB) calc. for $C_{12}H_{17}O_3S$ [M+H]$^{+}$ 241.0898, found 241.0895; R_f = 0.53 (hex/EtOAc, 4/1).

Analytic Data for Trifluoromethylated Alkenes

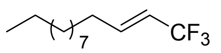

2a: Colorless oil; ^{1}H NMR (400 MHz, $CDCl_3$) δ 6.37 (dtq, *J* = 16.0, 6.8, 2.4 Hz, 1H), 5.60 (dqt, *J* = 16.0, 6.4, 1.6 Hz, 1H), 2.19–2.09 (m, 2H), 1.48–1.38 (m, 2H), 1.36–1.20 (m, 14H), 0.88 (t, *J* = 6.8 Hz, 3H); ^{13}C NMR (101 MHz, $CDCl_3$) δ 141.06 (q, *J* = 6.6 Hz), 123.38 (q, *J* = 270.1 Hz), 118.49 (q, *J* = 33.2 Hz), 32.15, 31.70, 29.83, 29.77, 29.61, 29.57, 29.28, 28.19, 22.93, 14.33; ^{19}F NMR (377 MHz, $CDCl_3$) δ –64.97; HRMS *m/z* (EI) calc. for $C_{13}H_{23}F_3$ [M^{+}] 236.1752, found 236.1750; R_f = 0.95 (only hexanes).

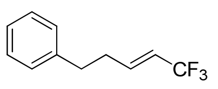

2b: Colorless oil; ^{1}H NMR (400 MHz, $CDCl_3$) δ 7.38–7.18 (m, 5H), 6.44 (dtq, *J* = 15.6, 6.8, 2.0 Hz, 1H), 5.66 (dqt, *J* = 15.6, 6.4, 1.2 Hz, 1H), 2.79 (t, *J* = 7.6 Hz, 2H), 2.54–2.45 (m, 2H); ^{13}C NMR (101 MHz, $CDCl_3$) δ 140.72, 139.86 (q, *J* = 6.6 Hz), 128.75, 128.56, 126.49, 123.21 (q, *J* = 270.2 Hz), 119.22 (q, *J* = 33.3 Hz), 34.54, 33.93; ^{19}F NMR (377 MHz, $CDCl_3$) δ –63.99; FT–IR (neat): ν_{max} = 2932, 1681, 1277, 1121 cm^{-1}; HRMS *m/z* (EI) calc. for $C_{11}H_{11}F_3$ [M^{+}] 200.0813, found 200.0814; R_f = 0.71 (only hexanes).

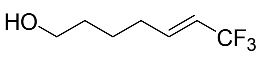

2c: Colorless oil; ^{1}H NMR (400 MHz, $CDCl_3$) δ 6.38 (dtq, J = 16.0, 6.8, 2.4 Hz, 1H), 5.63 (dqt, J = 16.0, 6.4, 1.6 Hz, 1H), 3.67 (t, J = 6.4 Hz, 2H), 2.25–2.15 (m, 2H), 1.65–1.49 (m, 4H), 1.25 (bs, 1H); ^{13}C NMR (101 MHz, $CDCl_3$) δ 140.51 (q, J = 6.6 Hz), 123.23 (q, J = 270.0 Hz), 118.78 (q, J = 33.2 Hz), 62.49, 32.05, 31.27, 24.35; ^{19}F NMR (377 MHz, $CDCl_3$) δ −64.90; FT–IR (neat): ν_{max} = 3385, 2940, 1681, 1275, 1120 cm^{-1}.

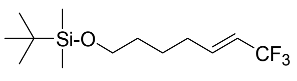

2d: Colorless oil; ^{1}H NMR (400 MHz, $CDCl_3$) δ 6.38 (dtq, J = 16.0, 6.8, 2.0 Hz, 1H), 5.63 (dqt, J = 16.0, 6.4, 1.6 Hz, 1H), 3.67 (t, J = 6.0 Hz, 2H), 2.24–2.14 (m, 2H), 1.58–1.46 (m, 4H), 0.89 (s, 9H), 0.05 (s, 6H); ^{13}C NMR (101 MHz, $CDCl_3$) δ 140.81 (q, J = 6.6 Hz), 123.32 (q, J = 270.1 Hz), 118.70 (q, J = 33.4 Hz), 62.92, 32.31, 31.42, 26.15, 24.56, 18.56, −5.11; ^{19}F NMR (377 MHz, $CDCl_3$) δ −64.93; FT–IR (neat): ν_{max} = 2932, 2859, 1681, 1257, 1123 cm^{-1}.

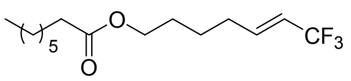

2e: Colorless oil; ^{1}H NMR (400 MHz, $CDCl_3$) δ 6.37 (dtq, J = 15.6, 6.8, 2.0 Hz, 1H), 5.62 (dqt, J = 15.6, 6.0, 1.6 Hz, 1H), 4.08 (t, J = 6.4 Hz, 2H), 2.31 (t, J = 7.2 Hz, 2H), 2.23–2.12 (m, 2H), 1.69–1.57 (m, 4H), 1.57–1.46 (m, 2H), 1.35–1.20 (m, 8H), 0.88 (t, J = 6.8 Hz, 3H); ^{13}C NMR (101 MHz, $CDCl_3$) δ 140.21 (q, J = 6.5 Hz), 123.18 (q, J = 270.0 Hz), 119.02 (q, J = 33.2 Hz), 63.91, 34.52, 31.86, 31.17, 29.31, 29.13, 28.24, 25.19, 24.62, 22.79, 14.24; ^{19}F NMR (377 MHz, $CDCl_3$) δ −63.41; FT–IR (neat): ν_{max} = 2932, 2859, 1737, 1273, 1121 cm^{-1}; R_f = 0.53 (hex/EtOAc, 8/1).

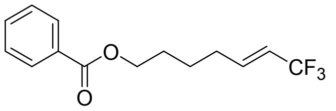

2f: Colorless oil; ^{1}H NMR (400 MHz, $CDCl_3$) δ 7.41–8.07 (m, 5H), 6.39 (dtq, J = 15.6, 6.8, 2.4 Hz, 1H), 5.64 (dqt, J = 15.6, 6.4, 1.6 Hz, 1H), 4.34 (t, J = 6.4, 2H), 2.29–2.18 (m, 2H), 1.84–1.76 (m, 2H), 1.67–1.57 (m, 2H); ^{13}C NMR (101 MHz, $CDCl_3$) δ 166.77, 140.21 (q, J = 6.7 Hz), 123.19 (q, J = 270.0 Hz), 119.05 (q, J = 33.4 Hz), 64.65, 31.20, 28.33, 24.72; ^{19}F NMR (377 MHz, $CDCl_3$) δ −64.74; FT–IR (neat): ν_{max} = 2948, 1720, 1275, 1117 cm^{-1}; HRMS *m/z* (EI) calc. for $C_{14}H_{15}F_3O_2$ [M^+] 272.1024, found 272.1026; R_f = 0.44 (hex/EtOAc, 8/1).

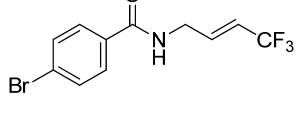

2g: White solid; ^{1}H NMR (400 MHz, $CDCl_3$) δ 7.67 (d, J = 8.8 Hz, 2H), 7.60 (d, J = 8.8 Hz, 2H), 6.47 (dtq, J = 16.0, 5.2, 2.4 Hz, 1H),

6.29 (bs, 1H), 5.81 (dqt, J = 16.0, 6.4, 2.0 Hz, 1H), 4.25–4.17 (m, 2H); ^{13}C NMR (101 MHz, $CDCl_3$) δ 166.74, 136.36 (q, J = 6.3 Hz), 132.72, 132.19, 128.78, 126.93, 122.89 (q, J = 270.5 Hz), 120.01 (q, J = 34.2 Hz), 40.24; ^{19}F NMR (377 MHz, $CDCl_3$) δ –64.17; FT–IR (neat): ν_{max} = 3316, 1651, 1540, 1301, 1110 cm^{-1}; HRMS m/z (EI) calc. for $C_{11}H_9BrF_3NO$ [M^+] 306.9820, found 306.9816; R_f = 0.36 (hex/EtOAc, 2/1).

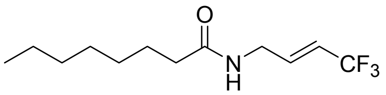

2h: Colorless oil; ^{1}H NMR (400 MHz, $CDCl_3$) δ 6.34 (dtq, J = 15.8, 5.2, 2.0 Hz, 1H), 6.14 (bs, 1H), 5.69 (dqt, J = 15.8, 6.4, 1.8 Hz, 1H), 3.98–3.91 (m, 2H), 2.20 (t, J = 7.6 Hz, 2H), 1.65–1.54 (m, 2H), 1.33–1.17 (m, 8H), 0.84 (t, J = 7.2 Hz, 3H); ^{13}C NMR (101 MHz, $CDCl_3$) δ 173.77, 136.90 (q, J = 6.3 Hz), 123.01 (q, J = 270.3 Hz), 119.12 (q, J = 34.0 Hz), 39.47, 36.61, 31.81, 29.40, 29.15, 25.88, 22.72, 14.14; ^{19}F NMR (377 MHz, $CDCl_3$) δ –64.28; FT–IR (neat): ν_{max} = 3289, 2930, 2859, 1651, 1548, 1303, 1124 cm^{-1}; HRMS m/z (EI) calc. for $C_{12}H_{20}F_3NO$ [M^+] 251.1497, found 251.1500; R_f = 0.38 (hex/EtOAc, 2/1).

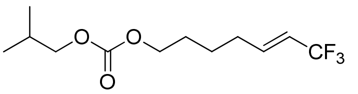

2i: Colorless oil; ^{1}H NMR (400 MHz, $CDCl_3$) δ 6.32 (dtq, J = 15.6, 6.8, 2.0 Hz, 1H), 5.58 (dqt, J = 15.6, 6.4, 1.6 Hz, 1H), 4.10 (t, J = 6.4 Hz, 2H), 3.87 (d, J = 6.8 Hz, 2H), 2.20–2.11 (m, 2H), 2.00–1.85 (m, 2H), 1.71–1.61 (m, 2H), 1.55–1.45 (m, 2H), 0.90 (d, J = 6.8 Hz, 6H); ^{13}C NMR (101 MHz, $CDCl_3$) δ 155.62, 140.09 (q, J = 6.6 Hz), 123.18 (q, J = 270.1 Hz), 119.13 (q, J = 33.4 Hz), 74.28, 67.55, 31.15, 28.70, 27.99, 24.42, 19.11; ^{19}F NMR (377 MHz, $CDCl_3$) δ –64.17; FT–IR (neat): ν_{max} = 2965, 1747, 1258, 1120 cm^{-1}; R_f = 0.53 (hex/EtOAc, 8/1).

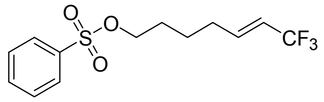

2j: Colorless oil; ^{1}H NMR (400 MHz, $CDCl_3$) δ 7.92–7.53 (m, 5H), 6.28 (dtq, J = 16.0, 6.8, 2.2 Hz, 1H), 5.56 (dqt, J = 16.0, 6.4, 1.6 Hz, 1H), 4.06 (t, J = 6.4 Hz, 2H), 2.16–2.06 (m, 2H), 1.71–1.62 (m, 2H), 1.52–1.43 (m, 2H); ^{13}C NMR (101 MHz, $CDCl_3$) δ 139.78 (q, J = 6.6 Hz), 136.21, 134.01, 129.47, 127.99, 123.08 (q, J = 270.1 Hz), 119.19 (q, J = 33.4 Hz), 70.40, 30.77, 28.32, 24.00; ^{19}F NMR (377 MHz, $CDCl_3$) δ –64.01; FT–IR (neat): ν_{max} = 2947, 1681, 1361, 1188, 1118 cm^{-1}; HRMS m/z (EI) calc. for $C_{13}H_{15}F_3O_3S$ [M^+] 308.0694, found 308.0698; R_f = 0.43 (hex/EtOAc, 4/1).

Trifluoromethylation of heteroarenes

An oven-dried resealable test tube equipped with a magnetic stir bar was charged with an acetonitrile solution (2.0 mL) containing heteroarene (0.50 mmol), TMEDA (1.0 mmol) and

Ptppy (2.0 mol %, 0.010 mmol), and sealed with a screw-cap. The reaction mixture was degassed by alternating vacuum and argon backfill cycles, after which CF_3I (1.5 mmol) was delivered by using a gastight syringe. The test tube was placed under blue LEDs at room temperature for 10–24 h, while progress was monitored by TLC or gas chromatography. The reaction mixture was diluted in diethyl ether and washed with water and brine. The organic layers were dried over $MgSO_4$, concentrated in vacuo, and purified by flash column chromatography on silica gel to give the trifluoromethylated heteroarene.

Analytic Data for Trifluoromethylated Heteroarenes

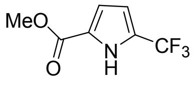

4b: White solid; ^{1}H NMR (400 MHz, $CDCl_3$) δ 10.61 (bs, 1H), 6.90–6.86 (m, 1H), 6.61–6.57 (m, 1H), 3.92 (s, 3H); ^{13}C NMR (101 MHz, $CDCl_3$) δ 161.80, 125.17 (q, J = 40.1 Hz), 125.15, 123.27 (q, J = 262.0 Hz), 115.19, 111.05 (q, J = 2.8 Hz), 52.37; ^{19}F NMR (377 MHz, $CDCl_3$) δ –60.41; FT–IR (neat): ν_{max} = 3264, 1707, 1286, 1107, 768 cm^{-1}; HRMS *m/z* (EI) calc. for $C_7H_6F_3NO_2$ [M^+] 193.0351, found 193.0351; R_f = 0.54 (hex/EtOAc, 4/1).

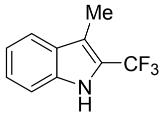

4c: Yellow solid; ^{1}H NMR (400 MHz, $CDCl_3$) δ 8.17 (bs, 1H), 7.65 (d, J = 7.8 Hz, 1H), 7.39 (d, J = 8.0 Hz, 1H), 7.33 (dd, J = 8.0, 7.8 Hz, 1H), 7.20 (dd, J = 8.0, 7.8 Hz, 1H), 2.45 (q, J = 1.8 Hz, 3H); ^{13}C NMR (101 MHz, $CDCl_3$) δ 135.78, 128.27, 124.98, 122.33 (q, J = 269.5 Hz), 121.74 (q, J = 36.6 Hz), 120.60, 120.31, 114.29 (q, J = 3.6 Hz), 111.76; ^{19}F NMR (377 MHz, $CDCl_3$) δ –58.61; FT–IR (neat): ν_{max} = 3393, 1452, 1161, 1114, 756 cm^{-1}; HRMS *m/z* (EI) calc. for $C_{10}H_8F_3N$ [M^+] 199.0609, found 199.0606; R_f = 0.47 (hex/EtOAc, 8/1).

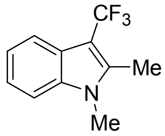

4e: Yellow solid; ^{1}H NMR (400 MHz, $CDCl_3$) δ 7.27 (d, J = 7.6 Hz, 1H), 7.30 (d, J = 8.0 Hz, 1H), 7.25 (dd, J = 8.0, 7.6 Hz, 1H), 7.19 (dd, J = 8.0, 7.6 Hz, 1H), 3.68 (s, 3H), 2.45 (q, J = 1.2 Hz, 3H); ^{13}C NMR (101 MHz, $CDCl_3$) δ 137.46 (q, J = 3.7 Hz), 136.34, 125.73 (q, J = 269.9 Hz), 124.61 (q, J = 1.4 Hz), 122.19, 121.24, 119.25, 109.41, 102.76 (q, J = 35.4 Hz), 29.67, 11.12; ^{19}F NMR (377 MHz, $CDCl_3$) δ –63.41; FT–IR (neat): ν_{max} = 2952, 1419, 1291, 1095 cm^{-1}; R_f = 0.38 (hex/EtOAc, 8/1).

Characterization.

Steady-state UV–vis absorption measurements. UV–vis absorption spectra were collected on a Varian Cary 50 spectrophotometer or an Agilent 8453 spectrophotometer at 298 K. 10 μM or 50 μM CH_3CN solutions of the Pt(II) complex were used for the measurements unless otherwise mentioned.

Steady-state photoluminescence measurements. Phosphorescence spectra were obtained using a Quanta Master 40 scanning spectrofluorimeter at room temperature. The 50 μM solutions were thoroughly deaerated by bubbling Ar for at least 30 min prior to performing the measurements. The photoluminescence quantum yields (PLQYs) were relatively determined according to following standard equation: $PLQY = PLQY_{ref} \times (I/I_{ref}) \times (A_{ref}/A) \times (n/n_{ref})^2$, where A, I, and n are the absorbance at the excitation wavelength, integrated photoluminescence intensity, and the refractive index of the solvent, respectively. Fluorescein as an aqueous 0.1 N NaOH solution was used as the external reference ($PLQY_{ref} = 0.79$). The refractive index of the 0.1 N NaOH solution was assumed to be identical to the value for pure water.

Photoluminescence lifetime measurements. Ar-saturated 50 μM solutions in CH_3CN were used for determination of the photoluminescence lifetimes of the Pt(II) complexes. Photoluminescence decay traces were acquired based on time-correlated single-photon-counting (TCSPC) techniques using a FluoTime 200 instrument (PicoQuant, Germany). A 377 nm diode laser (PicoQuant, Germany) was used as the excitation source. The photoluminescence signals were obtained using an automated motorized monochromator. Photoluminescence decay profiles were analyzed (OriginPro 8.0, OriginLab) using a single exponential decay model.

Nanosecond laser flash photolysis. An N_2-saturated CH_3CN sample solution in a 1 cm × 1 cm quartz cell was excited by a Nd:YAG laser (Continuum, SLII-10; 4–6 ns fwhm) at 355 nm with 5 mJ/pulse. Time courses of the transient absorption were measured by an InGaAs-PIN photodiode (Hamamatsu 2949) as a detector. The output from the photodiodes and a photomultiplier tube was recorded with a digitized oscilloscope (Tektronix, TDS3032; 300 MHz). All experiments were performed at 298 K.

Determination of quantum yields of trifluoromethylation (QY). The quantum yields for trifluoromethylation were determined by the standard ferrioxalate actinometry. A 0.0060 M $K_3[Fe(C_2O_4)_3]$ solution served as the chemical actinometer. 500 μL of the $K_3[Fe(C_2O_4)_3]$ solution was transferred to a 1 cm × 1 mm quartz cell, and the solution was photoirradiated with a monochromatized beam at 420 nm for 20 s. Then same amount of 1 % 1,10-phenanthroline in sodium acetate buffer solution (4.09 g CH_3COONa dissolved in 18 mL of 0.5 M H_2SO_4 and 32 mL of distilled water) were added and stored under dark for 1 h. The absorbance change at 510 nm was recorded. Inserting the value to eq 1 returned the light intensity value of 6.7×10^{-10} einstein s^{-1} at 420 nm:

$$\text{Light intensity } (I_0, \text{ einstein s}^{-1}) = (\Delta Abs(510 \text{ nm}) \times V)/(\Phi \times 11050 \text{ M}^{-1} \text{ cm}^{-1} \times \Delta t) \quad (1).$$

In eq 1, ΔAbs(510 nm), V, Φ, and Δt are the absorbance change at 510 nm, volume (L), the quantum yield (1.1) of the ferrioxalate actinometer at 420 nm,[3] and photoirradiation time (s), respectively. An CH_3CN solution containing 0.50 mmol 1-dodecene, 1.0 mol % Pt(II) catalyst, 2.0 mmol DBU, and 1.5 mmol CF_3I was photoirradiated under the identical condition for 30 min. The trifluoromethylated product was quantitated by ^{19}F NMR spectroscopy using 1,3,5-trifluorobenzene as an internal standard,[4] and inserted into eq 2:

$$QY_{CF_3} = \frac{[\text{product}] \times V}{I \times \Delta t} \qquad (2)$$

In eq 2, [product] is the molar concentration of the trifluoromethylated product determined by ^{19}F NMR spectroscopy, Δt (s) is the photoirradiated time, V is the volume of the solution (L), and I_0 is the light intensity obtained by eq 1 (einstein s^{-1}).

Electrochemical Characterization. Cyclic voltammetry and differential pulse voltammetry experiments were carried out using a CHI630 B instrument (CH Instruments, Inc.) using a three-electrode cell assembly. A Pt wire and a Pt microdisc were used as the counter and the working electrodes, respectively. A $Ag/AgNO_3$ couple was used as a pseudo reference electrode. Measurements were carried out in Ar-saturated CH_3CN (3 mL) using 0.10 M tetra-*n*-butylammonium hexafluorophosphate (Bu_4NPF_6) as the supporting electrolyte at scan rates of 100 mV s^{-1} (cyclic voltammetry) and 4.0 mV s^{-1} (differential pulse voltammetry). The concentration was 1.0 mM. A ferrocenium/ferrocene couple was employed as the external reference.

Photoinduced ESR measurements. A deaerated CH_3CN solution of 1.0 mM Ptppy and 10 mM CF_3I was delivered to an EPR cell (i.d. = 0.7 mm). EPR spectra were recorded on a JEOL JES-RE1XE spectrometer at 78 K under photoirradiation at $\lambda > 300$ nm. The magnitude of modulation was chosen to optimize the resolution and signal-to-noise (S/N) ratios of the observed spectra under non-saturating microwave power conditions. The g value was calibrated using a Mn^{2+} marker ($g = 2.034$, 1.981).

DFT/TD-DFT calculations. Quantum chemical calculations based on density functional theory (DFT) were carried out using a Gaussian 09 program.[5] Geometry optimization and single point calculations for the Pt complexes were performed using Becke's three parameter B3LYP exchange-correlation functional[6–8] and the "double-ξ" quality LANL2DZ basis set for the Pt atom, and the 6–311+G(d,p) basis set for all other atoms. A pseudo potential (LANL2DZ) was applied to replace the inner core electrons of the Pt atom, leaving the outer core $[(5s)^2(5p)^6]$

electrons and the $(5d)^6$ valence electrons. The polarizable continuum model (CPCM), parameterized for acetonitrile solvent, was applied during the geometry optimization step. Frequency calculations were subsequently performed to assess the stability of the convergence. Geometry optimization of the one-electron oxidized Pt complexes was carried out through the identical procedure, except the use of unrestricted B3LYP exchange-correlation functional. For TD−DFT calculations, the functional and the identical basis sets used for the geometry optimization were applied to the optimized geometries. The polarizable continuum model (CPCM) with a parameter set for acetonitrile was applied to account for solvation effects. Twenty lowest singlet and triplet states were calculated and analyzed. Simulation of the UV−vis absorption spectra were performed by employing a GaussSum program.[9] AOMix program was employed to estimate the MLCT character.[10]

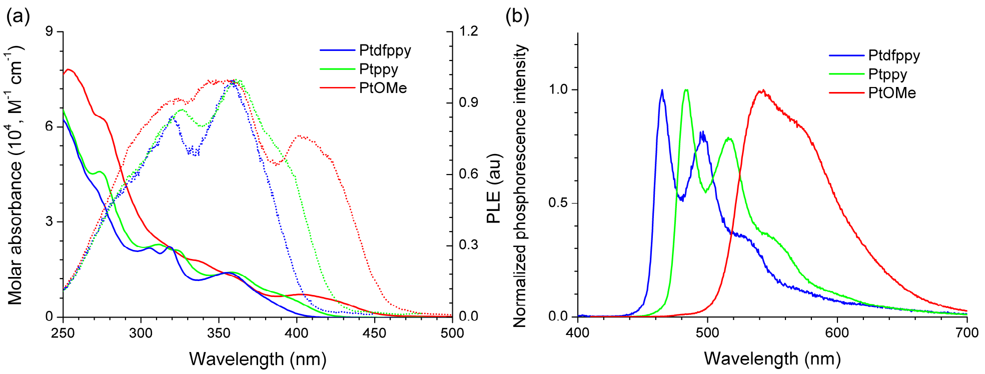

Fig. S1 Spectroscopic data for the Pt(II) complexes. (a) UV–vis absorption (solid lines) and photoluminescence excitation (dotted lines, λ_{obs} = 465 nm (Ptdfppy), 483 nm (Ptppy), and 540 nm (PtOMe)) spectra. (b) Phosphorescence spectra (λ_{ex} = 360 nm). Conditions: Ar-saturated CH_3CN solutions of 50 μM Pt complexes.

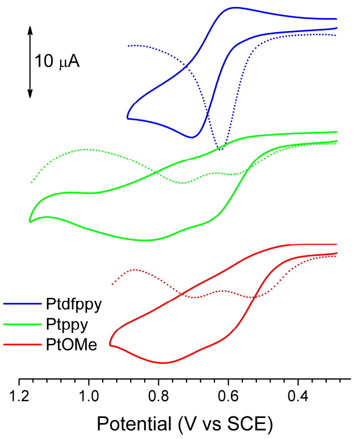

Fig. S2 Cyclic (solid lines) and differential pulse (dotted lines) voltammograms of the Pt(II) complexes. Conditions: 1.0 mM Pt(II) complex in deaerated CH_3CN containing 0.10 M $TBAPF_6$; a Pt microdisc and a Pt wire as the working and counter electrodes, respectively; $Ag/AgNO_3$ as the pseudo reference electrode; scan rates = 0.10 V s^{-1} (cyclic voltammetry) and 0.004 V s^{-1} (differential pulse voltammetry)..

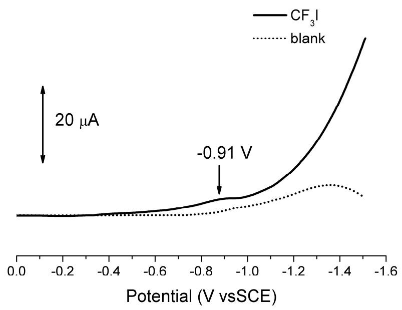

Fig. S3 Differential pulse voltammogram of CF_3I. Blank implies the electrolyte solution without CF_3I. Conditions: 10 mM CF_3I in deaerated CH_3CN containing 0.10 M $TBAPF_6$; a Pt microdisc and a Pt wire as the working and counter electrodes, respectively; $Ag/AgNO_3$ as the pseudo reference electrode; scan rate = 0.004 V s^{-1}.

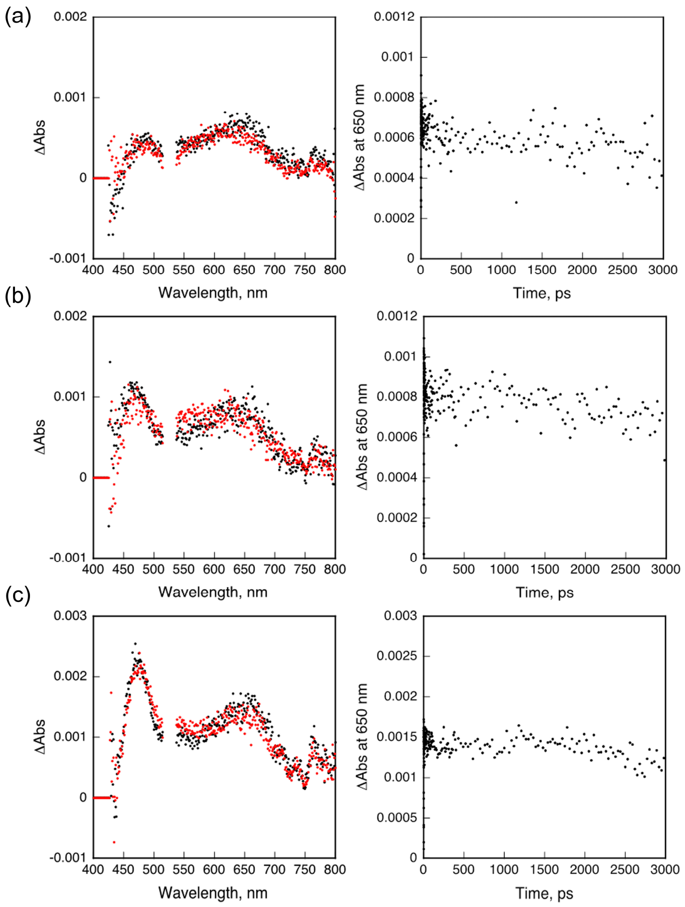

Fig. S4 Transient absorption spectra of (a) 1.0 mM Ptdfppy, (b) 1.0 mM Ptppy, and (c) 1.0 mM PtOMe (N_2-saturated acetonitrile) upon femtosecond laser excitation at 400 nm.

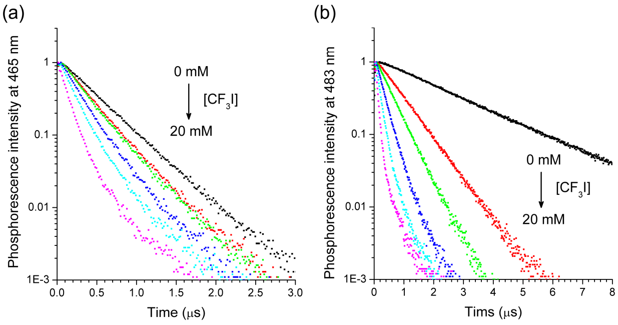

Fig. S5 Phosphorescence decay traces of (a) 50 μM Ptdfppy (λ_{obs} = 465 nm) and (b) 50 μM Ptppy (λ_{obs} = 483 nm) (deaerated CH_3CN) with increasing the concentration of CF_3I (0–20 mM) (λ_{ex} = 377 nm, temporal resolution = 16 ns). Electron transfer rates and their fits are shown in the main text, Figure 3.

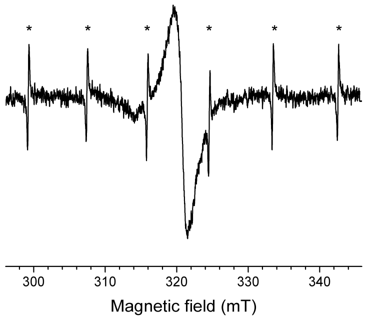

Fig. S6 ESR spectrum of a deaerated acetonitrile solution (78 K) of 1.0 mM Ptppy and 10 mM CF_3I upon photoexcitation at λ > 300 nm. The indicated marks (*) correspond to the signals from a Mn marker.

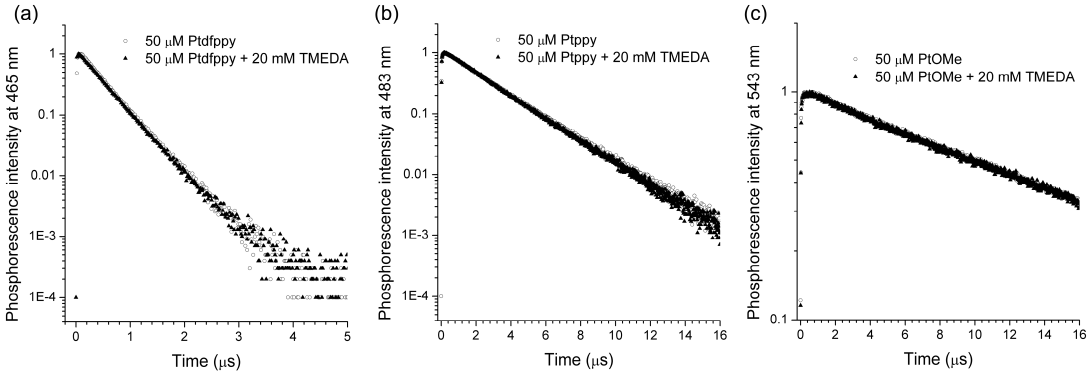

Fig. S7 Photoluminescence decay traces of deaerated acetonitrile solutions containing 50 μM Pt(II) complex in the absence and presence of 20 mM TMEDA: (a) Ptdfppy, (b) Ptppy, and (c) PtOMe.

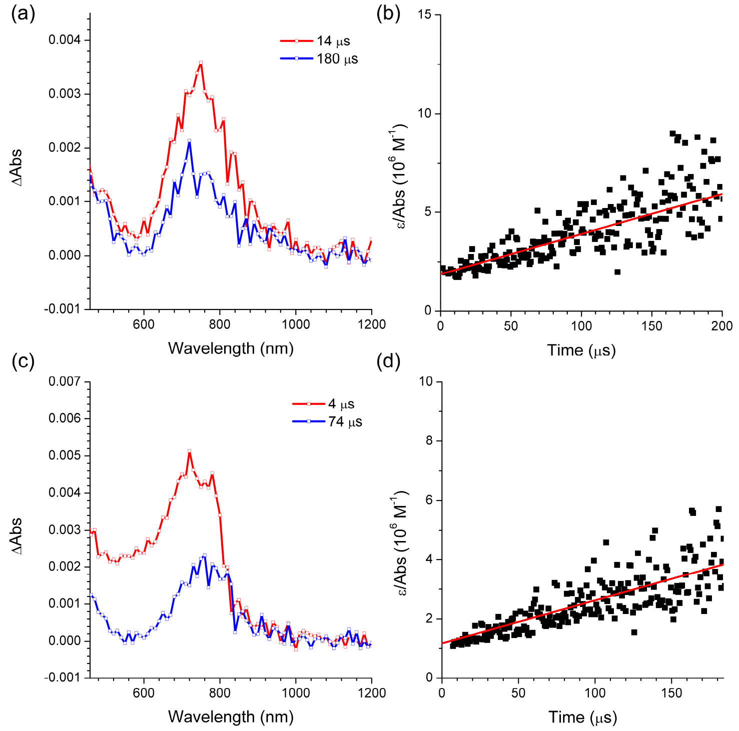

Fig. S8 Nanosecond laser flash photolysis (λ_{ex} = 355 nm) for deaerated acetonitrile solutions containing (a and b) 100 μM Ptdfppy (O.D. at 355 nm = 0.67) and 50 mM CF_3I, and (c and d) 100 μM Ptppy (O.D. at 355 nm = 0.62) and 50 mM CF_3I: Left, transient absorption spectra; right, determination of the rate constants for back electron transfer (k_{BeT}).

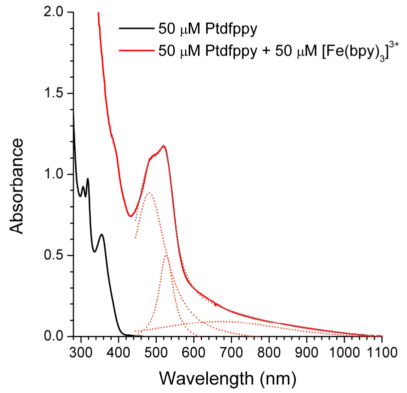

Fig. S9 UV–vis–NIR absorption spectra of 50 μM Ptdfppy (acetonitrile) before (black) and after (red) the addition of 50 μM $[Fe(bpy)_3]^{3+}$. Dotted lines are deconvoluted Gaussian curves (orange dotted lines) and their sum (grey dotted line).

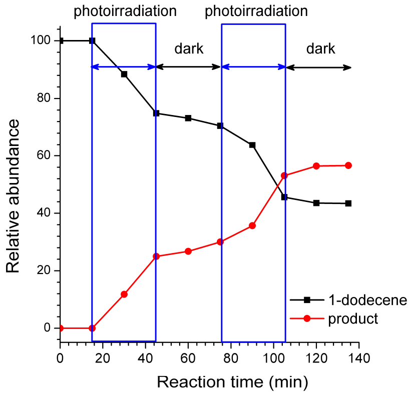

Fig. S10 Progress of photoredox catalytic trifluoromethylation of 1-dodecene during the alternating cycle of photoirradiation (420 nm blue LED (7 W) for 30 min) and a dark reaction (i.e., without light for 30 min). The relative abundance of 1-dodecene and its trifluromethylated product was quantitated by GC employing dodecane as an internal standard.

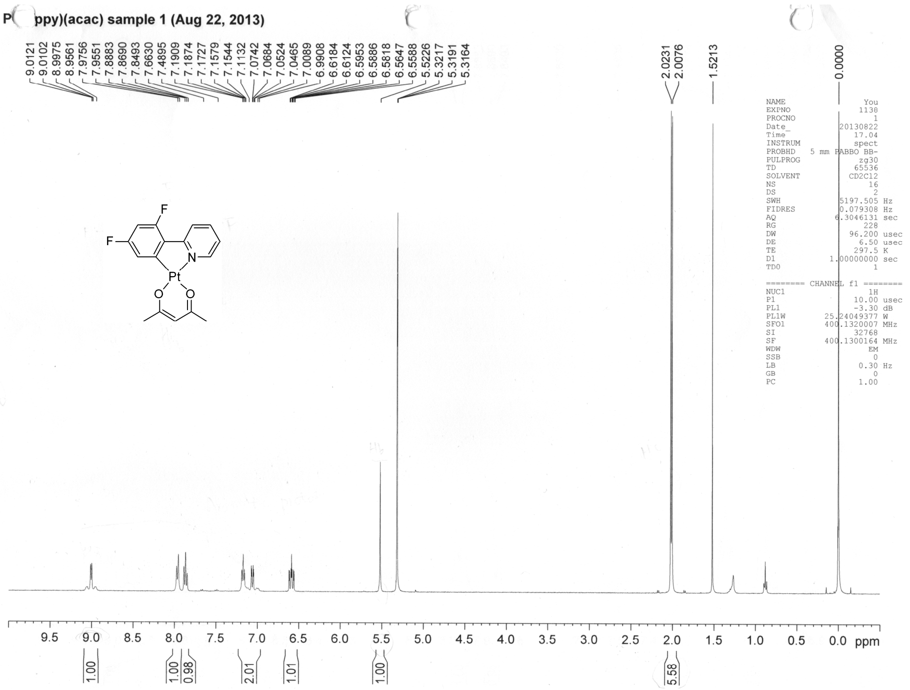

Fig. S11 ^{1}H NMR spectrum (400 MHz, $CDCl_3$) of Ptdfppy.

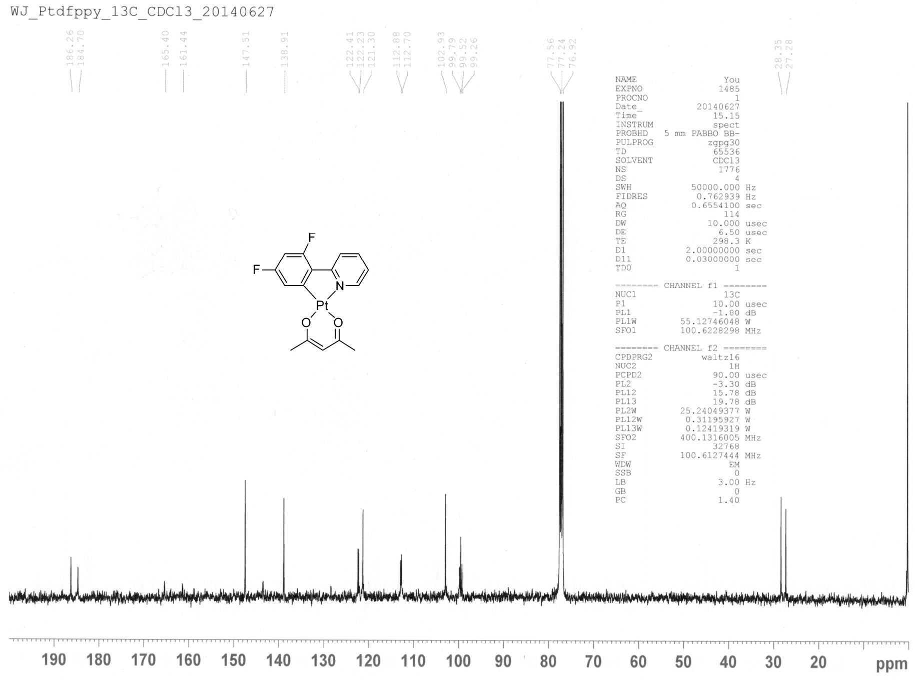

Fig. S12 $^{13}C\{^1H\}$ NMR spectrum (100 MHz, $CDCl_3$) of Ptdfppy.

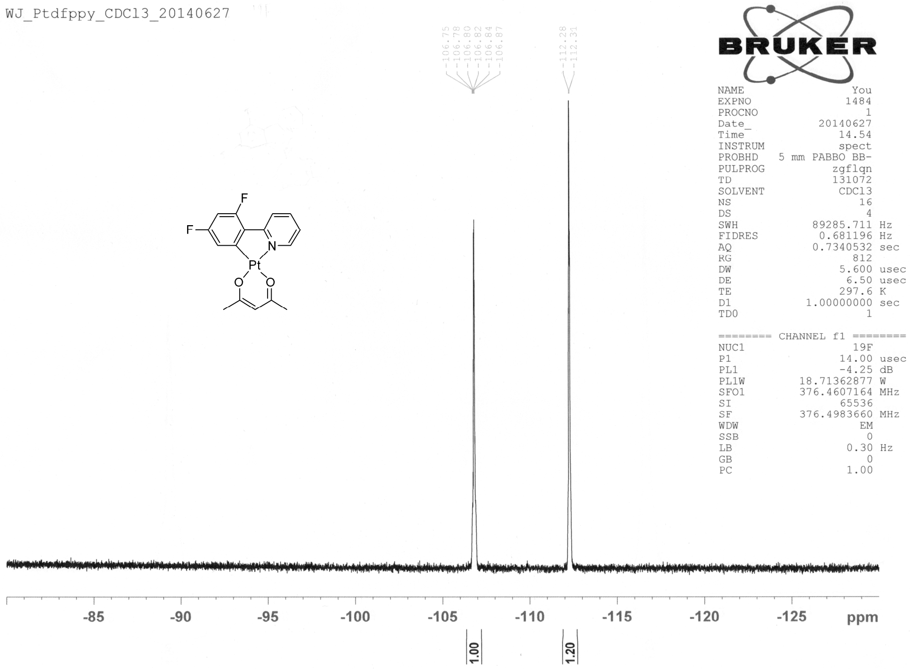

Fig. S13 ^{19}F NMR spectrum (376 MHz, $CDCl_3$) of Ptdfppy.

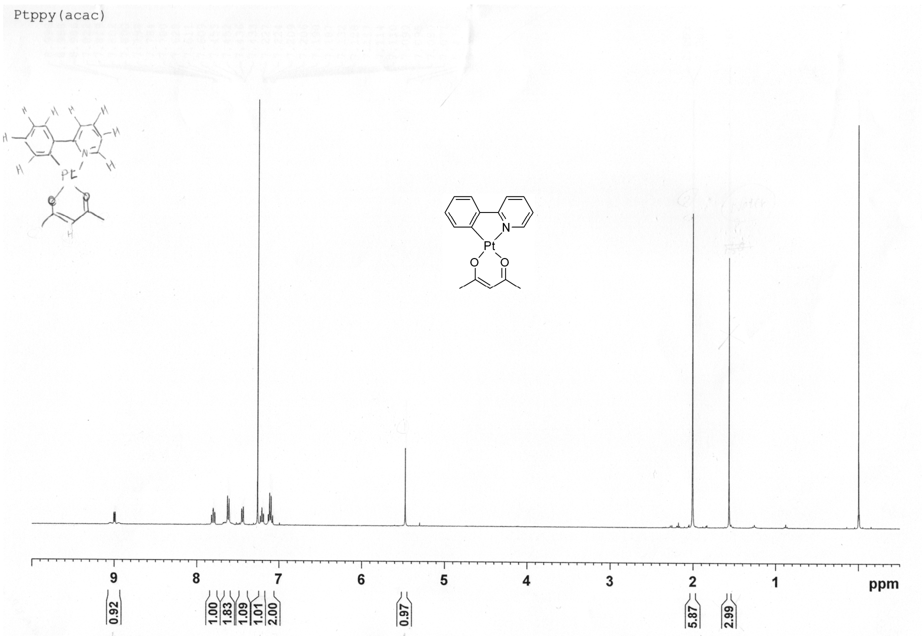

Fig. S14 ^{1}H NMR spectrum (400 MHz, $CDCl_3$) of Ptppy.

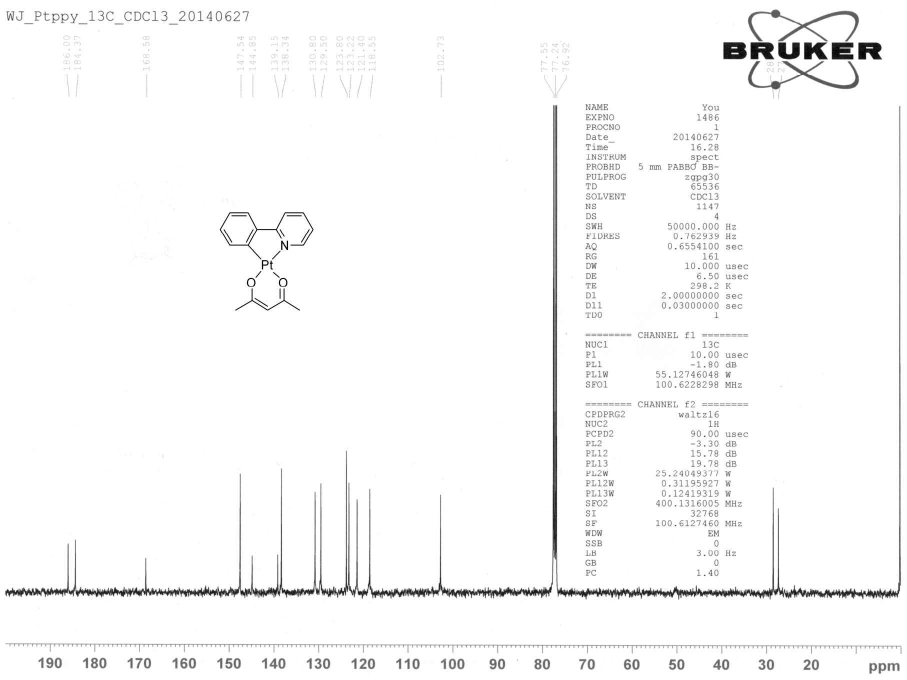

Fig. S15 ^{13}C{^{1}H} NMR spectrum (100 MHz, $CDCl_3$) of Ptppy.

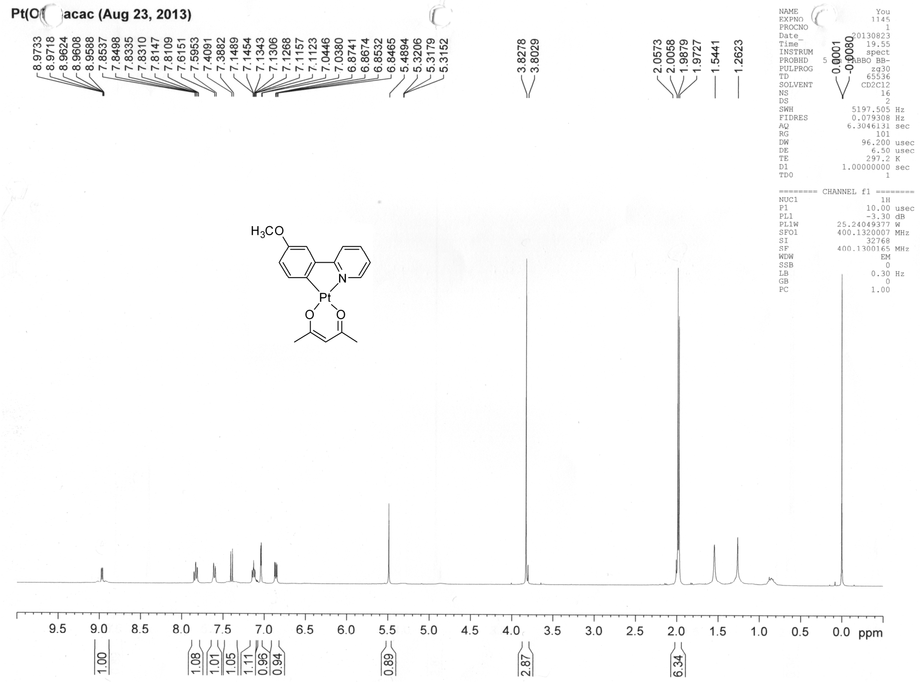

Fig. S16 ^{1}H NMR spectrum (400 MHz, $CDCl_3$) of PtOMe.

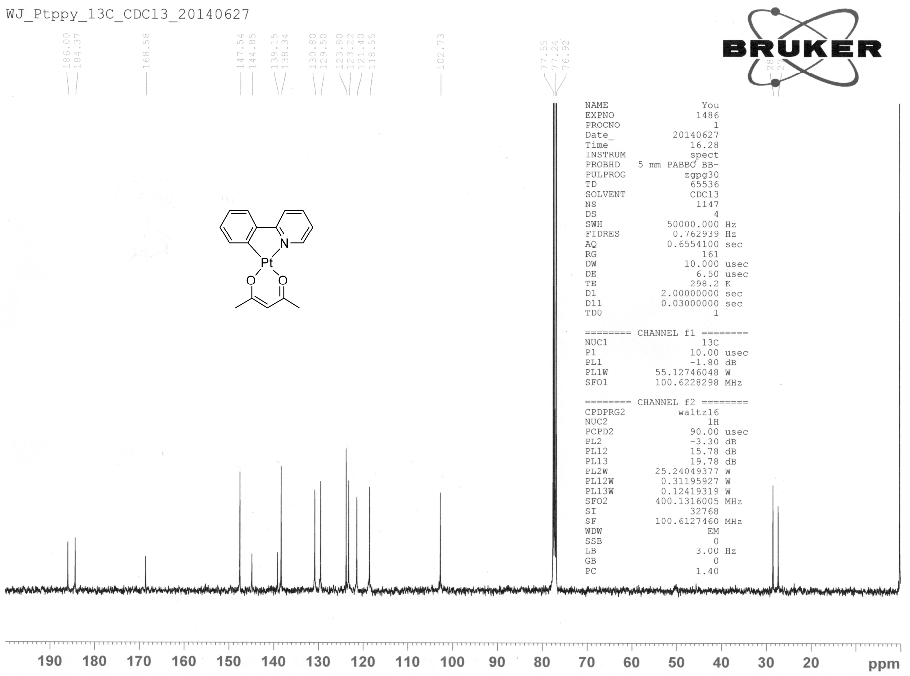

Fig. S17 $^{13}C\{^{1}H\}$ NMR spectrum (100 MHz, $CDCl_3$) of PtOMe.

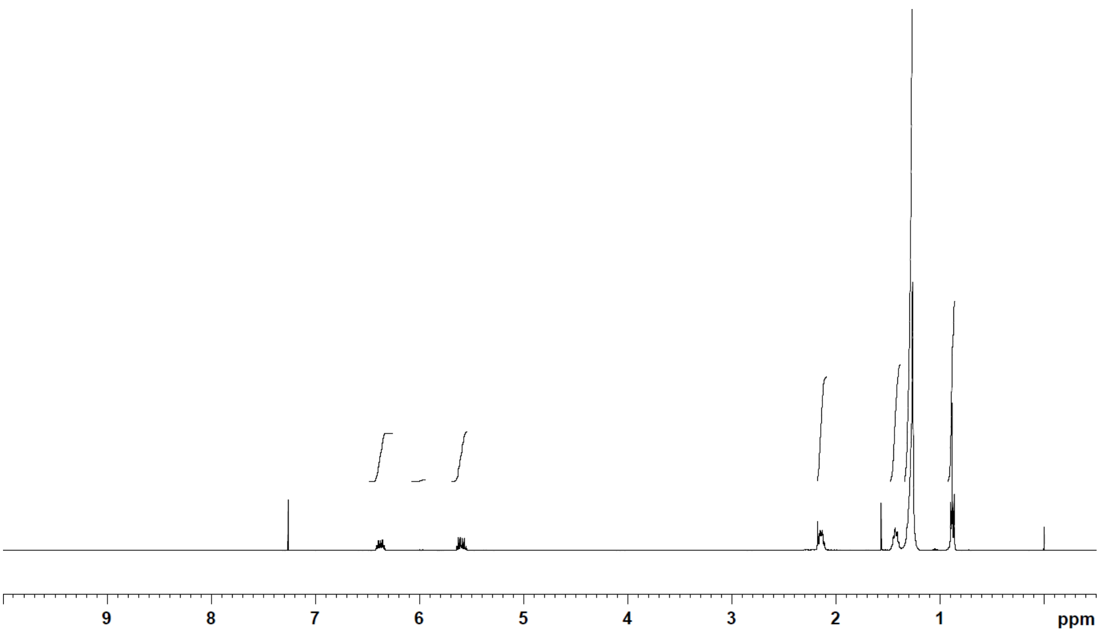

Fig. S18 ^{1}H NMR spectrum (400 MHz, $CDCl_3$) of **2a**.

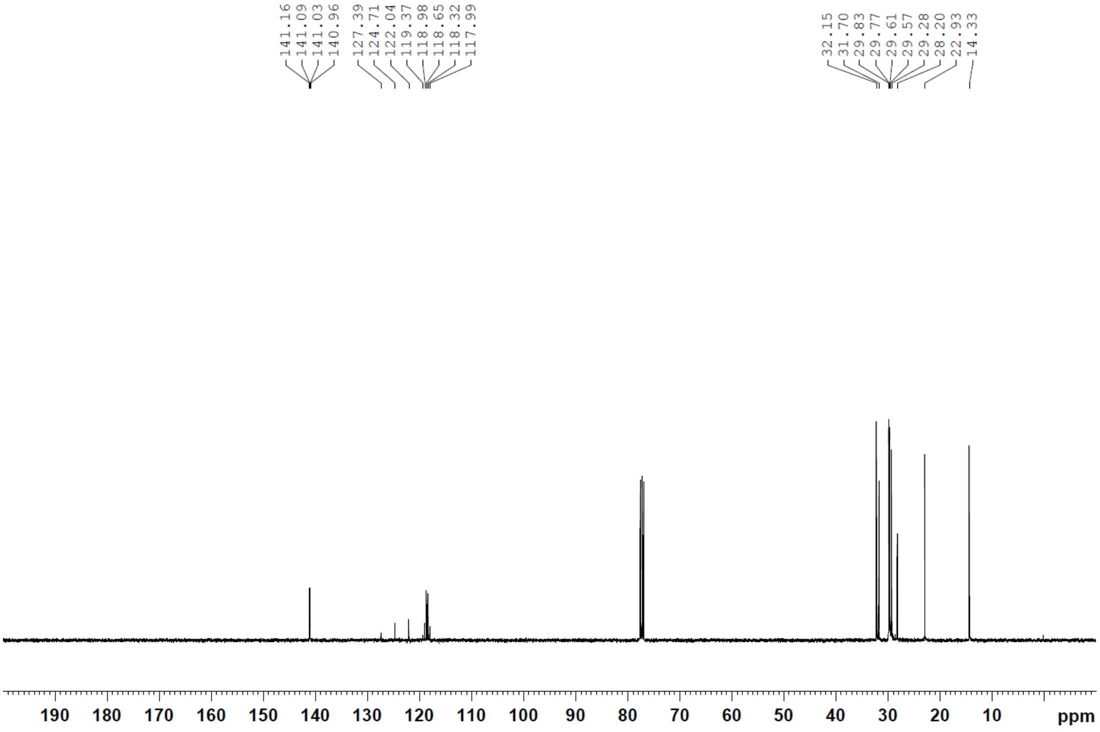

Fig. S19 ^{13}C NMR spectrum (101 MHz, $CDCl_3$) of **2a**.

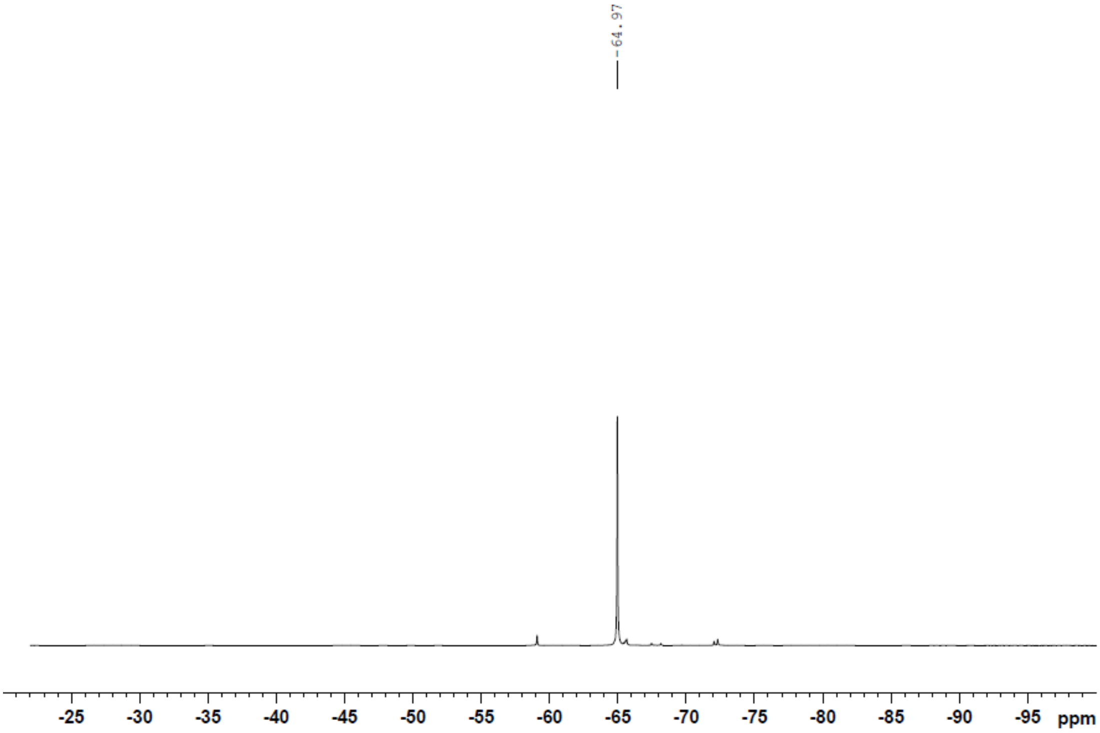

Fig. S20 ^{19}F NMR spectrum (377 MHz, $CDCl_3$) of **2a**.

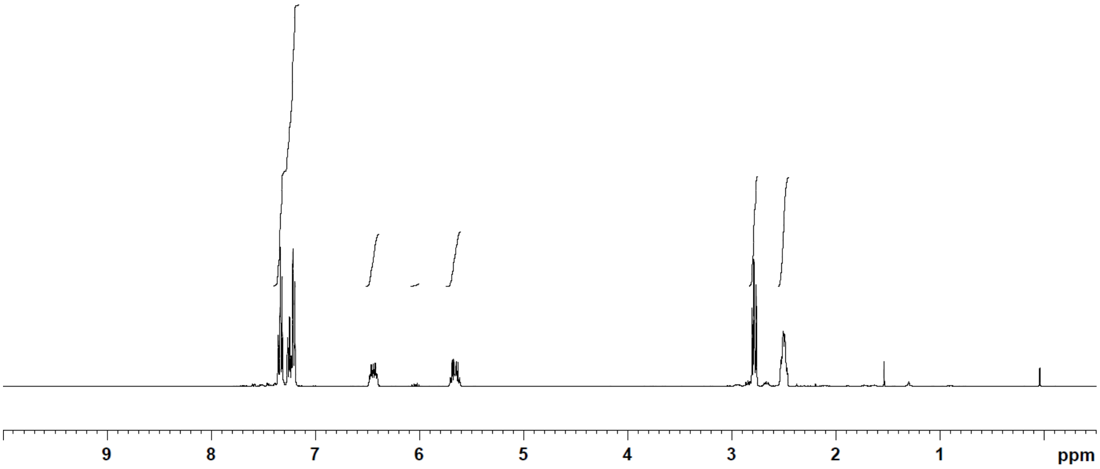

Fig. S21 ^{1}H NMR spectrum (400 MHz, $CDCl_3$) of **2b**.

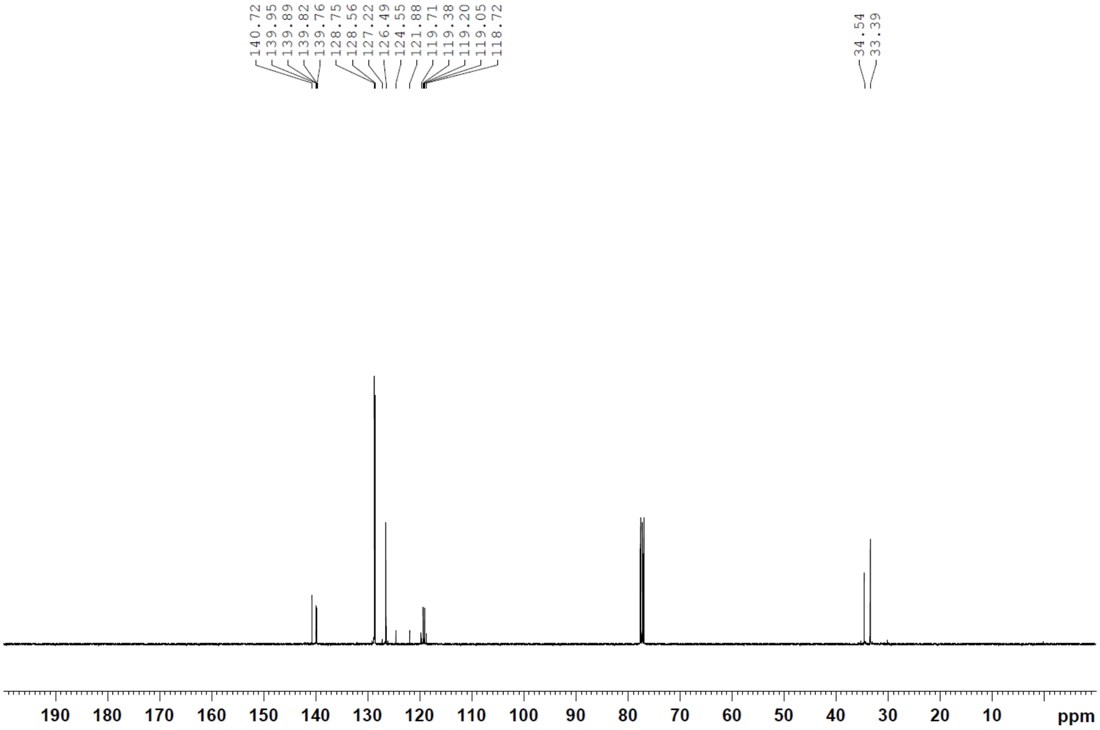

Fig. S22 ^{13}C NMR spectrum (101 MHz, $CDCl_3$) of **2b**.

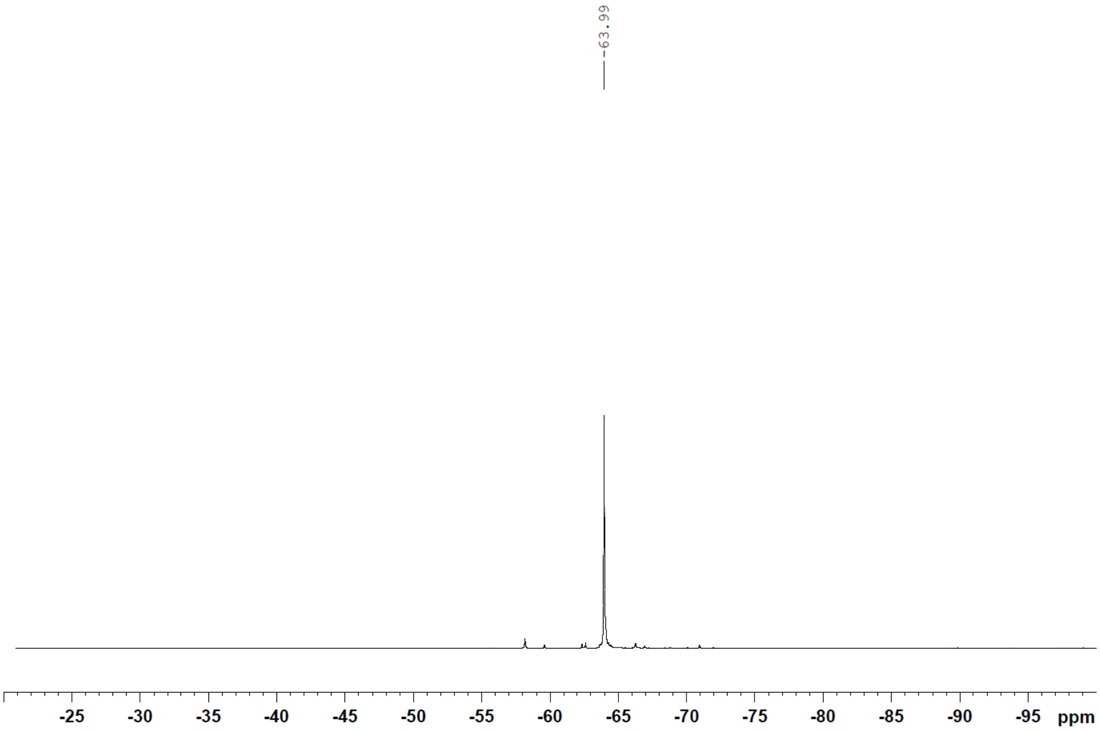

Fig. S23 ^{19}F NMR spectrum (377 MHz, $CDCl_3$) of **2b**.

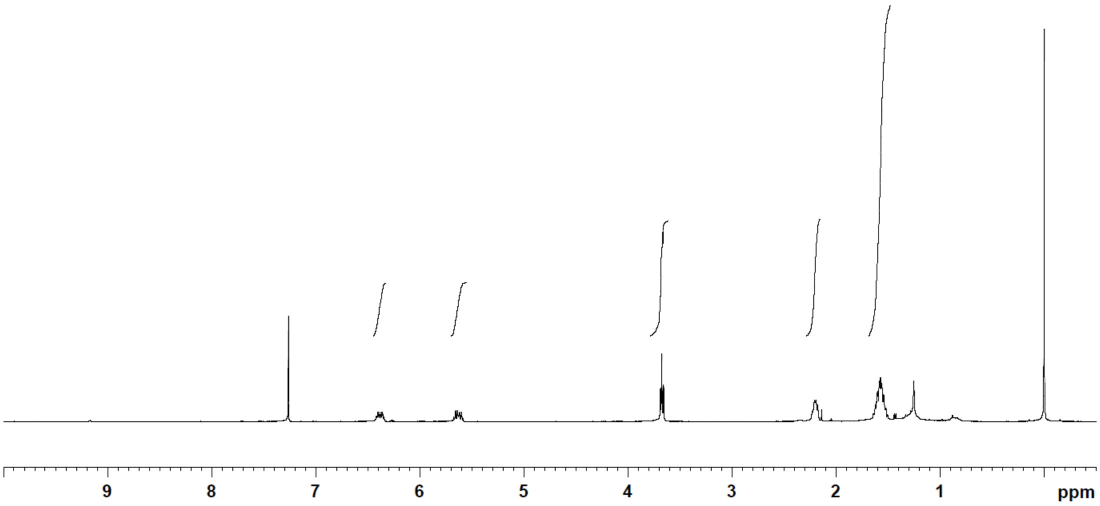

Fig. S24 ^{1}H NMR spectrum (400 MHz, $CDCl_3$) of **2c**.

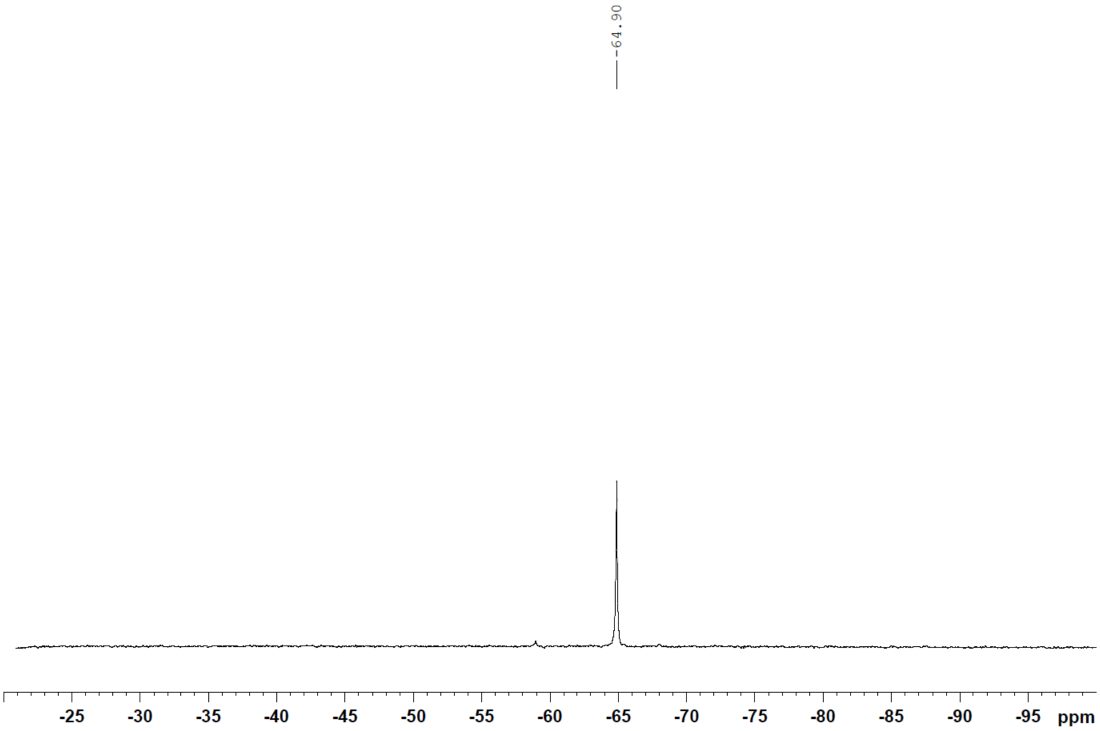

Fig. S25 ^{19}F NMR spectrum (377 MHz, $CDCl_3$) of **2c**.

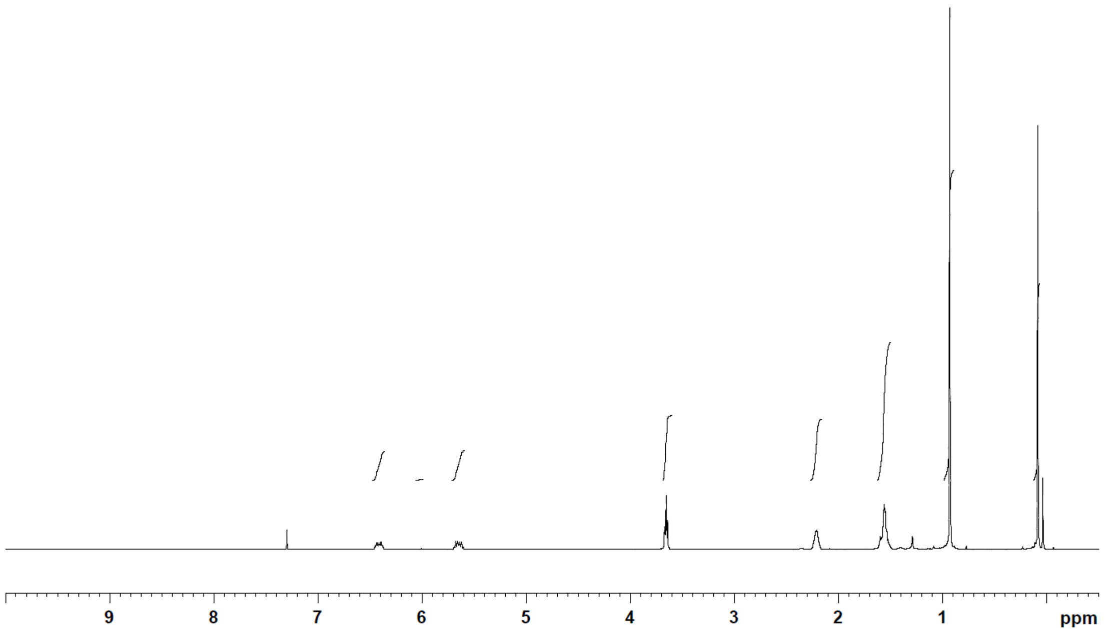

Fig. S26 ^{1}H NMR spectrum (400 MHz, $CDCl_3$) of **2d**.

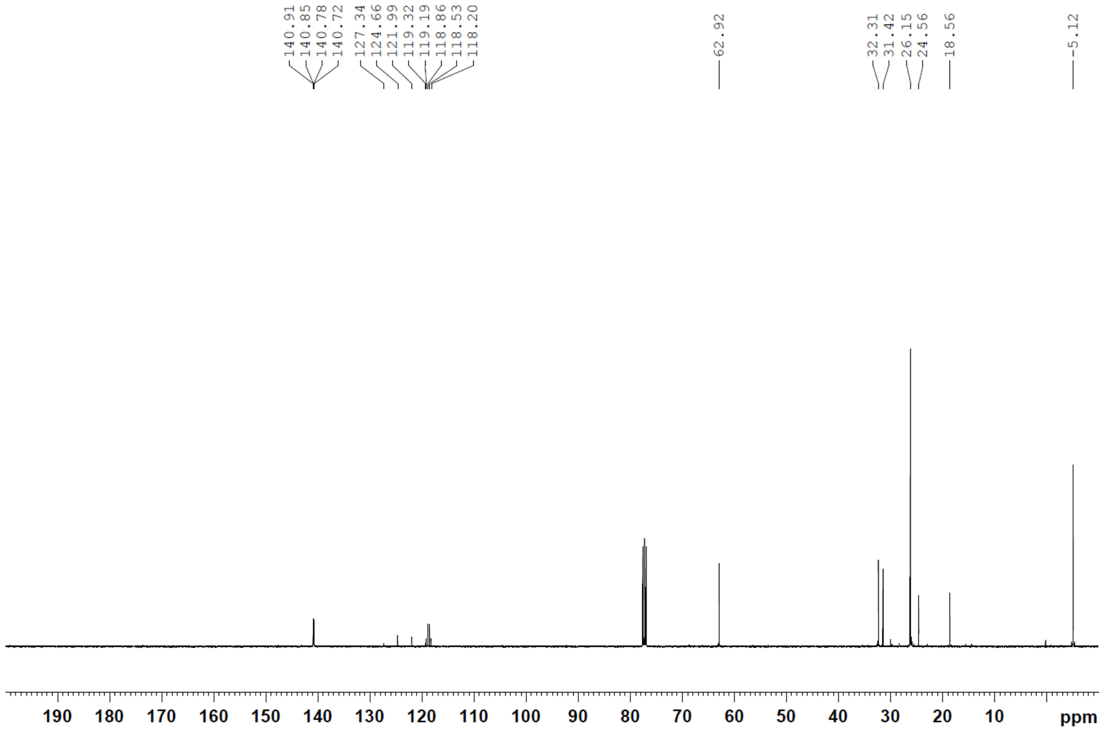

Fig. S27 ^{13}C NMR spectrum (101 MHz, $CDCl_3$) of **2d**.

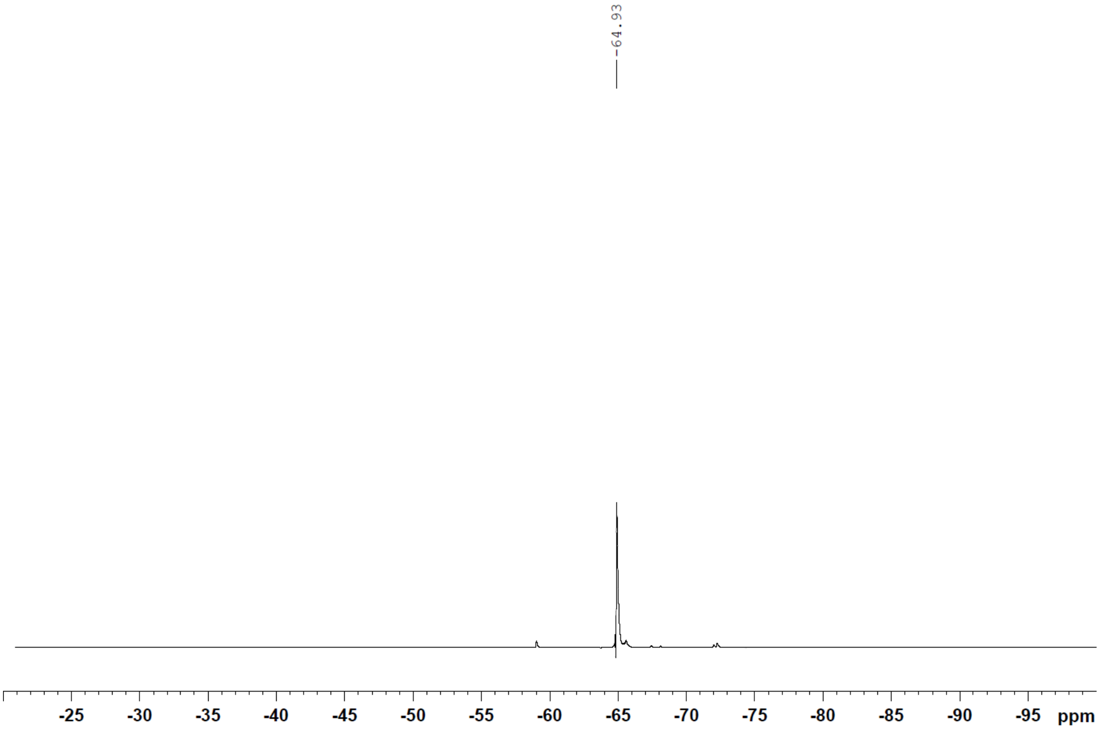

Fig. S28 ^{19}F NMR spectrum (377 MHz, $CDCl_3$) of **2d**.

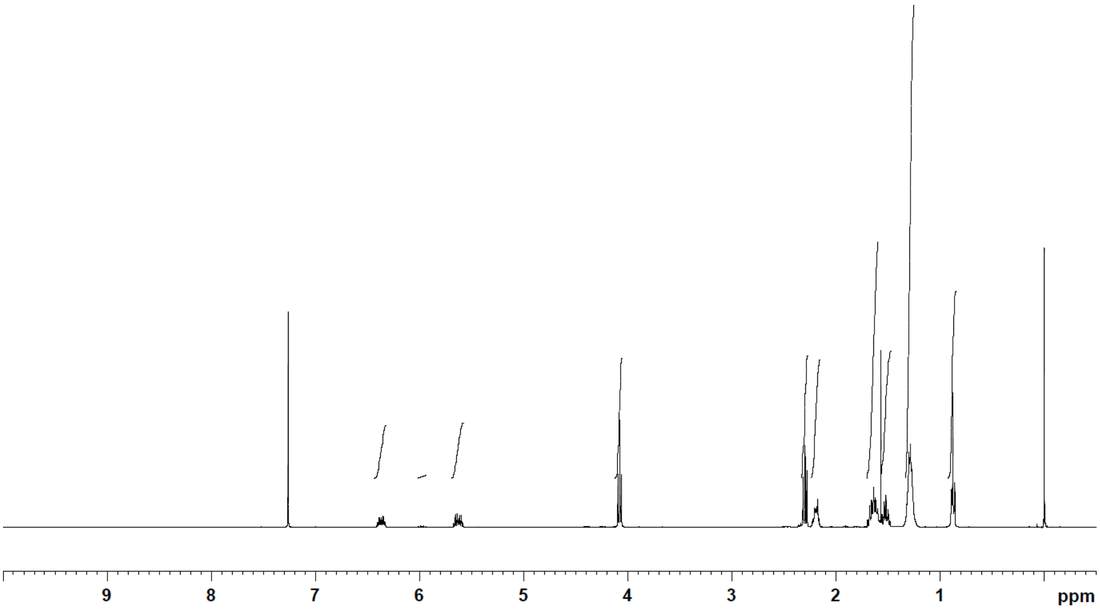

Fig. S29 ^{1}H NMR spectrum (400 MHz, $CDCl_3$) of **2e**.

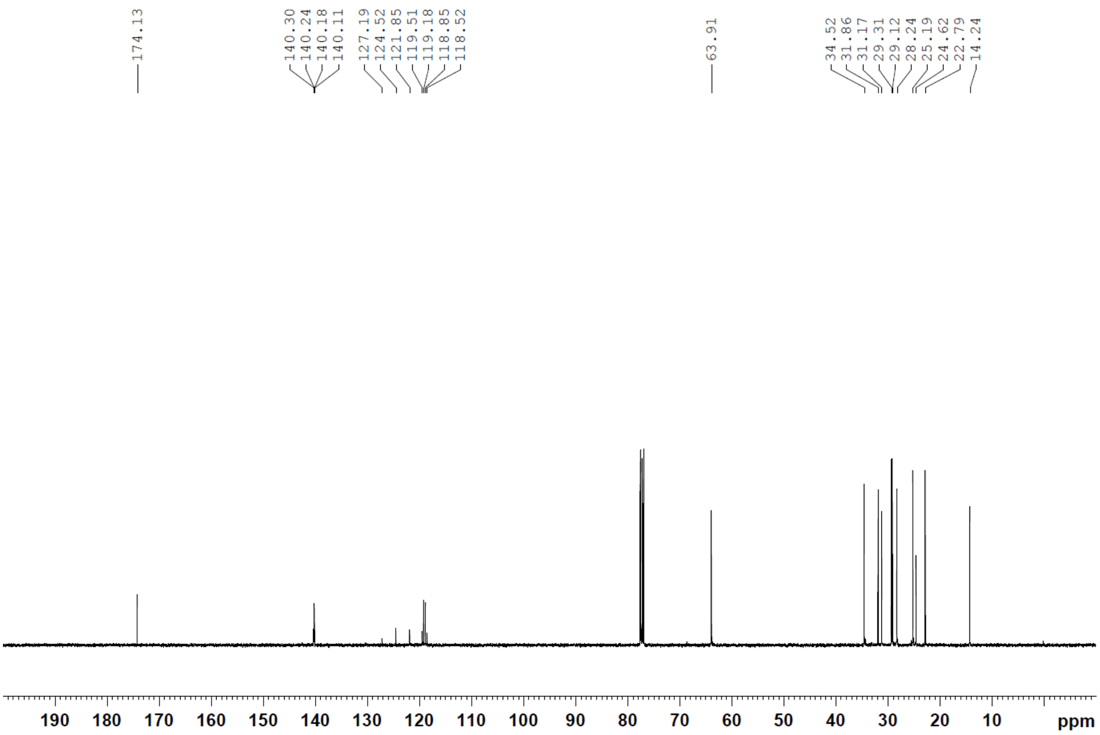

Fig. S30 ^{13}C NMR spectrum (101 MHz, $CDCl_3$) of **2e**.

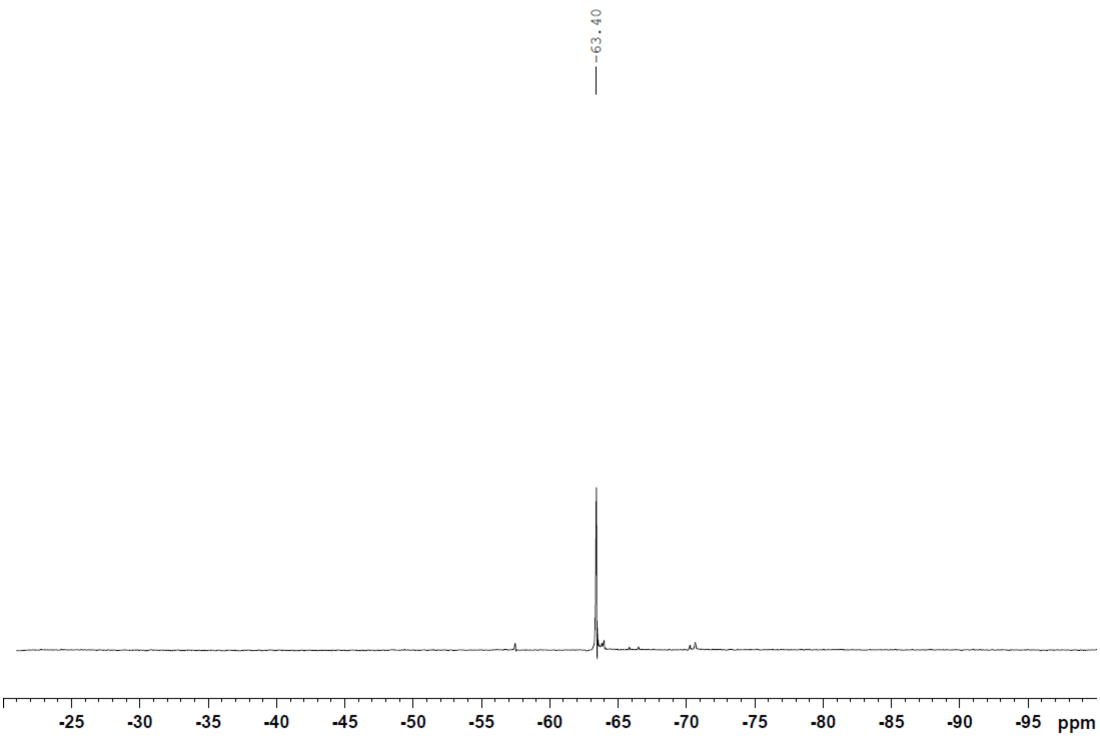

Fig. S31 ^{19}F NMR spectrum (377 MHz, $CDCl_3$) of **2e**.

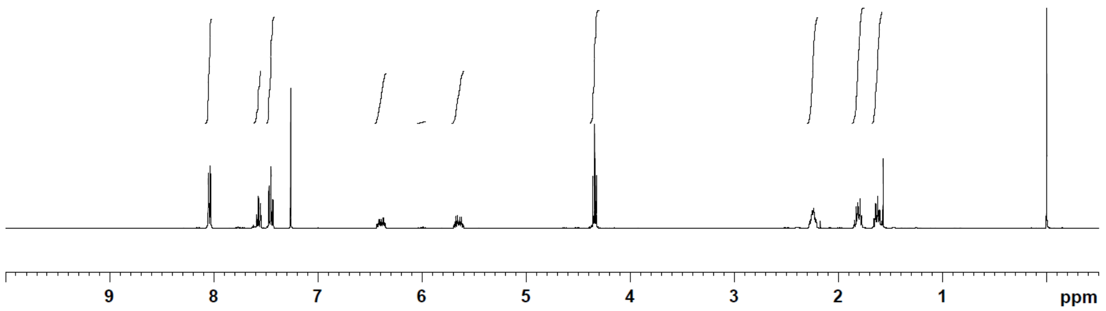

Fig. S32 ^{1}H NMR spectrum (400 MHz, $CDCl_3$) of **2f**.

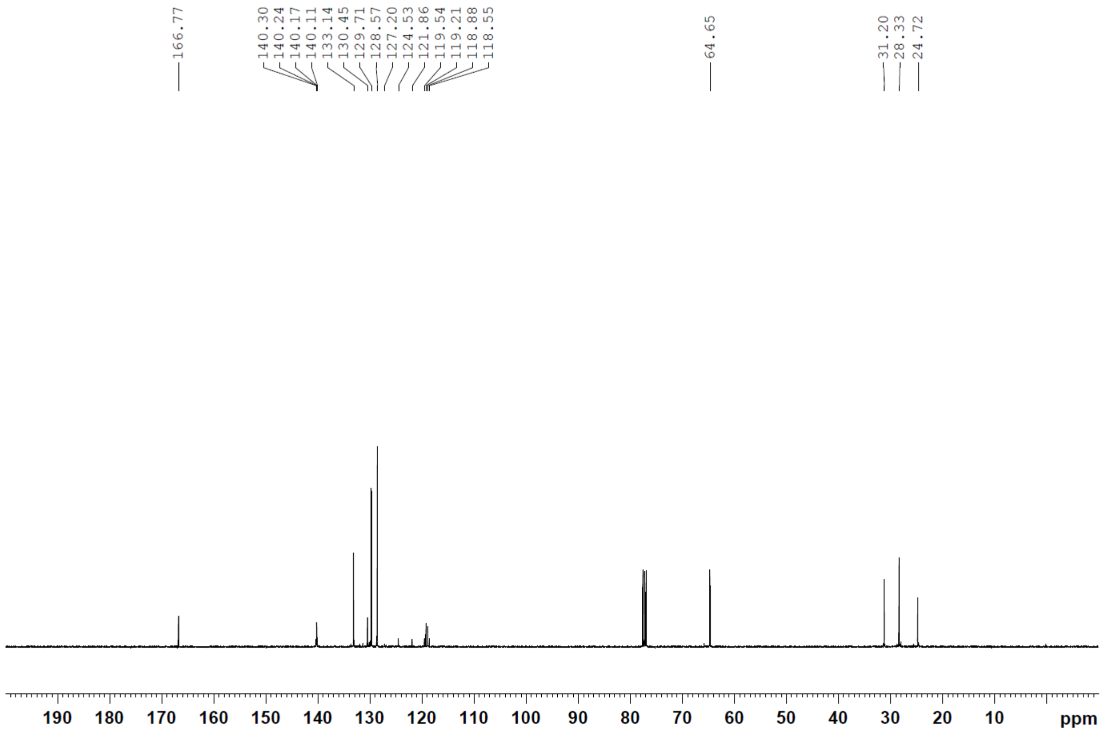

Fig. S33 ^{13}C NMR spectrum (101 MHz, $CDCl_3$) of **2f**.

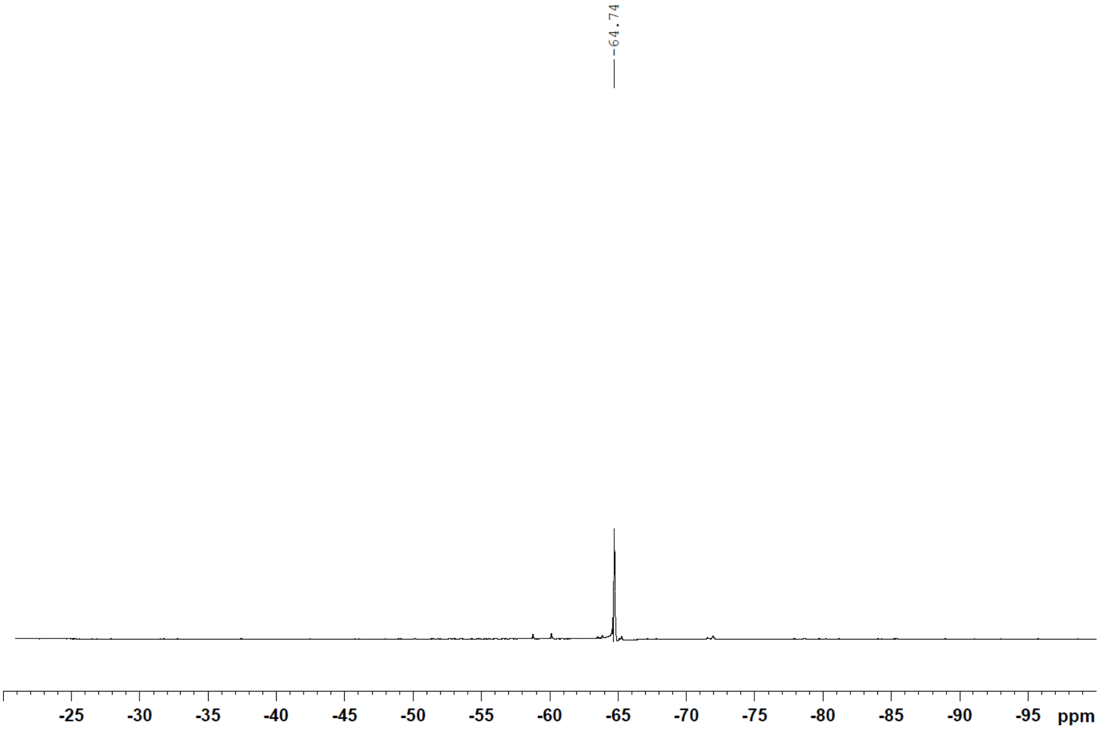

Fig. S34 ^{19}F NMR spectrum (377 MHz, $CDCl_3$) of **2f**.

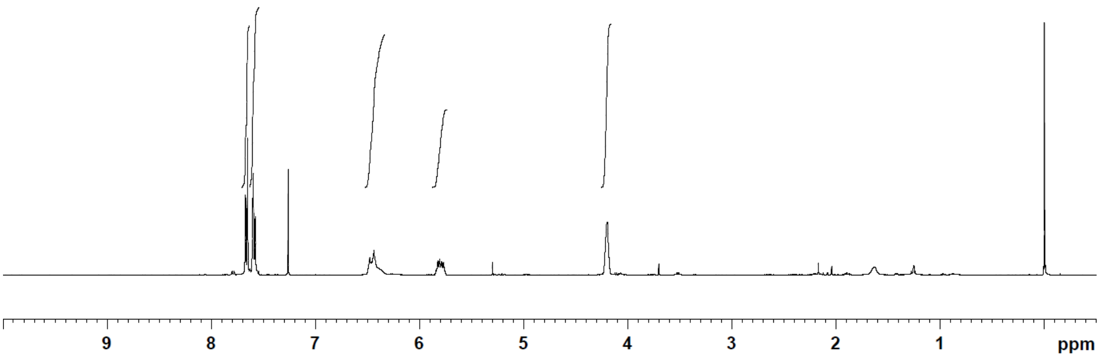

Fig. S35 ^{1}H NMR spectrum (400 MHz, $CDCl_3$) of **2g**.

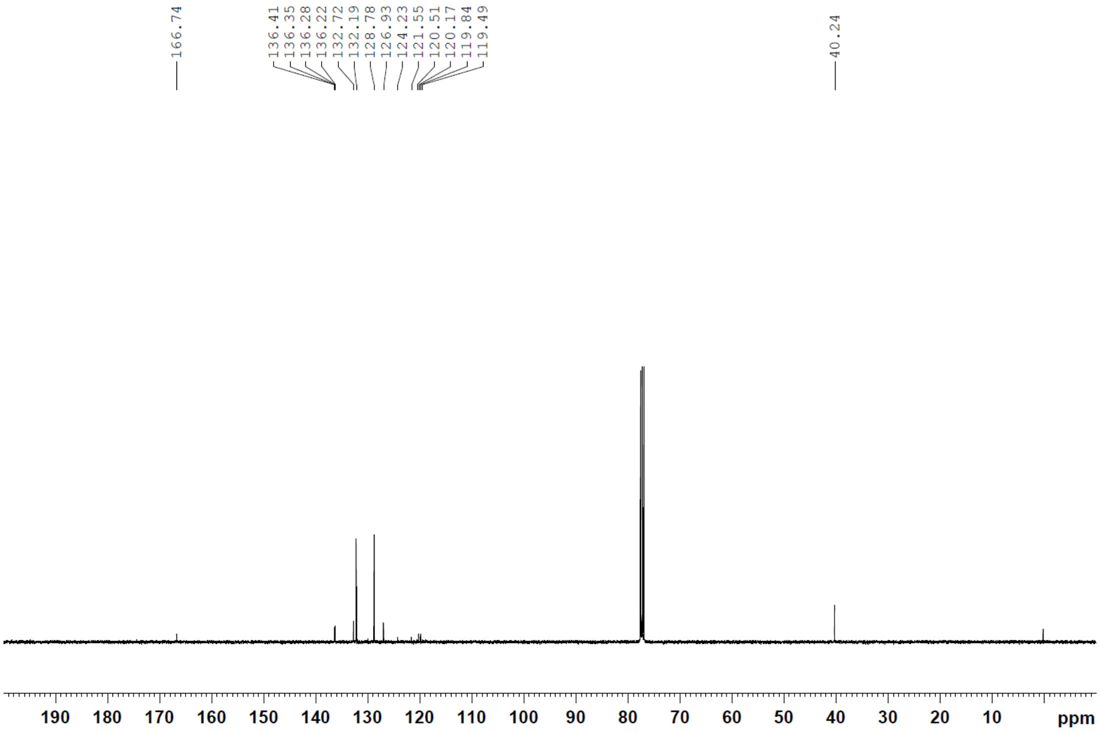

Fig. S36 ^{13}C NMR spectrum (101 MHz, $CDCl_3$) of **2g**.

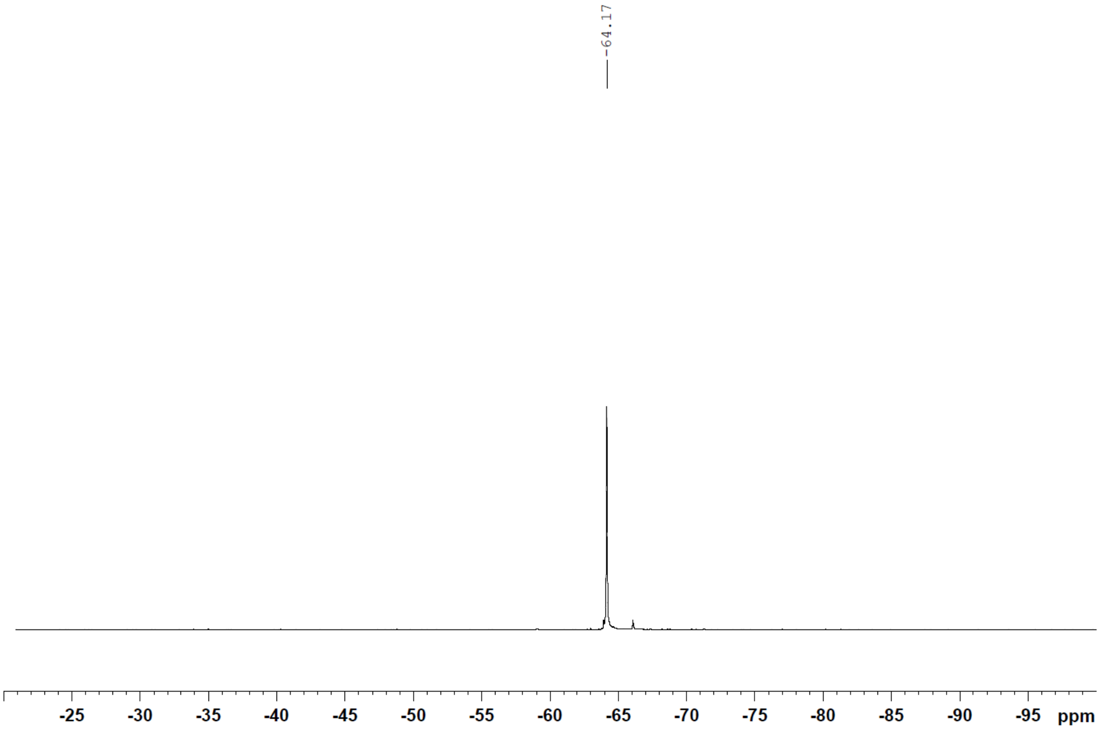

Fig. S37 ^{19}F NMR spectrum (377 MHz, $CDCl_3$) of **2g**.

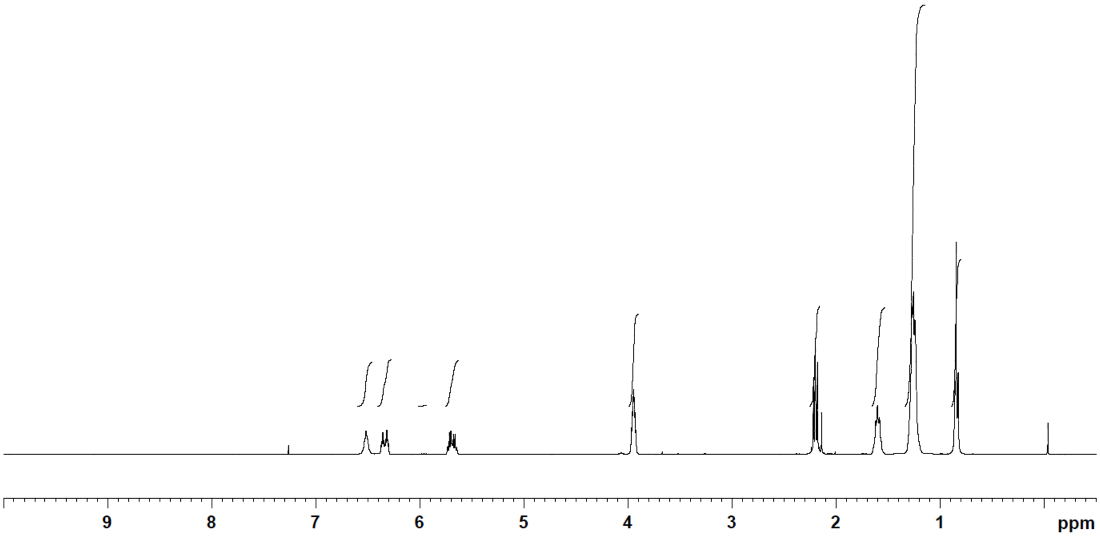

Fig. S38 ^{1}H NMR spectrum (400 MHz, $CDCl_3$) of **2h**.

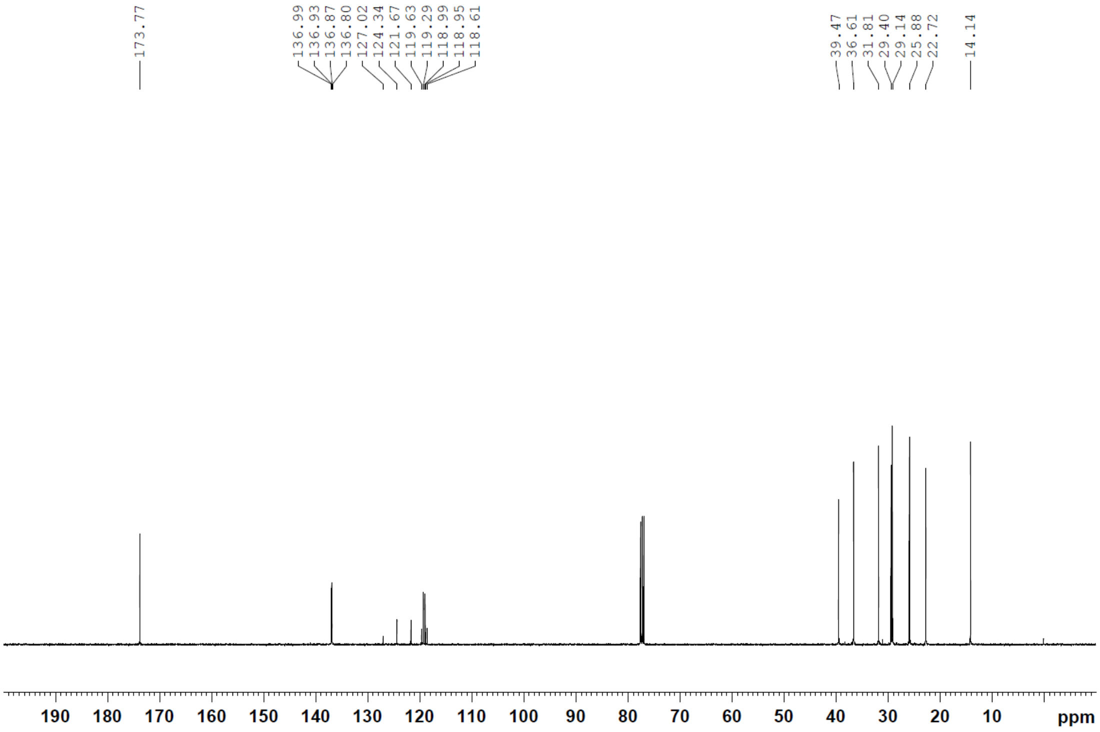

Fig. S39 ^{13}C NMR spectrum (101 MHz, $CDCl_3$) of **2h**.

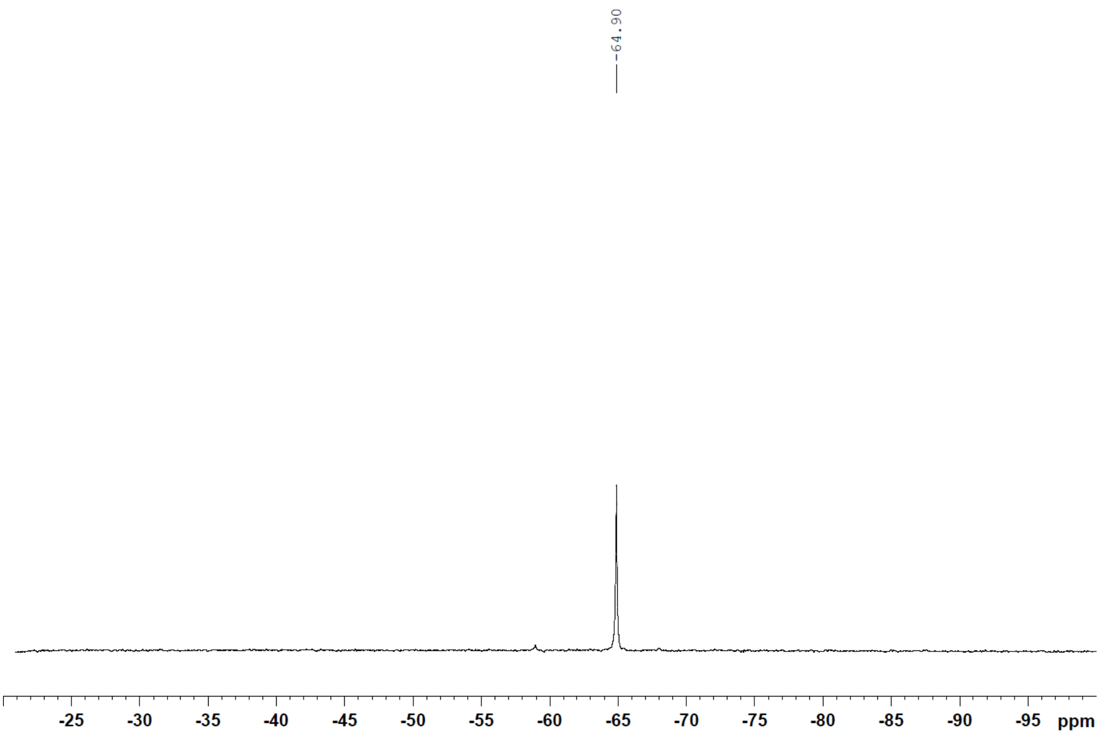

Fig. S40 ^{19}F NMR spectrum (377 MHz, $CDCl_3$) of **2h**.

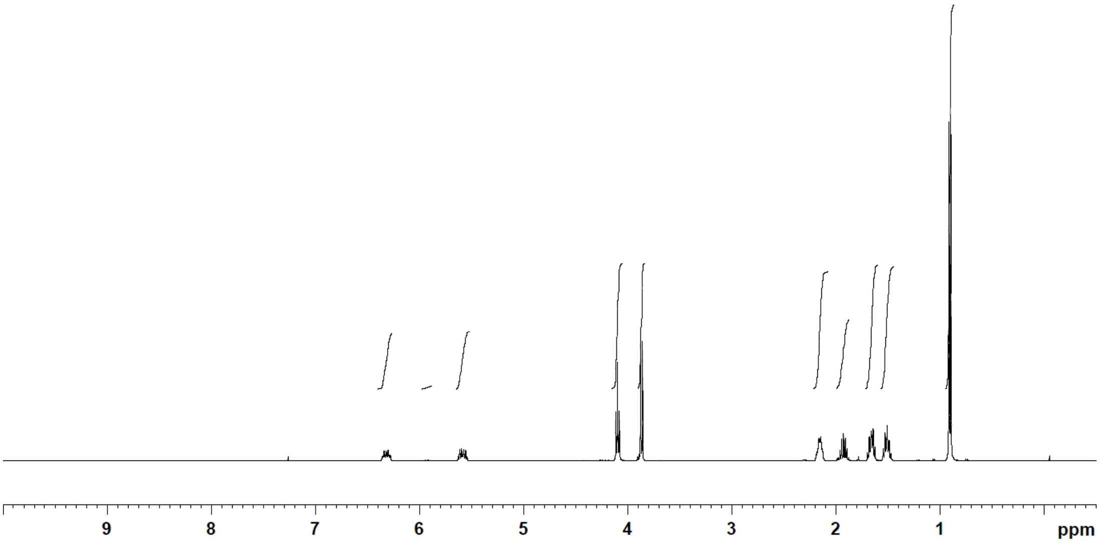

Fig. S41 ^{1}H NMR spectrum (400 MHz, $CDCl_3$) of **2i**.

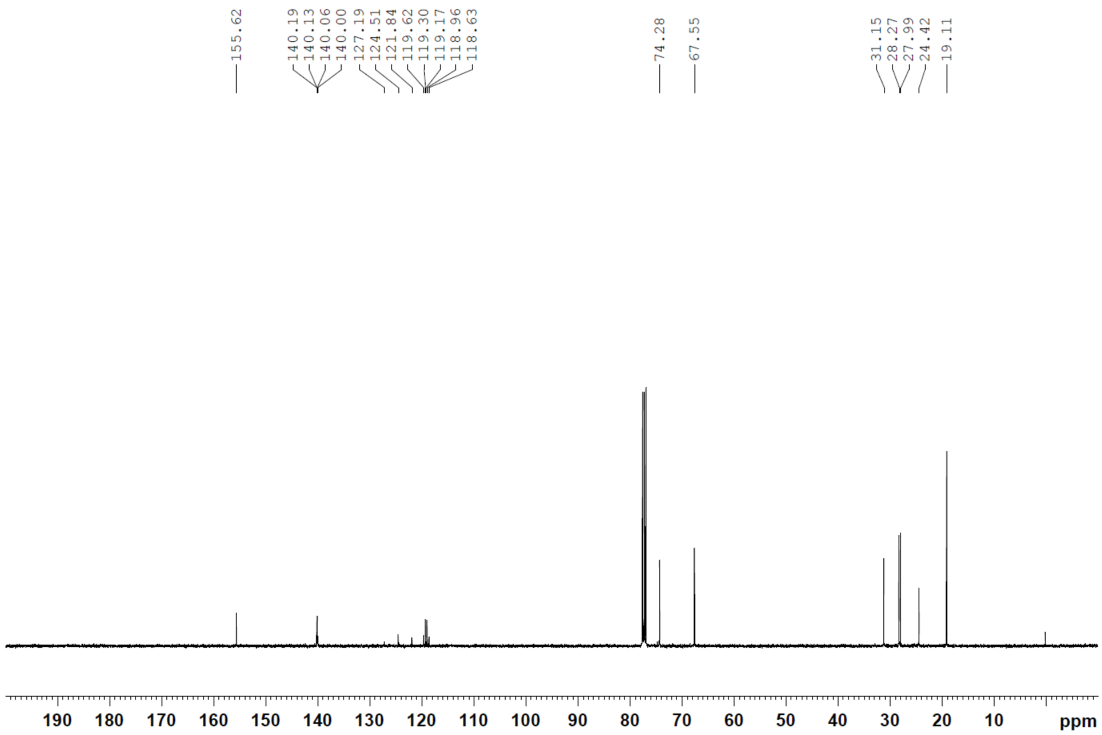

Fig. S42 ^{13}C NMR spectrum (101 MHz, $CDCl_3$) of **2i**.

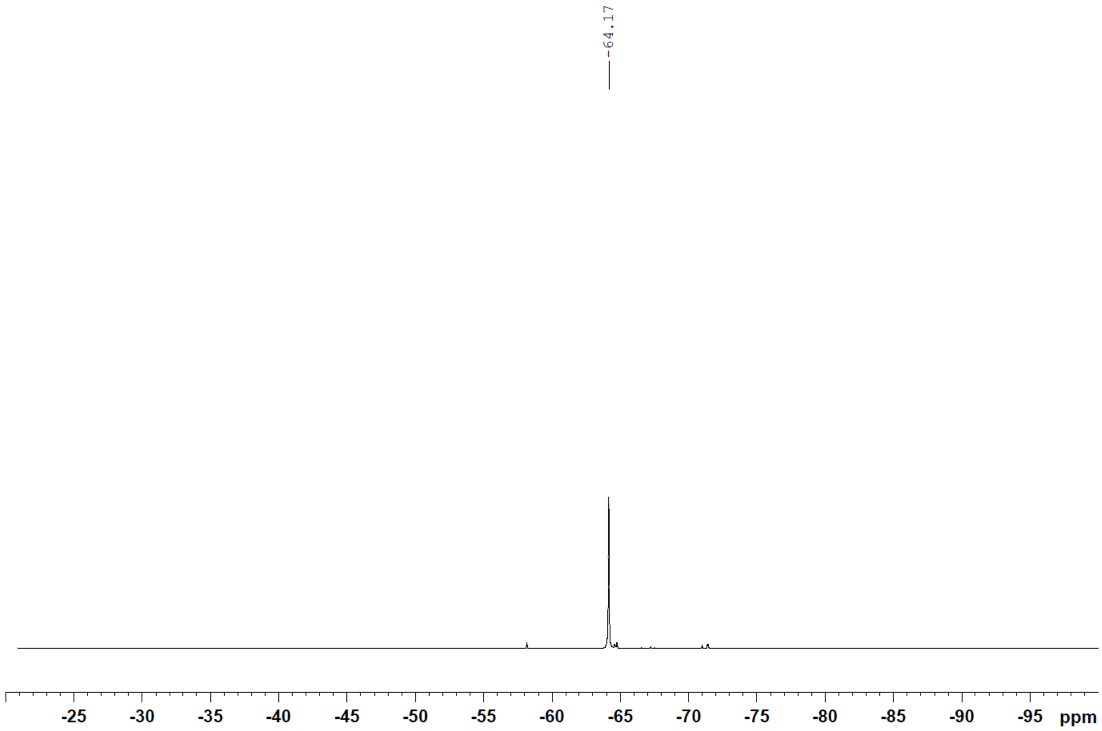

Fig. S43 ^{19}F NMR spectrum (377 MHz, $CDCl_3$) of **2i**.

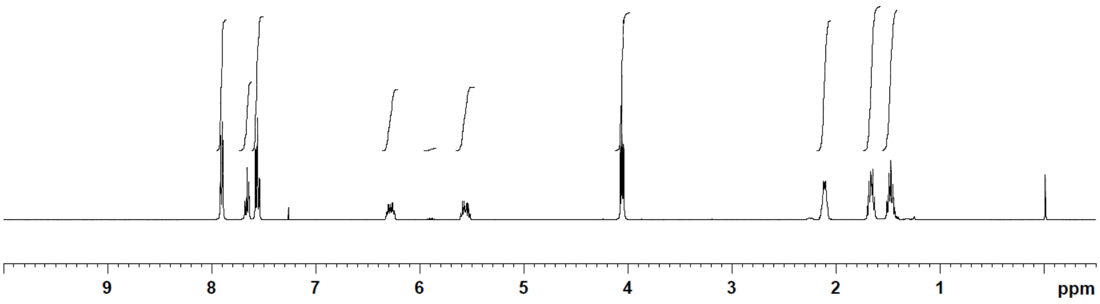

Fig. S44 ^{1}H NMR spectrum (400 MHz, $CDCl_3$) of **2j**.

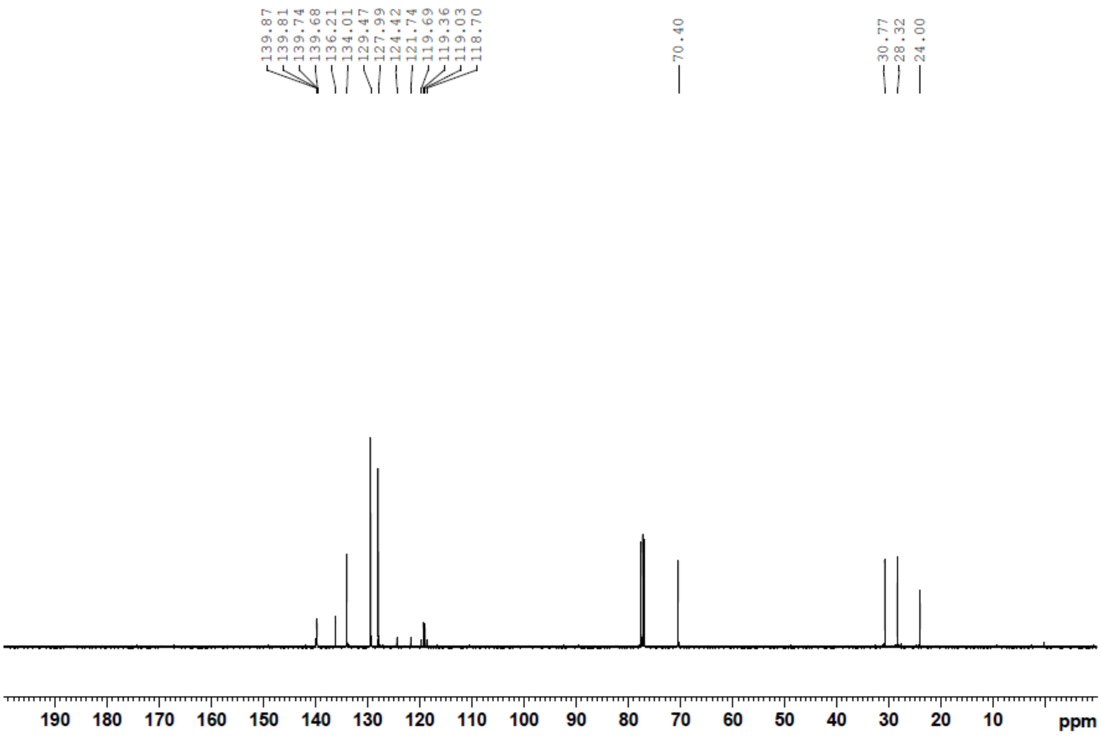

Fig. S45 ^{13}C NMR spectrum (101 MHz, $CDCl_3$) of **2j**.

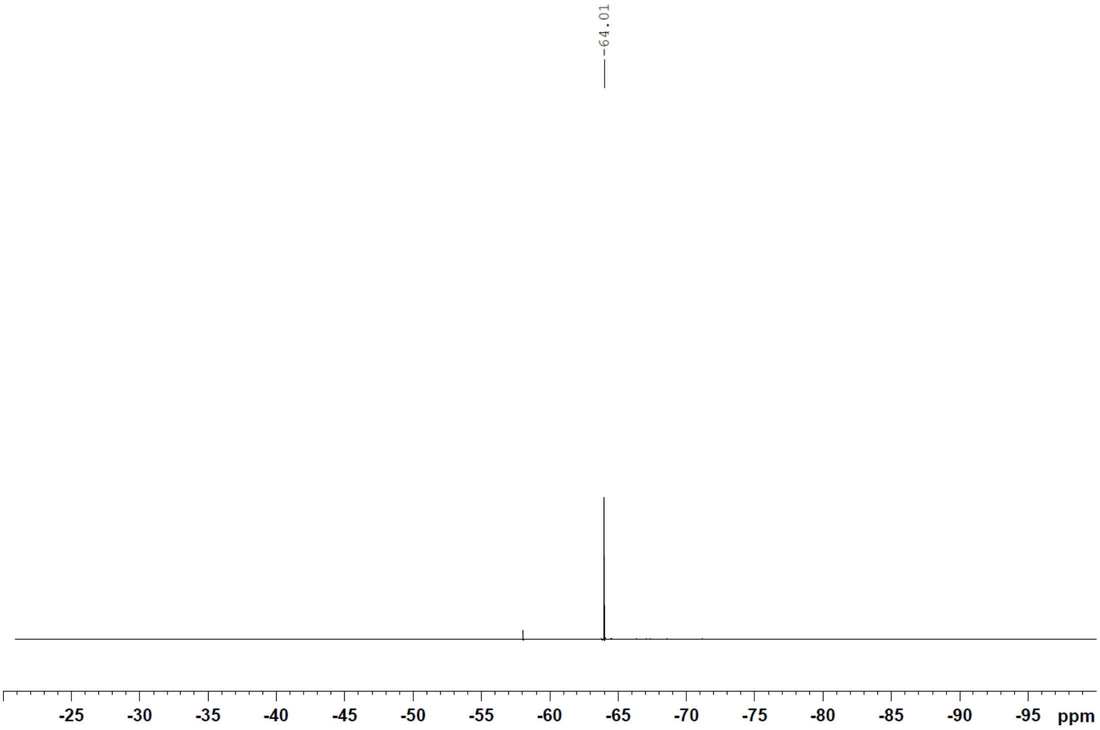

Fig. S46 ^{19}F NMR spectrum (377 MHz, $CDCl_3$) of **2j**.

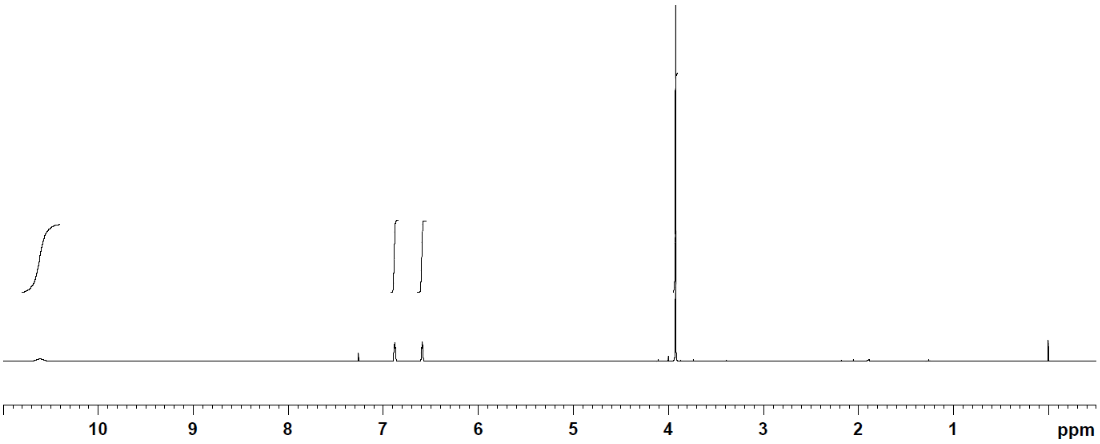

Fig. S47 ^{1}H NMR spectrum (400 MHz, $CDCl_3$) of **4b**.

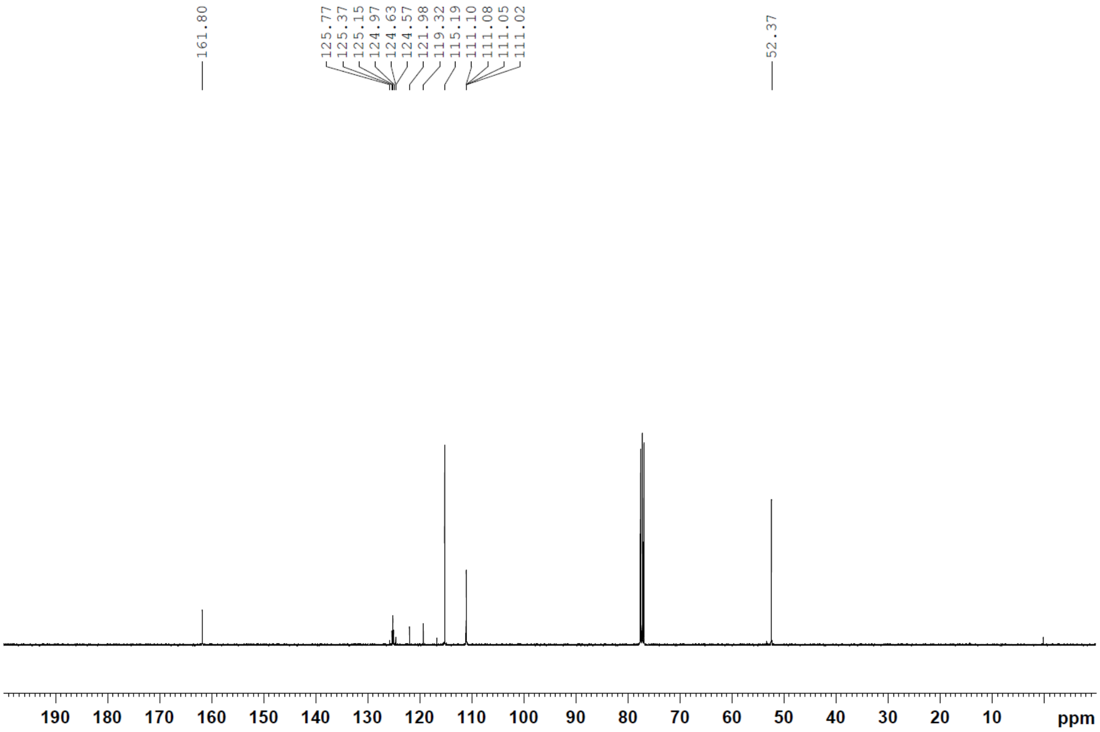

Fig. S48 ^{13}C NMR spectrum (101 MHz, $CDCl_3$) of **4b**.

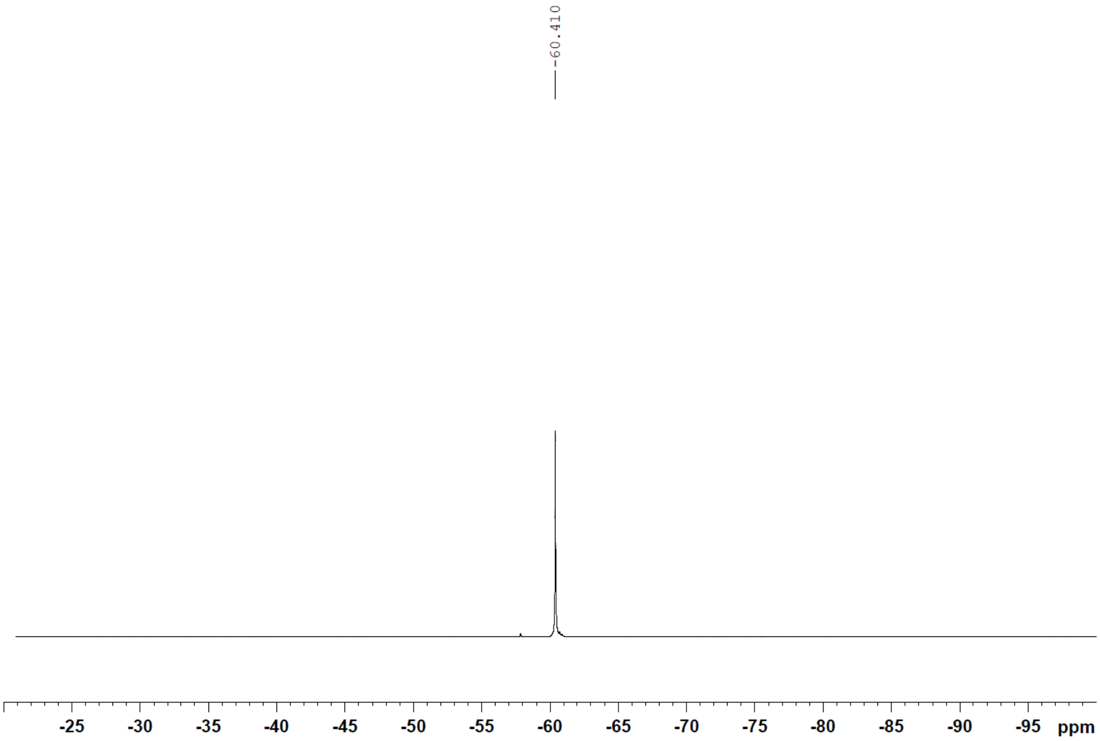

Fig. S49 ^{19}F NMR spectrum (377 MHz, $CDCl_3$) of **4b**.

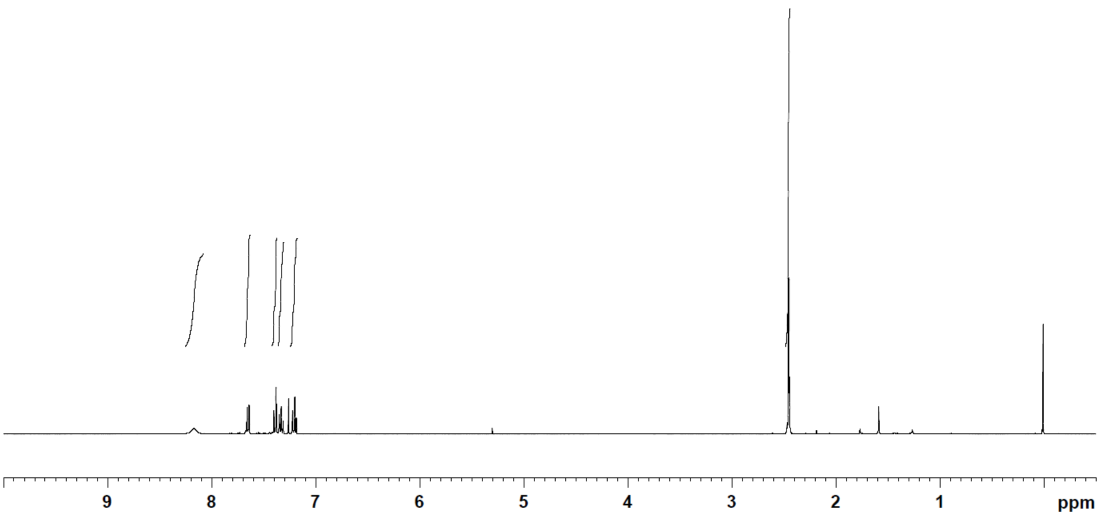

Fig. S50 ^{1}H NMR spectrum (400 MHz, $CDCl_3$) of **4c**.

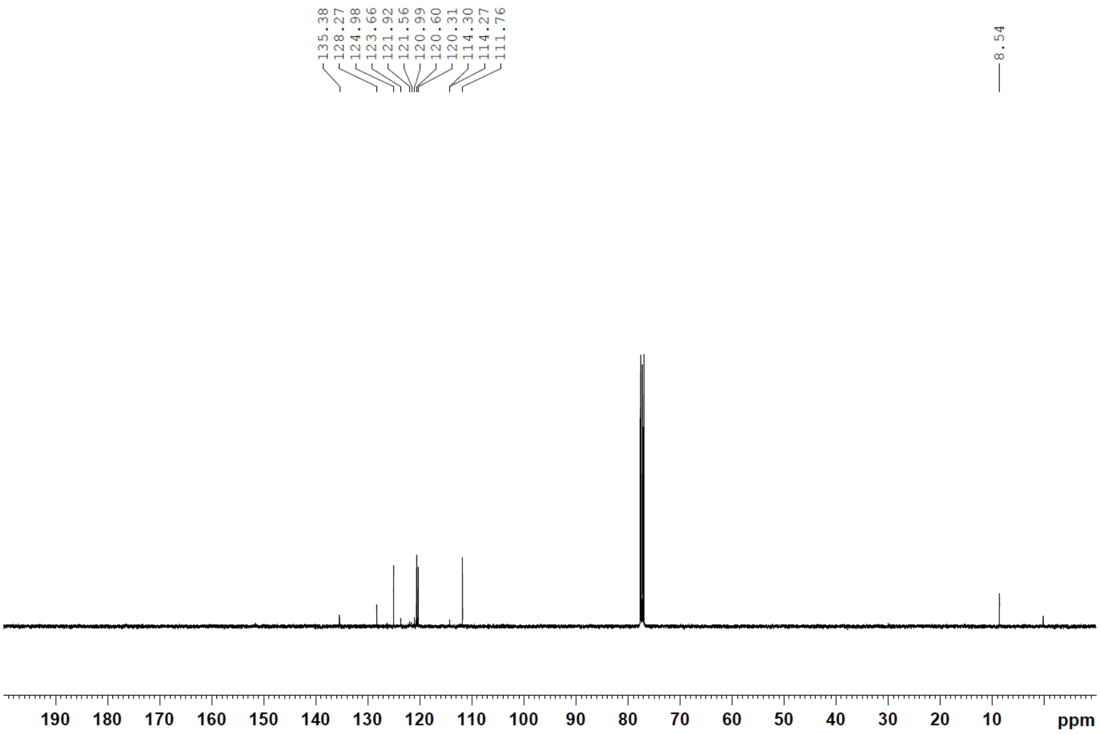

Fig. S51 ^{13}C NMR spectrum (101 MHz, $CDCl_3$) of **4c**.

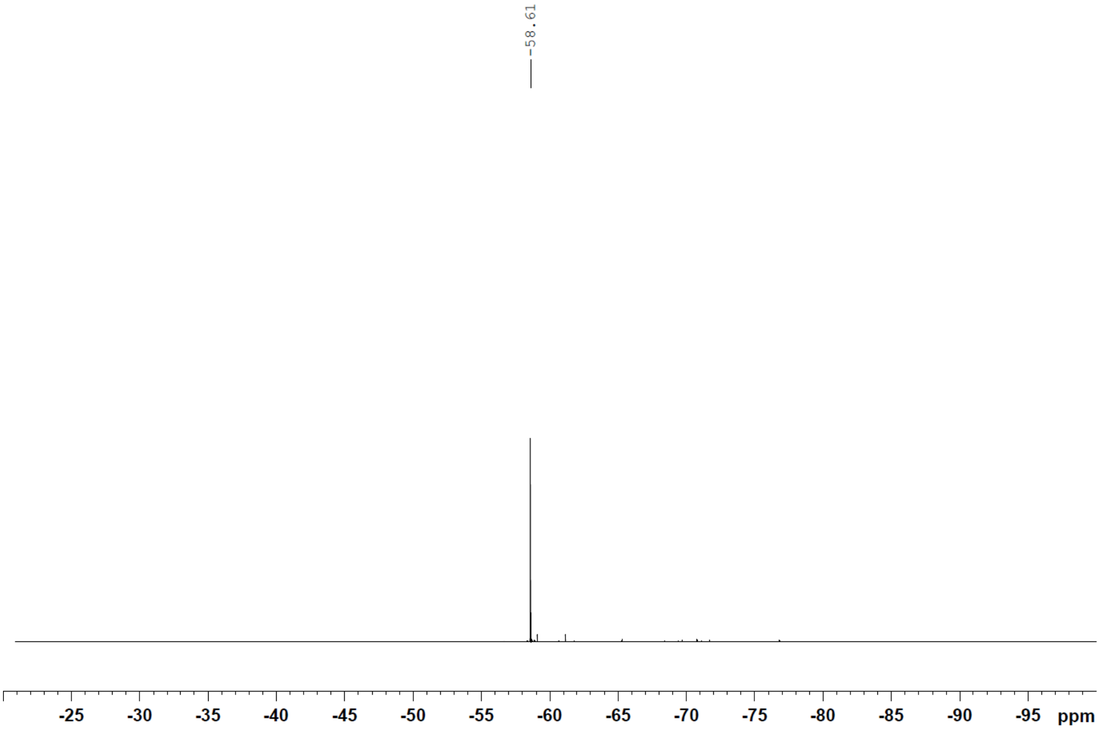

Fig. S52 ^{19}F NMR spectrum (377 MHz, $CDCl_3$) of **4c**.

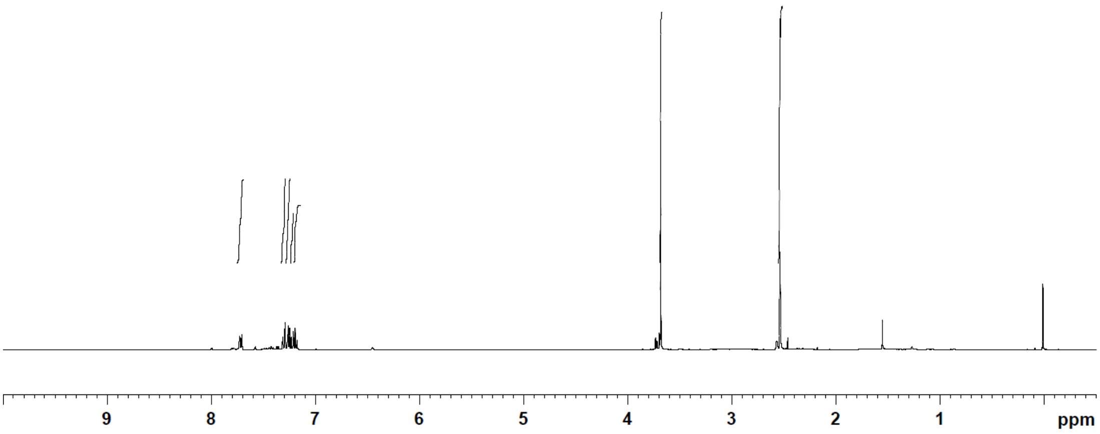

Fig. S53 ^{1}H NMR spectrum (400 MHz, $CDCl_3$) of **4e**.

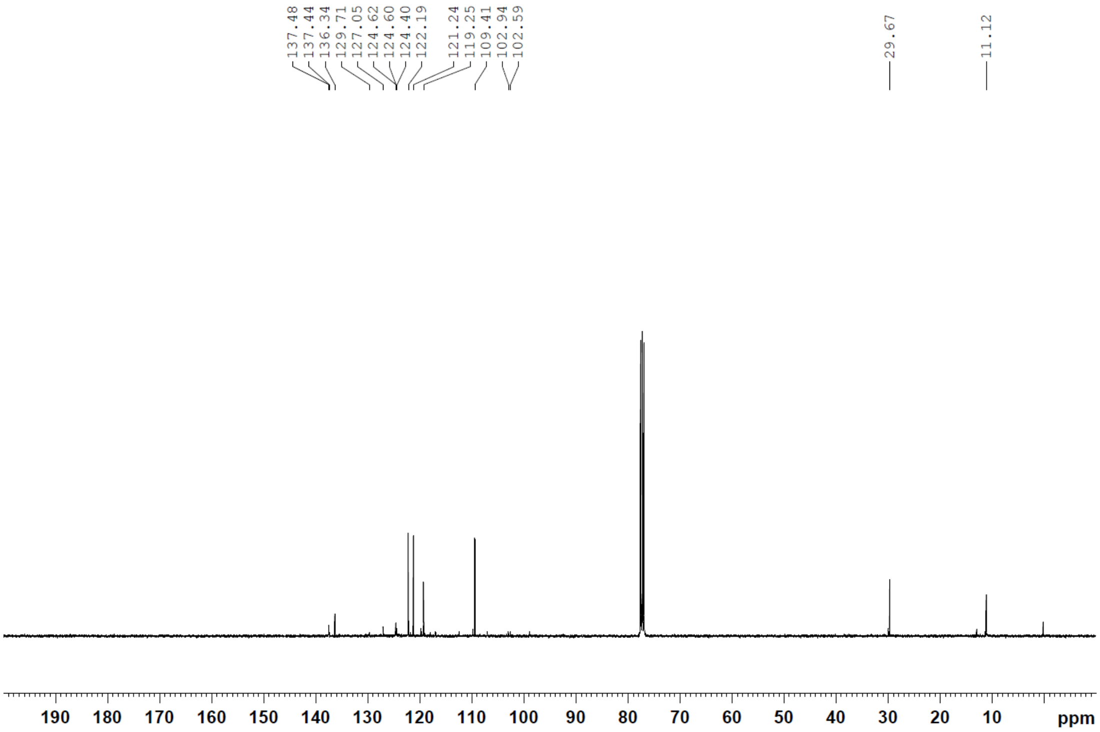

Fig. S54 ^{13}C NMR spectrum (101 MHz, $CDCl_3$) of **4e** (in a 9:1 ratio with bistrifluoromethylated product).

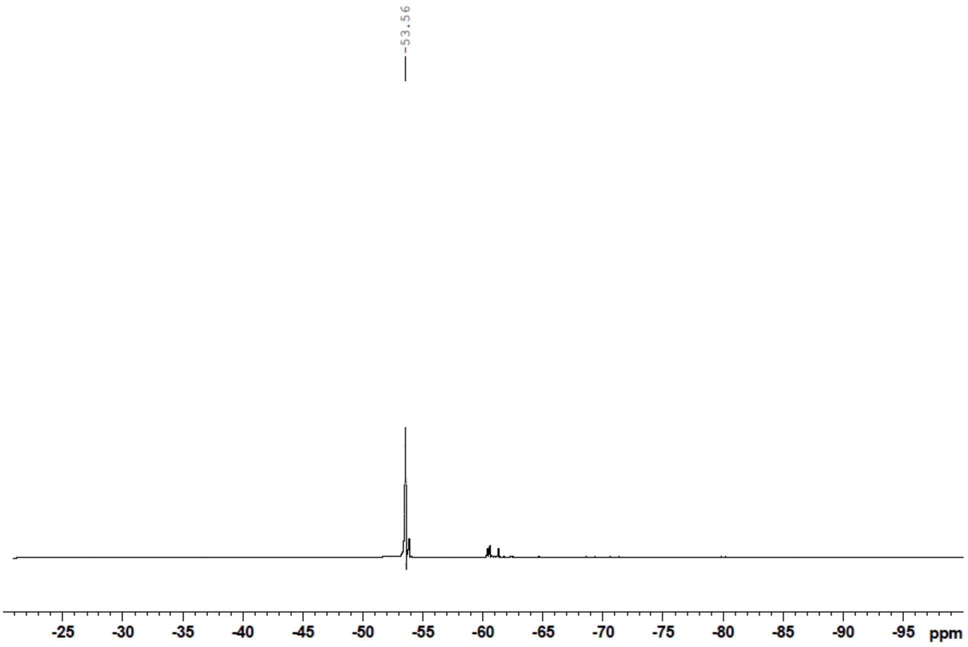

Fig. S55 ^{19}F NMR spectrum (377 MHz, $CDCl_3$) of **4e**.

Table S1. Optimization of the Reaction Conditions for Trifluoromethylation of 1-Dodecene. See the Experimental Details for the Reaction Protocol.

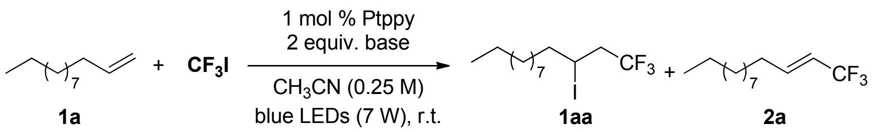

entry	base	solvent	variations	yield (%) **1aa**	yield (%) **2a**
1	DBU	CH_3CN		0	97
2	TMEDA	CH_3CN		80	14
3	DIPEA	CH_3CN		94	trace
4	TEA	CH_3CN		76	trace
5	K_3PO_4	CH_3CN		0	0
6	K_2HPO_4	CH_3CN		0	0
7	KO^tBu	CH_3CN		0	0
8	DBU	DMF		0	80
9	DBU	MeOH		10	70
10	DBU	CH_2Cl_2		0	95
11	DBU	CH_3CN	without Ptppy	0	0
12	DBU	CH_3CN	no light	0	0
13	DBU	CH_3CN	1 mol % $[Ru(bpy)_3]Cl_2$	0	98
14	DBU	CH_3CN	1 mol % $[Ir(ppy)_2(dtbbpy)]PF_6$	0	97

Table S2. Optimization of the Reaction Conditions for Trifluoromethylation of 3-Methylindole. See the Experimental Details for the Reaction Protocol.

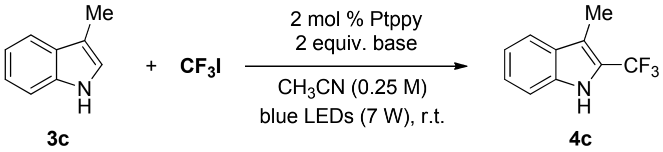

entry	base	variations	yield (%)
1	DBU		32
2	TMEDA		70
3	DIPEA		47
4	TEA		48
5	K_3PO_4		trace
6	K_2HPO_4		trace
7	KO^tBu		trace
8	TMEDA	without Ptppy	25
9	TMEDA	no light	trace
10	TMEDA	1 mol % $[Ru(bpy)_3]Cl_2$	90
11	TMEDA	1 mol % $[Ir(ppy)_2(dtbbpy)]PF_6$	77

Table S3. Optimization of the Reaction Conditions for Trifluoromethylation of *N*-Methylpyrrole. See the Experimental Details for the Reaction Protocol.

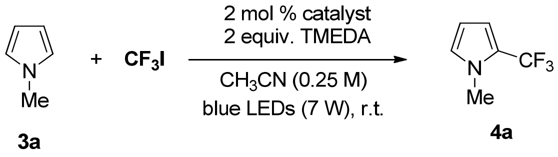

entry	catalyst	variations	yield (%)
1	Ptppy		97
2	PtOMe		84
3	Ptdfppy		71
4	no catalyst		21
5	Ptppy	no light	trace
6	$[Ru(bpy)_3]Cl_2$		97
7	$[Ir(ppy)_2(dtbbpy)]PF_6$		98

Table S4. Report of the Optimized Geometry for the One-Electron Oxidized Species of Ptdfppy

Center Number	Atomic Number	Atomic Type	Coordinates (Angstroms) X	Y	Z
1	78	0	-0.587938	0.060739	-0.000073
2	6	0	1.118402	-0.882702	-0.000046
3	6	0	2.269372	-0.020385	0.000007
4	6	0	1.269649	-2.279660	-0.000069
5	6	0	3.523843	-0.618076	0.000022
6	6	0	2.545465	-2.805015	-0.000052
7	1	0	0.406339	-2.931789	-0.000102
8	6	0	3.688289	-1.999191	-0.000008
9	1	0	4.679205	-2.435271	0.000005
10	6	0	0.192665	2.945280	0.000058
11	6	0	1.980784	1.411007	0.000045
12	6	0	1.067749	4.025242	0.000119
13	1	0	-0.882833	3.065828	0.000035
14	6	0	2.896902	2.462996	0.000108
15	6	0	2.434956	3.776866	0.000145
16	1	0	0.672471	5.032305	0.000146
17	1	0	3.955037	2.251688	0.000129
18	1	0	3.143216	4.596533	0.000195
19	8	0	-2.341559	1.184133	-0.000121
20	8	0	-1.726722	-1.643159	0.000112
21	6	0	-3.000924	-1.718103	0.000155
22	6	0	-4.619966	1.794604	-0.000097
23	1	0	-5.621250	1.367051	-0.000513
24	1	0	-4.501534	2.431536	0.880808
25	1	0	-4.500988	2.432087	-0.880519
26	6	0	-3.546352	-3.117937	0.000329
27	1	0	-4.634621	-3.137732	-0.000059
28	1	0	-3.174944	-3.650124	-0.879634
29	1	0	-3.175619	-3.649603	0.880900
30	6	0	-3.547598	0.743007	-0.000069
31	6	0	-3.890772	-0.617949	0.000049
32	1	0	-4.945321	-0.854684	0.000068
33	7	0	0.640356	1.683499	0.000023
34	9	0	4.638496	0.132613	0.000069
35	9	0	2.718062	-4.129015	-0.000077

Table S5. Report of the Optimized Geometry for the One-Electron Oxidized Species of Ptppy

Center Number	Atomic Number	Atomic Type	Coordinates (Angstroms) X	Y	Z
1	78	0	-0.326236	-0.023842	-0.000043
2	6	0	1.130454	-1.311551	-0.000018
3	6	0	2.446629	-0.730397	0.000008
4	6	0	0.999191	-2.719083	-0.000029
5	6	0	3.567150	-1.548854	0.000021
6	6	0	2.126160	-3.521087	-0.000016
7	1	0	0.007403	-3.153323	-0.000048
8	6	0	3.406188	-2.939453	0.000008
9	1	0	4.567965	-1.134224	0.000040
10	1	0	2.029630	-4.600322	-0.000024
11	1	0	4.283439	-3.575669	0.000018
12	6	0	1.073380	2.627049	0.000028
13	6	0	2.469785	0.732862	0.000024
14	6	0	2.169718	3.480751	0.000059
15	1	0	0.052027	2.985285	0.000016
16	6	0	3.598154	1.549022	0.000057
17	6	0	3.448293	2.932308	0.000074
18	1	0	2.012375	4.551147	0.000072
19	1	0	4.584829	1.105796	0.000069
20	1	0	4.321494	3.573381	0.000099
21	8	0	-1.784722	1.467685	-0.000048
22	8	0	-1.806224	-1.440981	0.000041
23	6	0	-3.066462	-1.223049	0.000069
24	6	0	-3.877467	2.553609	-0.000019
25	1	0	-4.947660	2.353402	-0.000303
26	1	0	-3.623741	3.149804	0.880853
27	1	0	-3.623301	3.150103	-0.880556
28	6	0	-3.909565	-2.467827	0.000136
29	1	0	-4.974938	-2.244687	-0.000145
30	1	0	-3.666417	-3.068823	-0.880240
31	1	0	-3.666825	-3.068424	0.880903
32	6	0	-3.058304	1.293896	-0.000010
33	6	0	-3.689901	0.042712	0.000029
34	1	0	-4.770592	0.043481	0.000033
35	7	0	1.226835	1.296238	0.000011

Table S6. Report of the Optimized Geometry for the One-Electron Oxidized Species of PtOMe

Center Number	Atomic Number	Atomic Type	Coordinates (Angstroms) X	Y	Z
1	78	0	-0.710858	-0.035674	-0.000019
2	6	0	1.168999	-0.512400	-0.000015
3	6	0	2.080288	0.603987	0.000000
4	6	0	1.709017	-1.822844	-0.000025
5	6	0	3.439112	0.398064	0.000003
6	6	0	3.069526	-2.036866	-0.000021
7	1	0	1.025730	-2.663155	-0.000038
8	6	0	3.949253	-0.925824	-0.000005
9	6	0	-0.679376	2.960054	0.000012
10	6	0	1.428797	1.919729	0.000011
11	6	0	-0.096978	4.220650	0.000029
12	1	0	-1.751085	2.807410	0.000007
13	6	0	2.058281	3.160597	0.000027
14	6	0	1.290841	4.321021	0.000036
15	1	0	-0.727808	5.099699	0.000035
16	1	0	3.138544	3.219437	0.000033
17	1	0	1.772318	5.291362	0.000048
18	8	0	-2.694144	0.636763	-0.000015
19	8	0	-1.361974	-1.972303	-0.000036
20	6	0	-2.589530	-2.349178	-0.000033
21	6	0	-5.048181	0.620812	0.000040
22	1	0	-5.902644	-0.053992	-0.000395
23	1	0	-5.100628	1.266544	0.880953
24	1	0	-5.100306	1.267343	-0.880296
25	6	0	-2.764721	-3.843833	0.000114
26	1	0	-3.813271	-4.136511	-0.000768
27	1	0	-2.272586	-4.265816	-0.880229
28	1	0	-2.274277	-4.265387	0.881624
29	6	0	-3.735580	-0.113624	-0.000030
30	6	0	-3.718717	-1.515595	-0.000046
31	1	0	-4.678946	-2.011132	-0.000055
32	7	0	0.067458	1.846581	0.000004
33	1	0	4.159469	1.206776	0.000014
34	1	0	3.460520	-3.045278	-0.000035
35	8	0	5.273826	-1.014180	0.000001
36	6	0	5.932515	-2.297257	0.000035
37	1	0	5.671077	-2.859100	-0.898687
38	1	0	6.994738	-2.069744	0.000114
39	1	0	5.670948	-2.859125	0.898703

References

1. Wu, W., W. Wu, S. Ji, H. Guo, J. Zhao, *Dalton Trans.* **2011**, *40*, 5953–5963.
2. H. Woo, S. Cho, Y. Han, W.-S. Chae, D.-R. Ahn, Y. You, W. Nam, *J. Am. Chem. Soc.* **2013**, *135*, 4771−4787.
3. H. J. Kuhn, S. E. Braslavsky, R. Schmidt, *Pure. Appl. Chem*. **2004**, *76*, 2105−2146.
4. Y. Ye, M. S. Sanford, *J. Am. Chem. Soc*. **2012**, *134*, 9034−9037.
5. M. J. Frisch, G. W. Trucks, H. B. Schlegel, G. E. Scuseria, Robb, M. A.; J. R. Cheeseman, G. Scalmani, V. Barone, B. Mennucci, G. A. Petersson, H. Nakatsuji, M. Caricato, X. Li, H. P. Hratchian, A. F. Izmaylov, J. Bloino, G. Zheng, J. L. Sonnenberg, M. Hada, M. Ehara, K. Toyota, R. Fukuda, J. Hasegawa, M. Ishida, T. Nakajima, Y. Honda, O. Kitao, H. Nakai, T. Vreven, J. A. Montgomery, Jr., J. E. Peralta, F. Ogliaro, M. Bearpark, J. J. Heyd, E. Brothers, K. N. Kudin, V. N. Staroverov, R. Kobayashi, J. Normand, K. Raghavachari, A. Rendell, J. C. Burant, S. S. Iyengar, J. Tomasi, M. Cossi, N. Rega, J. M. Millam, M. Klene, J. E. Knox, J. B. Cross, V. Bakken, C. Adamo, J. Jaramillo, R. Gomperts, R. E. Stratmann, O. Yazyev, A. J. Austin, R. Cammi, C. Pomelli, J. W. Ochterski, R. L. Martin, K. Morokuma, V. G. Zakrzewski, G. A. Voth, P. Salvador, J. J. Dannenberg, S. Dapprich, A. D. Daniels, O.

Farkas, J. B. Foresman, J. V. Ortiz, J. Cioslowski, D. J. Fox, Gaussian, Inc., Wallingford CT, 2009.

6. A. D. Becke, *J. Chem. Phys.* **1988**, *88*, 2547–2553.
7. A. D. Becke, *J. Chem. Phys.* **1993**, *98*, 5648–5652.
8. A. D. Becke, *Phys. Rev. A* **1988**, *38*, 3098–3100.
9. N. M. O'Boyle, A. L. Tenderholt, K. M. Langner, *J. Comp. Chem.* **2008**, *29*, 839–845.
10. S. I. Gorelsky, AOMix: Program for Molecular Orbital Analysis, http://www.sg-chem.net/, University of Ottawa, version 6.5, 2011.
